# A Length-Aware C-Terminal Rule for Prioritizing Short ACE-Inhibitory Peptides from Food Protein Hydrolysates

**DOI:** 10.3390/foods15152764

**Published:** 2026-08-06

**Authors:** Mei-Ling Li, Ying-Jang Lai, Pei-Yu Wu, Jen-Chieh Li, Shang-Ming Huang, Kuo-Chiang Hsu

**Affiliations:** 1 Department of Nutrition, China Medical University, No. 100, Sec.1, Jingmao Rd., Beitun Dist., Taichung 406, Taiwan; codarduck@gmail.com (M.-L.L.); peiyuwu@mail.cmu.edu.tw (P.-Y.W.);; 2 Department of Food Science, National Quemoy University, No. 1, University Rd., Jinning Township, Kinmen 892, Taiwan; lai@nqu.edu.tw; 3 Department of Food Nutrition and Health Biotechnology, Asia University, 500 Lioufeng Rd., Wufeng, Taichung 413, Taiwan

**Keywords:** ACE-inhibitory peptides, structure–activity rules, in silico screening, external benchmarking, applicability domain, molecular dynamics simulation, protein hydrolysates

## Abstract

The discovery of angiotensin-converting enzyme (ACE)-inhibitory peptides from food protein hydrolysates is commonly guided by empirical fractionation or sequence-based prediction, but few screening rules have been evaluated at the hydrolysate level and independently benchmarked across peptide lengths. Here, we developed a length-aware C-terminal screening rule (Rule 5: P1’ ∈ {W, Y, F, P} and P2’ ∈ {L, I, V, K, R, H}) through an experimentally anchored framework. The rule was derived using 24 stratified protein–protease hydrolysates and showed the strongest associations with ACE inhibition (r = 0.711, *p* < 0.001) and log_10_(1/IC_50_) (r = 0.741, *p* < 0.001) among five evaluated rules. Independent evaluation in 16 composition-weighted commercial hydrolysates retained predictive utility (r = 0.608 for ACE inhibition and r = 0.581 for log_10_(1/IC_50_)). Five peptides—VF, GIF, LP, IP, and VP—were selected because they represented the intersection of in silico cleavage prediction, Rule 5 compliance, and corresponding candidate-associated low-mass MALDI features in the experimentally prepared hydrolysates. All five inhibited ACE (IC_50_ = 19.50–93.19 µM); VF and GIF showed mixed-type inhibition, whereas LP, IP, and VP showed competitive inhibition. External benchmarking against 1429 quantitative ACE-inhibitory peptides established a defined applicability domain: Rule 5 significantly enriched potent peptides among di- and tripeptides (2–3 residues; median IC_50_, 28.0 vs. 79.0 µM; *p*_adj_ < 0.001, Benjamini–Hochberg-corrected; enrichment factor = 2.5 at IC_50_ ≤ 1 µM), but enrichment attenuated rapidly as longer sequences were included. Molecular dynamics simulations (200 ns) showed persistent peptide–ACE contact for all five candidates under the simulated conditions. Rule 5 is therefore proposed as a transparent first-pass filter for prioritizing short ACE-inhibitory candidates and protein–protease combinations, rather than as a universal predictor across the full peptide-length spectrum.

## 1. Introduction

Hypertension is a major modifiable risk factor for cardiovascular disease and remains one of the most prevalent non-communicable disorders worldwide. Angiotensin-converting enzyme (ACE; EC 3.4.15.1) contributes to blood-pressure regulation by generating the vasoconstrictor angiotensin II and degrading the vasodilator bradykinin [1,2]. Although synthetic ACE inhibitors are clinically effective, adverse effects such as persistent cough, hypotension, and angioedema have sustained interest in food-derived ACE-inhibitory peptides as ingredients for functional foods [3,4].

Food-derived ACE inhibitors are frequently short peptides released during enzymatic hydrolysis of dietary proteins [5]. Their activity is strongly influenced by the C-terminal region: aromatic or proline residues at the terminal position (designated here as P1’) and hydrophobic or positively charged residues at the penultimate position (P2’) are repeatedly favored in kinetic and quantitative structure–activity relationship studies [6,7,8,9]. These observations provide a useful biochemical basis for sequence prioritization, but they do not by themselves establish whether a simple motif can predict the inhibitory performance of complex food protein hydrolysates.

Current discovery workflows commonly begin with broad hydrolysis, activity-guided fractionation, and repeated purification before peptide identification. Although effective, this strategy is resource-intensive and poorly suited to screening large numbers of protein sources and proteases. Computational digestion and sequence-based filters could reduce this search space; however, many published models are evaluated primarily within peptide datasets used for model construction, and their practical ability to rank real hydrolysates is seldom tested.

A further complication is peptide length. The C-terminal dipeptide can dominate the interaction of di- and tripeptides with ACE, whereas longer peptides possess additional residues that may introduce alternative contacts, conformational constraints, or steric effects. A motif that is useful for short peptides should therefore not be assumed to operate as a universal classifier across the entire peptide-length spectrum. Defining the applicability domain is essential if a simple rule is to be used as a transparent first-pass screening tool rather than overextended as a general potency model.

In previous work, we showed that the abundance of preferred N-terminal motifs generated by virtual hydrolysis could predict dipeptidyl peptidase-IV inhibitory activity of protein hydrolysates [10,11]. Building on that concept, the present study developed an ACE-focused framework that integrates literature-derived C-terminal rules, virtual hydrolysis, hydrolysate-level experimentation, targeted peptide verification, external peptide-database benchmarking, and structural simulation. The central objective was not merely to identify several active peptides, but to determine whether a concise and interpretable rule could prioritize productive protein–protease combinations and short peptide candidates.

Specifically, we aimed to: (1) compare published C-terminal structure–activity rules using a stratified set of experimentally prepared hydrolysates; (2) derive a parsimonious rule and evaluate it independently in composition-weighted commercial protein hydrolysates; (3) examine peptides located at the intersection of in silico cleavage prediction, rule compliance, and mass-spectrometric screening, followed by peptide synthesis, ACE inhibition, and kinetic analysis; and (4) define the peptide-length applicability domain by external benchmarking against 1429 quantitative ACE-inhibitory peptides, with docking and molecular dynamics used only as supportive structural interpretation.

## 2. Materials and Methods

### 2.1. Materials and Reagents

Nine protein substrates were selected for enzymatic hydrolysis, comprising seven purified proteins and two commercial food ingredients. β-Lactoglobulin A (≥90% purity, from bovine milk, Cat. No. L7880), κ-casein (≥70%, from bovine milk, Cat. No. C0406), α-lactalbumin (≥85%, from bovine milk, Cat. No. L5385), lactoferrin (≥85%, from bovine milk, Cat. No. L9507), myoglobin (≥90%, from equine skeletal muscle, Cat. No. M1882), gliadin (from wheat, Cat. No. G3375), and gluten (≥75% protein basis, from wheat, Cat. No. G5004) were purchased from Sigma-Aldrich (St. Louis, MO, USA). Beef (bovine shoulder tender) was purchased from Sun Make (Taipei, Taiwan), and high-protein milk powder (S-P93) was obtained from Sentosa (Taipei, Taiwan); both were stored at −20 °C until use.

Seven proteolytic enzymes with distinct cleavage specificities were employed for hydrolysis. Trypsin (≥10,000 BAEE units/mg protein, from bovine pancreas, Type I, Cat. No. T1426), pepsin (≥3200 units/mg protein, from porcine gastric mucosa, Cat. No. P6887), thermolysin (30–350 units/mg protein, from *Geobacillus stearothermophilus*, Cat. No. P1512), papain (≥10 units/mg protein, from papaya latex, Cat. No. P4762), bromelain (≥3 units/mg protein, from pineapple stem, Cat. No. B4882), α-chymotrypsin (≥40 units/mg protein, from bovine pancreas, Type II, Cat. No. C4129), and proline specific endopeptidase (prolyl oligopeptidase, PEP; ≥5.0 units/mg solid, from *Flavobacterium* sp., Cat. No. E1411) were also obtained from Sigma-Aldrich (St. Louis, MO, USA). All other chemicals and reagents were of analytical grade.

### 2.2. Structure–Activity Rule Development and Hydrolysate-Level Evaluation

**Figure 1:**
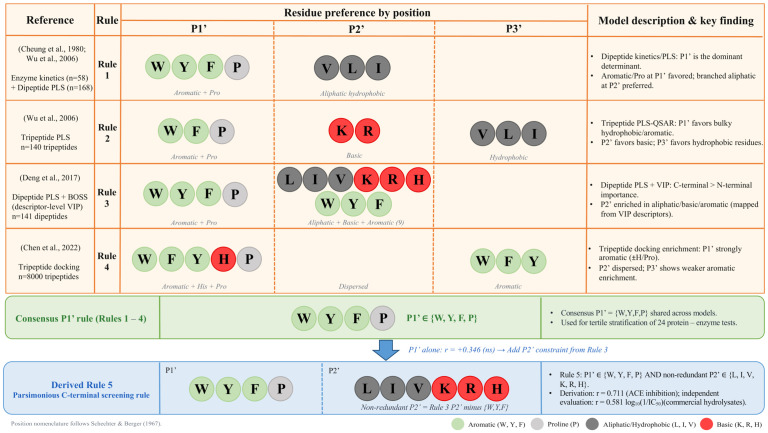
Residue preferences at P1’/P2’ (and P3’ for tripeptides) summarized from four published structure–activity models for ACE-inhibitory peptides and derivation of Rule 5. A consensus P1’ constraint {W, Y, F, P} was shared across the four models and used for tertile stratification of the 24-hydrolysate rule-development set. Rule 5 combines this P1’ set with a simplified P2’ constraint {L, I, V, K, R, H} derived from Rule 3 after excluding the aromatic P2’ residues W, Y, and F. Rule 1 (enzyme kinetics and dipeptide PLS, n = 58/168) [6,7]; Rule 2 (tripeptide PLS, n = 140) [7]; Rule 3 (dipeptide QSAR and variable-importance analysis, n = 141) [8]; Rule 4 (docking enrichment among 8000 tripeptides) [9]. Rule 5 showed r = 0.711 with ACE inhibition in the derivation set and r = 0.581 with log_10_(1/IC_50_) in the independent commercial-hydrolysate evaluation. See Table S5 for derivation-set comparisons and Table S16 for the independent commercial-hydrolysate evaluation.

The study was organized as a sequential rule-development and evaluation framework: (i) extraction of C-terminal preferences from four published structure–activity models; (ii) virtual hydrolysis of food proteins and P1’-frequency stratification; (iii) rule derivation using 24 experimentally prepared protein–protease hydrolysates; and (iv) independent hydrolysate-level evaluation using 16 composition-weighted commercial protein preparations. External peptide-level benchmarking and structural simulations were subsequently used to define and interpret the applicability domain. The workflow is summarized in Figure 1.

#### 2.2.1. Literature-Derived Structural Rules for ACE-Inhibitory Peptides (Rules 1–4)

Four published structure–activity relationship models that address the structural requirements of ACE-inhibitory peptides were systematically reviewed to identify consensus positional preferences (Figure 1). The models differ in peptide chain length, statistical methodology, and descriptor set, yet converge on a shared finding: the C-terminal residue is the single most important determinant of ACE-inhibitory activity. For clarity, the C-terminal residue of each peptide is herein designated as the P1’ position, and the penultimate C-terminal residue as P2’, following the convention commonly adopted in ACE-inhibitory peptide QSAR studies [7], rather than the original protease-cleavage-site definition of Schechter–Berger nomenclature [12].

**Rule 1:** P1’ in {W, Y, F, P} and P2’ in {V, L, I}, derived from enzyme kinetics and PLS-QSAR of 168 dipeptides (**R^2^ = 73.2**%) [6,7].

**Rule 2** represents the tripeptide sub-model: P1’ in {W, F, P} (bulky hydrophobic), P2’ in {K, R} (positively charged), and P3’ in {V, L, I} (hydrophobic aliphatic). This tripeptide rule is presented separately from Rule 1 because it introduces explicit constraints on both P2’ and P3’ positions. However, the stringent three-position requirement limits the number of qualifying fragments in typical hydrolysates.

**Rule 3** was derived from a multi-descriptor QSAR model for 141 ACE-inhibitory dipeptides (R^2^ = 0.734, Q^2^ = 0.715) [8]. The resulting P2’ constraint included hydrophobic (L, I, V), positively charged (K, R, H), and aromatic (W, Y, F) residues, expanding the P2’ set to {L, I, V, K, R, H, W, Y, F}.

**Rule 4** applied molecular docking to screen 8000 tripeptides against ACE [9]. Frequency analysis of the top 500 ranked tripeptides revealed enrichment of aromatic residues at P1’ and P3’, and hydrophobic residues (H, P) at P2’, yielding the constraint P1’ in {W, F, Y, H, P}, P3’ in {W, F, Y}.

Based on the consensus of Rules 1–4, the shared P1’ criterion was defined as P1’ in {W, Y, F, P}, consistently favored across all four models. P was included because it appeared in three of four rules and is the most frequently reported C-terminal residue among known ACE-inhibitory peptides in the BIOPEP-UWM database [13].

#### 2.2.2. Virtual Hydrolysis and P1’ Frequency Screening Using BIOPEP-UWM

To systematically identify food protein–enzyme combinations with high structural potential for generating ACE-inhibitory peptides, in silico virtual hydrolysis was performed using the BIOPEP-UWM database (https://biochemia.uwm.edu.pl/en/biopep-uwm/) accessed on 13 December 2023 [13]. A total of 773 food protein sequences are cataloged in BIOPEP-UWM (accessed October to December 2024). From these, 84 commercially available protein ingredients were selected as the initial substrate pool, chosen based on accessibility from established biochemical suppliers (see Section 2.1; Table S1), ensuring reproducibility and batch consistency for comparative screening purposes. The 84 proteins span dairy, cereal, meat, and other food categories, covering a broad compositional diversity.

Each of the 84 proteins was subjected to virtual enzymatic digestion under eight proteolytic conditions representing seven enzymes (pepsin was evaluated at two pH levels): trypsin (EC 3.4.21.4), pepsin at pH 1.3 (EC 3.4.23.1), pepsin at pH > 2.0 (EC 3.4.23.1), thermolysin (EC 3.4.24.27), papain (EC 3.4.22.2), stem bromelain (EC 3.4.22.32), α-chymotrypsin (EC 3.4.21.1), and prolyl endopeptidase (PEP; EC 3.4.21.26). The eight conditions were implemented using the built-in enzyme action module of BIOPEP-UWM (Table S2).

For each protein–enzyme combination, the P1’ frequency was calculated as the proportion of predicted peptide fragments (length ≥ 2 amino acids) carrying a C-terminal residue belonging to the consensus ACE-inhibitory set {W, Y, F, P} (Equation (1)):

(1) $$P1’\;\mathrm{frequency}\;(\%)\;=\;\frac{N_{peptides\;with\;P1’\;\in \;\{W,Y,F,P\}}}{N_{total\;peptide\;fragments\;(length\;\geq \;2)}}\;\times \;100$$

Single amino acids released during virtual hydrolysis were excluded from the denominator, as they lack a P2’ residue necessary for engaging the ACE S2’ subsite. The resulting 672 protein–enzyme combinations (84 proteins × 8 proteolytic conditions) were ranked by P1’ frequency within each enzyme group. Combinations yielding zero peptide fragments were excluded. The remaining combinations were stratified into four categories: High (H; top tertile; frequency: 100–32.84%, n = 197), Medium (M; middle tertile; frequency: 32.58–13.08%, n = 197), Low (L; bottom tertile; frequency: 13.01–0.36%, n = 197), and Zero (Z; no cleavage or all single amino acids; n = 81).

#### 2.2.3. Selection of the 24-Hydrolysate Rule-Development Set

Twenty-four protein–protease combinations were selected by stratified sampling from the High, Medium, and Low P1’-frequency tertiles (eight per tertile; Table S3). The purpose of this sampling was to construct a rule-development set spanning a broad range of predicted motif abundance and experimental activity while retaining diversity in protein source and protease class. Zero-frequency combinations were not included because they could not contribute to comparison among C-terminal rules.

ACE inhibition was measured for each hydrolysate at 0.5 mg mL^−1^ using the FAPGG assay, and IC_50_ values were determined independently. Correlations between motif frequency and either ACE inhibition or log_10_(1/IC_50_) were used to compare the literature-derived rules. Because Rule 5 was refined after examining this 24-hydrolysate set, these samples are referred to throughout as the derivation set, not as an independent validation set.

#### 2.2.4. Derivation and Internal Evaluation of Rule 5

Within the derivation set, Rule 3 produced the strongest correlations among the four literature-derived rules (Table S5). Its P2’ set, however, included W, Y, and F—residues that overlap with the P1’ aromatic criterion. This overlap means that for peptides already carrying an aromatic C-terminal residue, the aromatic P2’ residues contribute relatively little additional selectivity to the two-position filter. Excluding them from P2’ was therefore explored as an empirical model-simplification step. This simplification is also consistent with the known geometry of the ACE S2’ subsite, a hydrophobic pocket whose dimensions favor branched aliphatic side chains (Leu, Ile, Val) and extended positively charged residues (Lys, Arg, His) rather than planar aromatic ring systems [6,7].

Rule 5 was derived from Rule 3 by excluding aromatic residues from P2’, yielding a simplified motif with P1’ in {W, Y, F, P} and P2’ in {L, I, V, K, R, H}. This simplification was guided by both the empirical performance within the derivation set and the structural plausibility described above; Rule 5 should therefore be regarded as a parsimonious variant of Rule 3 rather than as the uniquely correct representation of S2’ binding preferences.

Rule 5: P1’ in {W, Y, F, P} and P2’ in {L, I, V, K, R, H}.

Rule 5 yielded the highest correlations in the derivation set with ACE inhibition (r = 0.711, *p* < 0.001) and log_10_(1/IC_50_) (r = 0.741, *p* < 0.001; Table S5). These values were treated as internal rule-development performance; independent predictive utility was assessed subsequently using the 16 commercial hydrolysates and the external peptide database.

Extension to a third position (P3’) was explored but did not improve predictive accuracy.

Rule 5 was adopted for all subsequent analyses.

For each protein–enzyme combination, the Rule k-compliant peptide frequency was calculated using Equation (2):

(2) $$\mathrm{Rule}\;k\;\mathrm{frequency}\;(\%)\;=\;\frac{N_{rule\;k}}{N_{total}}\;\times \;100$$

where N_rule k_ is the number of peptide fragments (≥2 aa) satisfying Rule k, and N_total_ is the total number of fragments (≥2 aa) from virtual hydrolysis. The positional assignments follow the ACE-QSAR convention established by Wu et al. [7] and Cheung et al. [6]: P1’ denotes the C-terminal residue and P2’ the penultimate residue of the inhibitory peptide. This convention differs from the original Schechter–Berger protease-cleavage nomenclature [12], in which P1’ refers to the first residue on the leaving-group side of the scissile bond; a schematic comparing the two conventions is provided in Figure S5.

### 2.3. Preparation of Protein Hydrolysates

Proteins (gluten, gliadin, α-lactalbumin, β-lactoglobulin, κ-casein, lactoferrin, myoglobin, beef and milk powder) were dissolved or dispersed individually in distilled deionized water (ddH_2_O) at 4% (*w*/*v*). Where applicable, lipid removal was performed prior to hydrolysis: lyophilized beef was defatted by three successive hexane extractions (1:10, *w*/*v*; 4 °C, 10,000× *g*, 10 min), followed by vacuum evaporation of residual solvent under reduced pressure for 30 min and freeze dry.

For each protein–protease combination, three independent hydrolysis batches were prepared. Protein substrates (1 g per batch) were hydrolyzed at a 3% (*w*/*w*) enzyme-to-substrate ratio under optimal conditions for each protease (pH 8.0/37 °C for trypsin, pH 6.2/50 °C for papain, pH 8.0/70 °C for thermolysin, pH 1.3 or 2.0/37 °C for pepsin, pH 8.0/50 °C for bromelain, pH 8.0/37 °C for α-chymotrypsin, and pH 7.0/35 °C for proline-specific endopeptidase). Hydrolysis was allowed to proceed to plateau, as determined by TNBS monitoring of free amino groups (Section 2.4); endpoint times ranged from 0.33 to 4.0 h depending on the enzyme–substrate combination. The pH was maintained at the target value by addition of 1 M NaOH or 1 M HCl as needed throughout hydrolysis. The individual hydrolysis endpoint time and corresponding final DH are reported in Table 1. Aliquots were heat-inactivated (100 °C, 15 min), centrifuged (12,100× *g*, 10 min, 4 °C), lyophilized, and stored at −20 °C.

### 2.4. Degree of Hydrolysis

The degree of hydrolysis (DH) plateau time for each combination was used to define the endpoint for subsequent experiments, consistent with the in silico prediction assuming complete theoretical hydrolysis (Figure S1).

The DH was determined by the TNBS (2,4,6-trinitrobenzene sulfonic acid) colorimetric method according to Adler-Nissen [14] with minor modifications [15], using L-leucine as the standard. Absorbance was measured at 340 nm and DH was calculated using Equation (3):

(3) $$\mathrm{DH}\;(\%)\;=\;\frac{L_{t}-L_{0}}{L_{max}-L_{0}}\;\times \;100$$

where L_t_ is the free amino group content at hydrolysis time t; L_0_ is the free amino group content at time zero; and L_max_ is the total free amino group content obtained after complete acid hydrolysis (6 N HCl, 110 °C, 24 h).

### 2.5. ACE-Inhibitory Activity Assay

ACE-inhibitory activity was measured using N-[3-(2-furyl)acryloyl]-Phe-Gly-Gly (FAPGG; 0.5 mM final concentration) as substrate according to Udenigwe et al. [16] with minor modifications. ACE from rabbit lung (Sigma-Aldrich, Cat. No. A6778; 2 mU per well) was pre-incubated with the test sample (20 µL sample + 20 µL enzyme in a total reaction volume of 200 µL) for 10 min at 37 °C before initiating the reaction with 160 µL FAPGG solution. The rate of FAPGG hydrolysis (ΔA340 nm/min) was monitored continuously for 20 min at 37 °C in 80 mM HEPES-NaOH buffer (pH 8.3, 300 mM NaCl) using a BioTek Synergy HT Multi-Mode Microplate Reader (BioTek Instruments, Winooski, VT, USA). A sample blank (sample + buffer, no enzyme) and a positive control (captopril, 1 nM) were included in each plate. ACE inhibition was calculated using Equation (4):

(4) $$\mathrm{ACE}\;\mathrm{inhibition}\;(\%)\;=\;\frac{{Slope}_{blank}-{Slope}_{sample}}{{Slope}_{blank}}\;\times \;100$$

IC_50_ values (concentration yielding 50% inhibition) were determined by non-linear regression using the Hill equation fitted to inhibition–concentration data collected over various concentrations. For hydrolysates, values represent three independently prepared hydrolysis batches; for synthesized peptides, values represent three independent assay replicates (mean ± SD, n = 3).

### 2.6. MALDI-TOF/TOF Analysis and Peptide Assignment

Peptide identification in selected hydrolysates was performed by matrix-assisted laser desorption/ionization tandem time-of-flight mass spectrometry (MALDI-TOF/TOF; Bruker UltraFlex III, Bruker Daltonics, Bremen, Germany) at the Instrumentation Center, China Medical University (Table S6). Lyophilized hydrolysates were reconstituted in 0.1% formic acid to a final concentration of 2000 µg mL^−1^. Twenty-four hydrolysate samples were analyzed.

Full-scan spectra (*m*/*z* 200–2000) were acquired, and selected high-intensity ions were subjected to CID-MS/MS. Sequence assignments were generated using FlexAnalysis 3.3 and BioTools 3.2 and cross-referenced with peptides predicted from the corresponding BIOPEP-UWM digestion. Assignments were considered putative when isomeric sequences could not be distinguished unambiguously by mass spectrometry alone. In particular, LP and IP have identical elemental compositions and precursor masses. They were therefore retained as alternative isomeric candidates based on protease-specific in silico digestion, but neither the individual identity nor the simultaneous presence of both peptides could be established from the MALDI data.

### 2.7. Selection, Synthesis, and Purity of Candidate Peptides

Five peptides (VF, GIF, LP, IP, and VP) were selected for synthesis because they satisfied three independent criteria: (i) generation predicted by virtual proteolysis, (ii) compliance with Rule 5, and (iii) presence of a corresponding candidate-associated feature in the low-mass MALDI region of the experimentally prepared hydrolysate. Selection was therefore intended to test sequences prioritized through the predefined intersection-based workflow in relation to experimentally prepared food protein hydrolysates, rather than to claim unambiguous MALDI-TOF/TOF identification. Peptides were synthesized by Kelowna International Scientific Inc. (Taipei, Taiwan) at ≥95% purity by a commercial service, and identity and purity were confirmed by analytical RP-HPLC and ESI-MS before biochemical testing.

### 2.8. Enzyme Kinetics

Inhibition kinetics of the five synthetic peptides against ACE were determined by Lineweaver–Burk double-reciprocal analysis. FAPGG substrate concentrations of 0.1, 0.2, 0.5, 0.75, 1.0, 1.5, 2.0, 3.0, and 4.0 mM were used in the presence of four inhibitor concentrations (0, 10, 25, and 50 µM), with the assay conducted as described in Section 2.5. Initial velocity (V, nmol min^−1^ mg protein^−1^) was determined from the linear rate of absorbance decrease at 340 nm. Double-reciprocal plots of 1/V versus 1/[S] were constructed. The y-intercept and slope corresponded to 1/V_max_ and K_m_/V_max_, respectively; therefore, V_max_ was calculated as the reciprocal of the y-intercept, and K_m_ as the slope divided by the y-intercept. Data are expressed as mean ± SD (n = 3). Inhibition constants were obtained from secondary replots of the fitted apparent kinetic parameters versus inhibitor concentration. Competitive inhibition models were applied to LP, IP, and VP, whereas mixed-inhibition models were applied to VF and GIF, yielding K_i_ and K_i_’ where applicable.

### 2.9. Molecular Docking

Molecular docking simulations were performed using AutoDock Vina v1.2.7 [17,18], and the parameters and grid box coordinates are provided in Table S7. The crystal structure of human testicular ACE (tACE, C-domain) in complex with lisinopril (PDB: 1O86, 2.0 Å resolution) [19] was retrieved from the RCSB Protein Data Bank.

Receptor preparation was performed using the Open Babel 3.1 API [20]. Lisinopril (LPR) and crystallographic water molecules were removed, while the catalytic zinc ion (Zn^2+^) and both chloride ions (CL1, CL2) were retained [21]. Polar hydrogen atoms were added at pH 7.4, Gasteiger partial charges were assigned, and the receptor was exported in PDBQT format.

Three-dimensional peptide structures were generated using PeptideBuilder [22] and energy-minimized with the MMFF94 force field using Open Babel 3.1 [20]. Structures were converted to PDBQT format using Meeko [23] with RDKit [24] for molecular handling (random seed = 42); Open Babel was used as fallback where Meeko conversion was unsuccessful.

Docking was evaluated at three regions of ACE: Site 1 (catalytic active site), Site 2 (CL1 distal pocket), and Site 3 (CL2 proximal pocket). For each peptide, the best-ranked pose at each site was retained for analysis.

Docking scores, site accessibility, and length-normalized scores were compared between the five short Rule 5 candidates and the longer comparison peptides to explore the effects of peptide length on docking behavior. Because the two groups differed in peptide length, these comparisons were treated as exploratory rather than as motif-specific validation. AutoDock Vina scores, originally reported in kcal mol^−1^, were converted to kJ mol^−1^ by multiplying by 4.184 for presentation.

Docking grid parameters for all three sites (center coordinates, dimensions, and key residues) are provided in Table S7.

### 2.10. External Peptide-Database Benchmarking

To evaluate Rule 5 beyond the derivation hydrolysates, external benchmarking was performed using 1429 unique ACE-inhibitory peptides with quantitative IC_50_ values (in µM) compiled from three public databases: the Antihypertensive Peptide Database (AHTPDB [25]), the BIOPEP-UWM database [13], and the Milk Bioactive Peptide Database (MBPDB) [26]. Database curation involved 12-step quality control: removal of entries without numeric IC_50_ values, unit standardization to µM, exclusion of entries reported in mg mL^−1^ or mM without molecular weight information, deduplication across databases using median IC_50_ for sequences appearing multiple times, and A/B class reclassification corrections. The complete curation pipeline and final dataset are provided in Dataset S1 (https://doi.org/10.5281/zenodo.21708604).

Each peptide was classified as Rule 5-compliant or non-compliant based on its C-terminal dipeptide motif. To define the applicability domain of Rule 5, length-stratified analyses were performed across all pairwise length windows (lower bound 2–10 aa × upper bound 2–15 aa). For each window, IC_50_ distributions of compliant versus non-compliant peptides were compared using the Mann–Whitney U test (two-tailed, normal approximation). Enrichment factors (EF) were calculated as the ratio of the proportion of potent peptides (at a given IC_50_ threshold) among Rule 5-compliant peptides to the corresponding proportion in the full unscreened set at each length window. A cumulative inclusion analysis was also performed by fixing the lower bound at 2 aa and progressively increasing the upper bound from 2 to 15 aa to identify the length threshold at which Rule 5 enrichment becomes non-significant. Enrichment factor and sensitivity across the evaluated potency thresholds are shown in Figure S4.

### 2.11. Molecular Dynamics Simulations

To assess peptide–ACE contact persistence beyond static docking, 200 ns all-atom molecular dynamics (MD) simulations were performed for all five Rule 5-compliant peptides (VF, GIF, LP, IP, VP) in complex with human testicular ACE (PDB: 1O86). Simulations were carried out using GROMACS 2025.2 [27] with the AMBER ff99SB-ILDN force field [28] and TIP3P water model [29]. The catalytic Zn^2+^ was retained and parameterized as a non-bonded divalent cation with AMBER ff99SB-ILDN Lennard-Jones parameters (σ = 0.1955 nm, ε = 0.05230 kJ mol^−1^); coordination geometry was maintained by the surrounding His, Glu, and water ligands throughout all simulations, as verified by Zn–Nε(His) distance monitoring. Each complex was solvated in a dodecahedral box with a minimum 1.0 nm distance from the protein to the box edge, and neutralized with Na^+^/Cl^−^ ions to 0.15 M. Energy minimization (steepest descent, 50,000 steps) was followed by 100 ps NVT equilibration (300 K, V-rescale thermostat, τT = 0.1 ps) and 100 ps NPT equilibration (1 bar, Parrinello–Rahman barostat, τP = 2.0 ps). Production runs of 200 ns were performed with a 2 fs time step, using PME electrostatics (cutoff 1.2 nm), LINCS constraints on all bonds with hydrogen, and coordinates saved every 100 ps. GPU acceleration was employed for both non-bonded interactions and PME calculations.

Trajectory analysis was performed after periodic boundary condition (PBC) correction (gmx trjconv, -pbc nojump followed by -pbc mol -center). Backbone RMSD was calculated for the ACE chain only (residues 1–566, excluding the peptide) to assess protein structural stability. The minimum distance between ACE and peptide heavy atoms was monitored to evaluate contact persistence. All metrics were averaged over the equilibrated period (20–200 ns) and reported as mean ± SD.

### 2.12. Statistical Analysis

All data are expressed as mean ± standard deviation (SD). Pearson correlation analysis was used to evaluate associations between in silico rule-compliant peptide-frequency metrics and experimental ACE inhibition or log_10_(1/IC_50_) in both the derivation dataset (n = 24) and the independent commercial-hydrolysate evaluation dataset (n = 16). Dependent correlations derived from the same dataset were compared using Steiger’s Z-test [30].

For docking comparisons, Welch’s *t*-test was used for two-group mean differences and one-sample *t*-test was used to assess whether site selectivity (ΔE) differed from zero within each group. Spearman correlation was used to analyze the relationship between docking-derived site selectivity and experimentally measured mixed inhibition. Bonferroni correction was applied to the seven primary docking metrics, and Benjamini–Hochberg false discovery rate control was used for exploratory analyses. Statistical analyses were performed using SPSS v12.0 (IBM Corp., Armonk, NY, USA) and GraphPad Prism v11.0 (GraphPad Software, Bosten, MA, USA), with *p* < 0.05 considered significant.

## 3. Results and Discussion

### 3.1. In Silico Screening and P1’ Frequency-Based Stratification

The results are presented in four linked levels: rule derivation in 24 stratified hydrolysates; independent evaluation in 16 composition-weighted commercial hydrolysates; biochemical examination of five candidates located at the prediction–experiment intersection; and external peptide-level benchmarking to define the length-dependent applicability domain. Docking and MD were used to interpret, rather than establish, the predictive rule.

Virtual hydrolysis of 84 commercially available food proteins under eight proteolytic conditions yielded 672 protein–enzyme combinations in the BIOPEP-UWM database. Of these, 591 (87.9%) produced at least one peptide fragment with a P1’-favorable residue, while the remaining 81 (12.1%) yielded exclusively non-favorable C-terminal residues, predominantly from trypsin digestions, whose strict cleavage specificity after Lys and Arg produces peptides terminating exclusively in basic residues that fall outside the P1’-favorable set {W, Y, F, P}.

Twenty-four protein–enzyme combinations were selected by tertile stratification (8 High, 8 Medium, 8 Low; Table 1; detailed subunit data in Table S3) to span the full range of predicted inhibitory potential while representing diverse protein sources (milk, beef, wheat) and enzyme classes. ACE-inhibitory activity was determined for all 24 hydrolysates at a fixed hydrolysate concentration of 0.5 mg mL^−1^. Percentage inhibition ranged from 7.8% (κ-casein/stem bromelain; Low tertile) to 96.3% (myoglobin/prolyl oligopeptidase; High tertile), while IC_50_ values ranged from 0.03 mg mL^−1^ (myoglobin/prolyl oligopeptidase) to 2.75 mg mL^−1^ (κ-casein/stem bromelain) (Table 1).

Final DH varied substantially among protein–protease combinations, ranging from 0.34% to 34.25%. However, high DH did not uniformly correspond to strong ACE inhibition. Thermolysin hydrolysates generally exhibited relatively high final DH but only modest inhibition, whereas several prolyl oligopeptidase hydrolysates showed strong inhibition despite low final DH. These observations indicate that peptide sequence composition, rather than the extent of hydrolysis alone, contributed substantially to the observed ACE-inhibitory activity.

P1’ frequency alone showed a moderate positive correlation with ACE inhibition (r = +0.550, *p* = 0.005; Table 1), confirming that the C-terminal residue identity provides a useful first-order predictor. However, the correlation with log_10_(1/IC_50_) was also positive (r = +0.503, *p* < 0.05), indicating that single-position screening has predictive value but remains weaker than the multi-position rules for potency ranking. This motivated the systematic comparison of multi-position rules described below.

### 3.2. Comparative Evaluation of Structure–Activity Rules

To improve predictive power beyond P1’ alone, the frequencies of Rules 1–5 were calculated for all 24 hydrolysates (Table 1; Table S5). Among the literature-derived rules, Rule 3 showed the strongest correlations (r = +0.691 with ACE inhibition; r = +0.704 with log_10_(1/IC_50_); both *p* < 0.001).

Rule 3 (P1’ in {W, Y, F, P} and P2’ in {L, I, V, K, R, H, W, Y, F}) showed strong correlations with ACE inhibition (r = +0.691, *p* < 0.001) and log_10_(1/IC_50_) (r = +0.704, *p* < 0.001). Rule 5, obtained by removing aromatic residues from the Rule 3 P2’ set, gave the highest correlations in this dataset.

**Figure 2:**
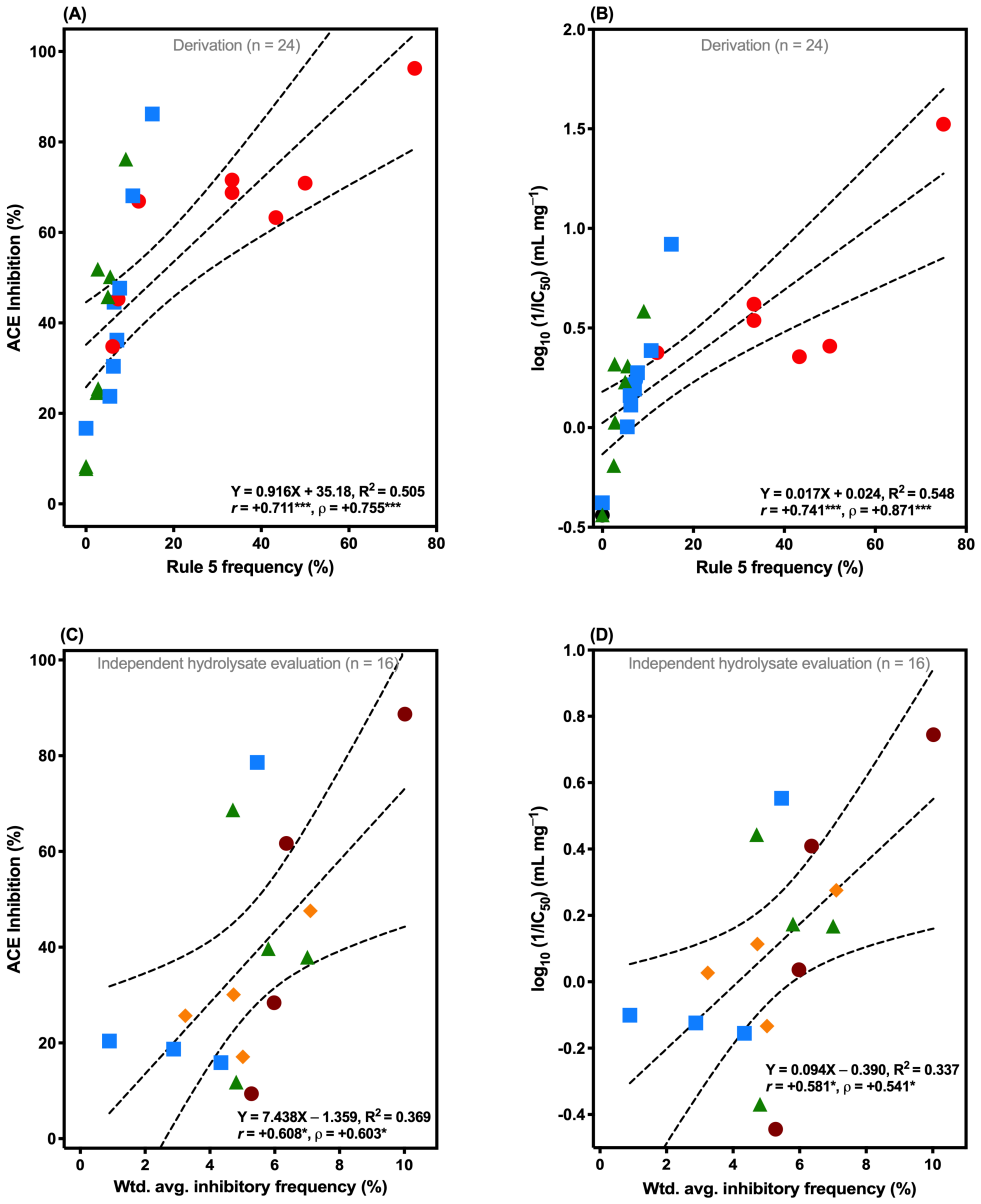
Associations between Rule 5 frequency and experimental ACE-inhibitory activity in protein–enzyme hydrolysates. Upper row: derivation dataset (n = 24); lower row: independent commercial-hydrolysate evaluation dataset (n = 16). (**A**) Rule 5 frequency versus ACE inhibition. (**B**) Rule 5 frequency versus log_10_(1/IC_50_). Data points in (**A**) and (**B**) are grouped by P1’-frequency tertile (red circles, High; blue squares, Medium; green triangles, Low). (**C**) Composition-weighted Rule 5 frequency versus ACE inhibition. (**D**) Composition-weighted Rule 5 frequency versus log_10_(1/IC_50_). Symbols in (**C**) and (**D**) indicate commercial protein source (red circles, milk protein; blue squares, beef protein; green triangles, wheat gluten; orange diamonds, wheat gliadin). The 16 commercial hydrolysates were not used to modify the Rule 5 residue definitions. Dashed lines represent linear regression with 95% confidence bands (shaded); Pearson r and Spearman ρ are shown in each panel. Rule 5: P1’ ∈ {W, Y, F, P} and P2’ ∈ {L, I, V, K, R, H}. Asterisks indicate significance levels (*, *p* < 0.05; ***, *p* < 0.001).

Against ACE inhibition, Rule 5 yielded r = +0.711 (*p* < 0.001) (Figure 2A), directionally higher than Rule 3 (r = +0.691) but not significantly different by Steiger’s Z-test (Z = 0.42, *p* = 0.674) [30]. Against log_10_(1/IC_50_), Rule 5 yielded the highest correlation (r = +0.741; Figure 2B), compared with Rule 1 (r = +0.588) and Rule 2 (r = +0.159). The complete rule comparison is provided in Table S5.

Thus, incorporating both P1’ and P2’ improved association with hydrolysate potency relative to simpler published criteria. Because Rule 5 was optimized within this dataset, these correlations support its selection for subsequent testing but should not be interpreted as independent validation.

### 3.3. Independent Evaluation with Commercial Food Protein Hydrolysates

To test practical applicability, composition-weighted Rule 5 frequencies were calculated for 16 hydrolysates prepared from four commercial protein sources (bovine milk, beef, wheat gluten, and wheat gliadin) using four enzymes each. Because the 16 combinations share four protein sources and four proteases, they are not fully independent. However, the design is factorial (4 proteins × 4 enzymes), and the correlation was assessed across the full 16-point matrix rather than within a single protein or enzyme group. Leave-one-protein-out and leave-one-protease-out sensitivity analyses showed that correlations remained significant in most subsets (r range: +0.414 to +0.697 for ACE inhibition; +0.368 to +0.702 for log_10_(1/IC_50_)); partial correlation controlling for protein source and protease identity yielded r = +0.518 (*p* = 0.040) for log_10_(1/IC_50_) (df = 8), although power is limited with n = 16 (detailed composition-weighting calculations in Tables S8–S11 and sensitivity analyses in Table S17). Weighting accounted for subunit composition so that in silico predictions better reflected the actual commercial materials used experimentally.

The composition-weighting procedure accounted for the heterogeneous subunit abundance of each commercial protein source, thereby making the virtual-hydrolysis metric more representative of the material used in the wet experiment (Table 2; Tables S8–S11).

Among these samples, milk–chymotrypsin showed the highest weighted Rule 5 frequency (10.02%) and the lowest IC_50_ (0.18 mg mL^−1^), consistent with chymotrypsin’s preference for aromatic C-terminal residues. Thermolysin hydrolysates also showed high frequencies owing to its broad hydrophobic-site specificity.

Across the 16 hydrolysates, weighted Rule 5 frequency correlated with ACE inhibition (r = 0.608, *p* < 0.05) and log_10_(1/IC_50_) (r = 0.581, *p* < 0.05; Figure 2D). Notably, Rule 5 was the only rule achieving statistical significance among Rules 1–5 in this independent set (Table S16). These samples were not used to modify the residue sets and therefore provide the principal independent hydrolysate-level evaluation of the rule. The correlations were moderate, as expected for complex hydrolysates in which peptide abundance, incomplete cleavage, matrix effects, and non-Rule 5 sequences also contribute to activity. Nevertheless, the consistent direction and magnitude across compositionally heterogeneous milk, beef, gluten, and gliadin preparations indicate that Rule 5 can assist pre-selection of promising protein–protease combinations rather than precisely predict the final IC_50_ of every product.

### 3.4. Experimental Examination of Peptides at the Prediction–Experiment Intersection

MALDI-TOF/TOF analysis produced 16 precursor-mass-supported sequence assignments for peptides of 5–13 residues and five low-mass candidate-associated features corresponding to Rule 5-compliant sequences predicted by the relevant virtual digestions (Table S12; Figure S2). Because matrix interference and limited mass accuracy in the low-mass region precluded unambiguous precursor-ion assignment, VF, GIF, LP/IP, and VP are described as putative candidates rather than definitive MALDI identifications. These candidates were advanced because they met the predefined intersection of virtual-digestion prediction, Rule 5 compliance, and a corresponding low-mass spectral feature; their ACE inhibition and kinetic properties were subsequently measured using independently synthesized peptides. The other detected peptides were retained as an exploratory structural comparison set; because they were generally longer (5–13 residues) than the Rule 5 candidates (2–3 residues), comparisons between the two groups necessarily reflect peptide length as well as motif compliance.

#### 3.4.1. ACE-Inhibitory Potency of the Five Prioritized Peptides

All five synthesized candidates inhibited ACE in a concentration-dependent manner, with IC_50_ values of 19.50–93.19 µM (Table 3). VF (19.50 ± 1.08 µM) and GIF (29.09 ± 1.26 µM), both terminating in phenylalanine, were more potent than the proline-terminated LP (53.13 ± 1.19 µM), IP (67.09 ± 1.62 µM), and VP (93.19 ± 6.51 µM). These results provide direct experimental support that candidates prioritized by the combined workflow are genuine ACE inhibitors; they do not imply that every Rule 5 sequence will be potent or that Rule 5 alone fully determines activity.

Comparison with published IC_50_ values revealed that LP, IP, and VP were 2.2-, 1.9-, and 4.5-fold more potent, respectively, than the corresponding literature values for the same dipeptides (LP: 117.80 µM [31]; IP: 130.00 µM [6]; VP: 420.00 µM [6]). VF (19.50 µM) was approximately 2.1-fold less potent than the value reported by Matsufuji et al. [32] (9.20 µM), likely reflecting differences in assay substrate (FAPGG vs. HHL) and reaction conditions. The incremental weakening of potency from LP to IP to VP mirrors the decreasing side-chain volume of P2’ (Leu > Ile > Val), consistent with the preference of the ACE S2’ subsite for branched aliphatic residues [7].

GIF has not, to our knowledge, previously been reported with a quantitative ACE IC_50_. Its predicted generation from wheat proteins, association with a low-mass MALDI feature, and independently measured low-micromolar activity illustrate the practical value of combining virtual digestion with conservative mass-spectrometric screening. More generally, the five peptides serve as experimentally tested exemplars of the screening framework, whereas the external database analysis below provides the broader assessment of generalizability.

**Table 3:** Putative Rule 5-compliant peptide candidates prioritized from wheat protein hydrolysates by protease-specific in silico digestion and corresponding low-mass MALDI-TOF/TOF features, followed by activity testing of synthesized peptides.

Peptide	P1’	P2’	Calc. M (Da)	Obs. *m*/*z* ^a^	Hydrolysate Source	IC_50_ (µM)		Reference
						This Study	Literature	
VF	F	V	264.15	265.994	Glu-Pep (pH 1.3)	19.50 ± 1.08	9.20	[32]
GIF	F	I	335.18	335.365/335.377	Glu-Pep (pH 1.3)/ Glu-Pep (pH > 2.0)	29.09 ± 1.26	ND	—
LP	P	L	228.15	228.074 ^b^	Glu-Bro	53.13 ± 1.19	117.80	[32]
IP	P	I	228.15	228.074 ^b^	Glu-Bro	67.09 ± 1.62	130.00	[6]
VP	P	V	214.13	213.124	Glu-Bro	93.19 ± 6.51	420.00	[6]

^a^ Candidate-associated low-mass feature observed by MALDI-TOF/TOF. Owing to matrix interference and limited mass accuracy below *m*/*z* 400, these values were not treated as accurate [M+H]^+^ precursor matches and do not constitute definitive sequence identification. ^b^ LP (Leu-Pro) and IP (Ile-Pro) share the same molecular formula (C_11_H_20_N_2_O_3_; M_r_ = 228.29 g mol^−1^) and were associated with the same unresolved low-mass feature at *m*/*z* 228.074. LP and IP were retained as alternative isomeric candidates based on in silico digestion predictions. Because leucine and isoleucine are isobaric and indistinguishable by MALDI-TOF mass spectrometry alone, neither the individual identity nor the simultaneous presence of both peptides can be confirmed without chromatographic separation or MS/MS fragmentation capable of resolving Leu/Ile (e.g., ECD or UVPD). The reported IC_50_ values for LP and IP were measured using independently synthesized peptides and therefore remain valid regardless of which isomer was present in the hydrolysate. ACE, angiotensin-converting enzyme; Rule 5: P1’ ∈ {W, Y, F, P} and P2’ ∈ {L, I, V, K, R, H}. Calc. M_r_ values are monoisotopic molecular weights. P1’, first C-terminal residue of inhibitory peptide (ACE S1’ subsite); P2’, second C-terminal residue (ACE S2’ subsite). Hydrolysate abbreviations: Glu-Pep, wheat gluten/pepsin; Glu-Bro, wheat gluten/bromelain. ND, not determined (no literature IC_50_ value available for ACE inhibition); —, no reference available. Literature IC_50_: VF (Val-Phe) from Matsufuji et al. [32]; LP (Leu-Pro) from Wei et al. [31]; IP (Ile-Pro) and VP (Val-Pro) from Cheung et al. [6]. For GIF (Gly-Ile-Phe), no quantitative ACE-inhibitory IC_50_ value was identified in the literature searched.

#### 3.4.2. Enzyme Kinetic Analysis and Inhibition Mode

**Figure 3:**
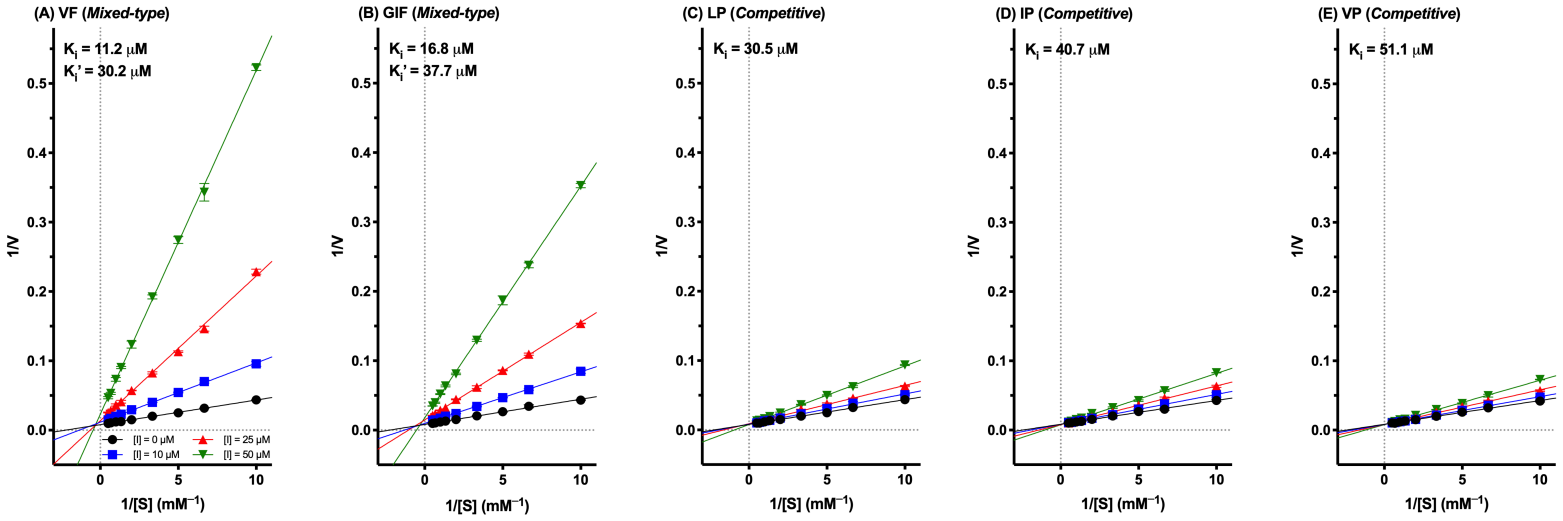
Lineweaver–Burk double-reciprocal plots for ACE inhibition by five Rule 5-compliant peptides. (**A**) VF (Val-Phe), mixed-type inhibition, K_i_ = 11.2 µM. (**B**) GIF (Gly-Ile-Phe), mixed-type inhibition, K_i_ = 16.8 µM. (**C**) LP (Leu-Pro), competitive inhibition, K_i_ = 30.5 µM. (**D**) IP (Ile-Pro), competitive inhibition, K_i_ = 40.7 µM. (**E**) VP (Val-Pro), competitive inhibition, K_i_ = 51.1 µM. FAPGG substrate concentrations: 0.1–4.0 mM; inhibitor concentrations: 0, 10, 25, and 50 µM. Initial velocity (V) expressed as nmol/min/mg protein. Mixed-type inhibition (**A**,**B**): lines intersect in the second quadrant. Competitive inhibition (**C**–**E**): lines converge on the y-axis (constant 1/V_max_). Data represent mean ± SD (n = 3).

Lineweaver–Burk double-reciprocal analysis revealed two distinct inhibition patterns among the five Rule 5 peptides (Figure 3). VF and GIF exhibited mixed-type inhibition: the double-reciprocal lines intersected in the second quadrant, indicating simultaneous increases in apparent K_m_ and decreases in V_max_. Secondary replots of apparent K_m_ and apparent 1/V_max_ versus inhibitor concentration yielded K_i_ = 11.2 ± 0.9 µM and K_i_’ = 30.2 ± 2.1 µM for VF, and K_i_ = 16.8 ± 1.4 µM and K_i_’ = 37.7 ± 2.8 µM for GIF. The ratios K_i_’/K_i_ of 2.7 (VF) and 2.2 (GIF) indicate that both peptides bind preferentially to free ACE but retain substantial affinity for the enzyme–substrate complex. In contrast, LP, IP, and VP displayed competitive inhibition, with Lineweaver–Burk lines converging on the y-axis (constant 1/V_max_) and inhibition constants K_i_ = 30.5 ± 2.6, 40.7 ± 3.2, and 51.1 ± 4.5 µM, respectively (Table S14).

Within this limited five-peptide panel, inhibition mode was associated with the C-terminal residue: VF and GIF (P1’ = F) showed mixed-type inhibition, whereas LP, IP, and VP (P1’ = P) showed competitive inhibition. This internally consistent pattern suggests that C-terminal identity may influence binding behavior, but the small and structurally restricted panel does not support a universal mechanistic rule. Confirmation would require a larger, length-matched set in which P1’ identity is varied independently of the remaining sequence.

#### 3.4.3. Docking-Based Interpretation of Peptide Length and Site Accessibility

**Figure 4:**
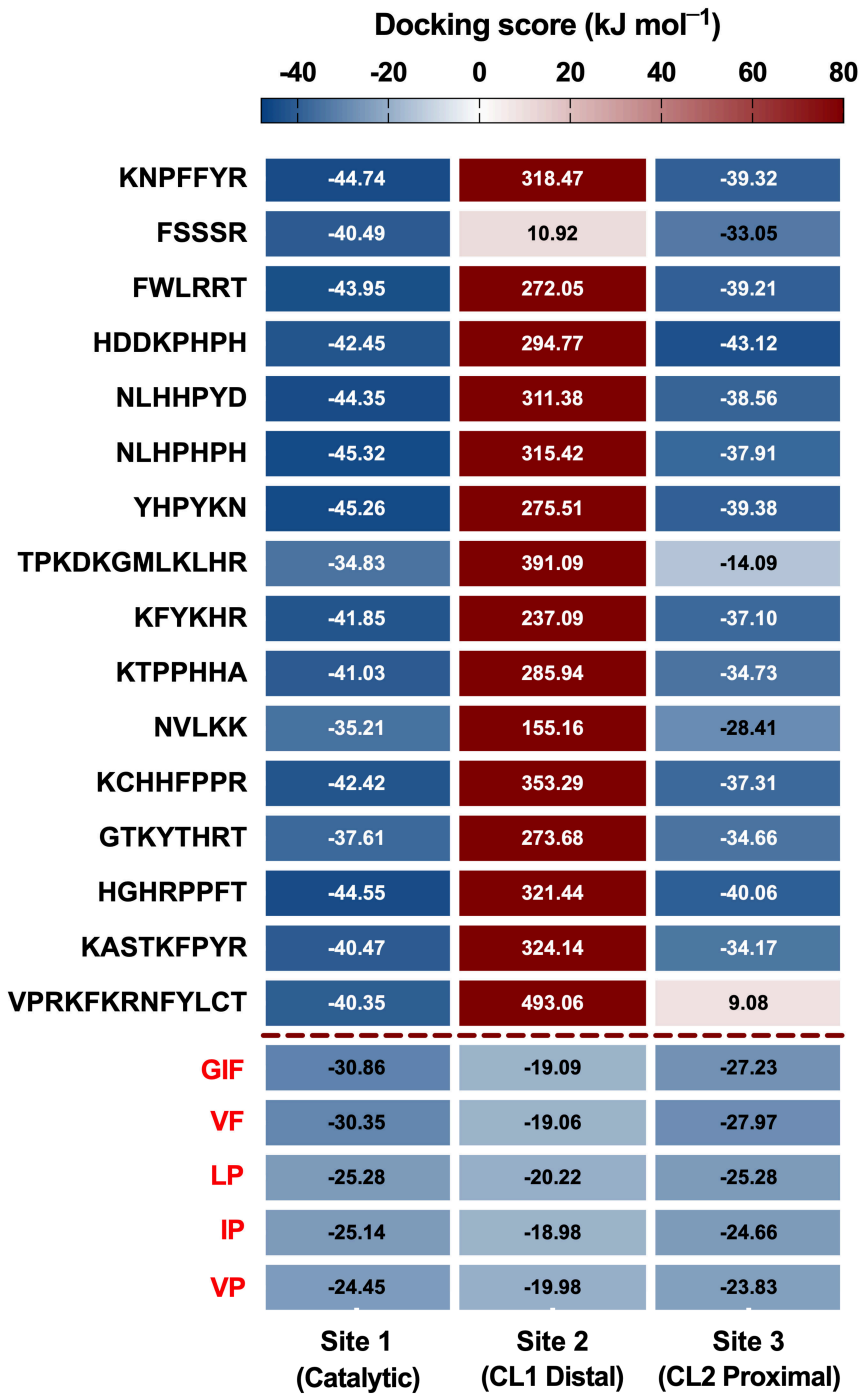
Docking-score heatmap for 16 precursor-mass-supported peptide assignments and five putative short-peptide candidates prioritized by low-mass MALDI-TOF/TOF features and protease-specific virtual digestion, docked against three ACE regions. Site 1, catalytic Zn^2+^-binding site; Site 2, chloride-1 (CL1) distal pocket; Site 3, chloride-2 (CL2) proximal pocket. The five short Rule 5 candidates (GIF, VF, LP, IP, and VP; 2–3 residues) are marked with stars and displayed separately from the 16 longer comparison peptides (5–13 residues). Positive Site 2 scores for the longer peptides are consistent with steric exclusion from the narrow CL1-associated channel, whereas the five short candidates yielded favorable negative scores. Because Rule 5 status and peptide length were confounded in this set, the comparison is interpreted as exploratory. Docking was performed with AutoDock Vina against human testicular ACE (PDB: 1O86).

All 21 detected peptides were docked to the catalytic site (Site 1), the CL1 distal pocket (Site 2), and the CL2 proximal pocket (Site 3). These calculations were used exploratorily to interpret the observed length dependence. Because the five Rule 5 candidates were di- or tripeptides whereas the comparison peptides contained 5–13 residues, the analysis cannot isolate motif compliance from peptide length (Figure 4).

**Figure 5:**
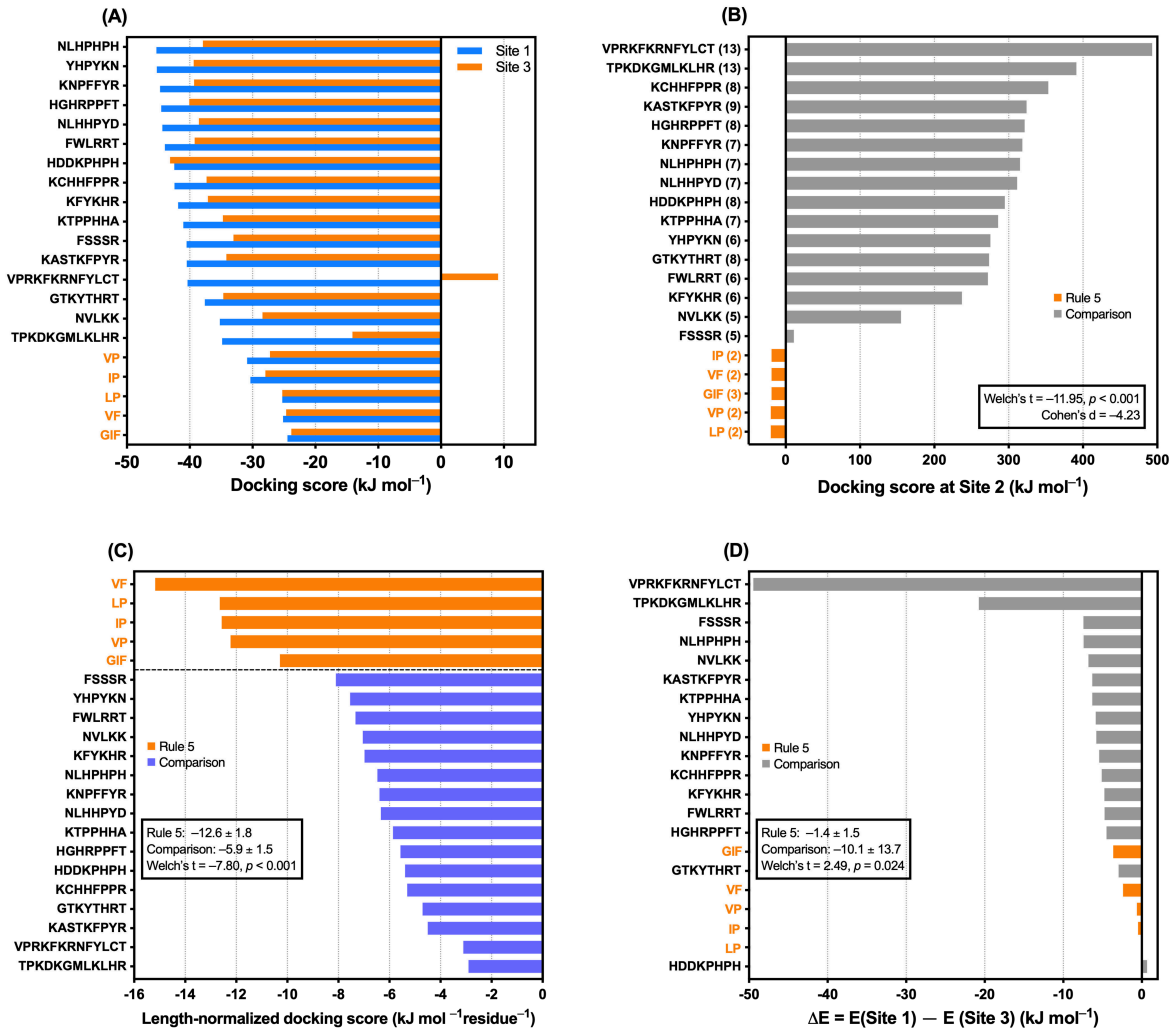
Exploratory docking comparison between the five short Rule 5 candidates and 16 longer comparison peptides across three ACE regions. (**A**) Docking scores at Site 1 and Site 3. (**B**) Docking scores at the CL1 distal pocket (Site 2); Rule 5 and Comparison are the abbreviated group labels used in the figure. (**C**) Length-normalized docking score, calculated as the most favorable docking score divided by peptide length. (**D**) Exploratory site-selectivity index, ΔE = E(Site 1) − E(Site 3); values closer to zero indicate more similar calculated scores at the two sites. Rule 5 peptides contained 2–3 residues, whereas comparison peptides contained 5–13 residues; therefore, group differences also reflect peptide length. The nominal between-group difference in panel D (*p* = 0.024) did not remain significant after Bonferroni correction. Docking was performed with AutoDock Vina against human testicular ACE (PDB: 1O86; exhaustiveness = 128; 20 binding modes per run).

At Site 1 (catalytic), all 21 peptides yielded favorable docking scores. The longer comparison peptides showed more negative values (−35.1 to −45.2 kJ mol^−1^) than the five short Rule 5 candidates (−24.3 to −31.0 kJ mol^−1^; Welch’s t = 9.14, *p* < 0.001; Figure 5A), consistent with the greater number of potential contacts available to longer sequences. At Site 3 (CL2 proximal), the two groups showed broadly overlapping score distributions, and the difference did not survive Bonferroni correction (*p* = 0.150).

All 16 longer comparison peptides showed unfavorable Site 2 docking scores (+10.9 to +493.1 kJ mol^−1^), whereas the five short candidates showed favorable values (−12.6 to −23.4 kJ mol^−1^). The narrow CL1-associated channel therefore appears geometrically compatible with di- and tripeptides but not with the longer sequences examined here. This result offers a plausible structural explanation for a short-peptide applicability domain; it should not be interpreted as independent validation of Rule 5 because length and compliance were confounded in the available set.

#### 3.4.4. Length-Normalized Docking Score

To reduce the inherent energetic advantage of longer peptides, the most favorable docking score was divided by peptide length. The five short candidates showed more favorable length-normalized docking scores than the longer comparison peptides (−12.6 ± 1.8 vs. −5.9 ± 1.5 kJ mol^−1^ residue^−1^; *p* < 0.001; Figure 5C). However, the strong association between peptide length and this normalized metric indicates that the result primarily describes efficient packing by short peptides and should not be presented as proof of motif-specific complementarity.

#### 3.4.5. Exploratory Relationship Between Docking Site Preference and Inhibition Kinetics

Site selectivity, defined as ΔE = E(Site 1) − E(Site 3), was closer to zero for the five short candidates than for the longer comparison peptides (Figure 5D), indicating similar calculated accessibility to the two modeled sites. The mean ΔE of the Rule 5 group did not differ significantly from zero (*p* = 0.105), and the between-group comparison (*p* = 0.024) did not meet the Bonferroni-adjusted threshold used for the primary docking metrics. Accordingly, the dual-site interpretation remains exploratory.

VF and GIF combined the strongest multi-site docking scores with mixed-type kinetics, whereas LP, IP, and VP showed competitive kinetics. The rank association between ΔE and the binary inhibition-mode coding (ρ = 0.900, *p* = 0.037) is intriguing but is based on only five observations and on a post hoc binary outcome. It therefore provides hypothesis-generating consistency, not mutual validation of the docking and kinetic results.

Taken together, the docking results suggest that peptide length strongly affects access to the modeled ACE pockets, while the observed difference between F- and P-terminated peptides warrants targeted evaluation with a larger, length-matched series.

#### 3.4.6. Interpretation of the Convergent Evidence

Several docking metrics differed between the short Rule 5 candidates and the longer comparison peptides (Figure 4 and Figure 5), particularly the Site 2 docking score and the length-normalized docking score. These findings are structurally coherent with size-dependent pocket accessibility, but their interpretation is limited by the absence of length-matched non-compliant controls.

The strongest evidence supporting the screening framework therefore comes from three levels: association with activity in the 24-hydrolysate derivation set, retained predictive utility in the independent 16-hydrolysate evaluation set, and direct ACE inhibition by all five candidates selected at the prediction–experiment intersection. Docking provides a plausible structural context for the length dependence but is not treated as proof of Rule 5 specificity.

### 3.5. External Benchmarking and Applicability Domain of Rule 5

External benchmarking was performed using 1429 unique ACE-inhibitory peptides with quantitative IC50 values compiled from AHTPDB, BIOPEP-UWM, and MBPDB (Dataset S1). When all lengths were pooled, compliant and non-compliant peptides did not differ significantly (median IC_50_, 44.3 vs. 59.9 µM; *p* > 0.05). This negative pooled result was important: it showed that Rule 5 should not be used as a universal classifier without considering peptide length. To assess potential circularity, 155 peptides overlapping with the Wu et al. [7] training set were identified and excluded; the 2–3 aa enrichment remained significant after exclusion (z = −3.006, *p* = 0.003), whereas the 2–4 aa window (Figure S3) lost significance (Table S13).

**Figure 6:**
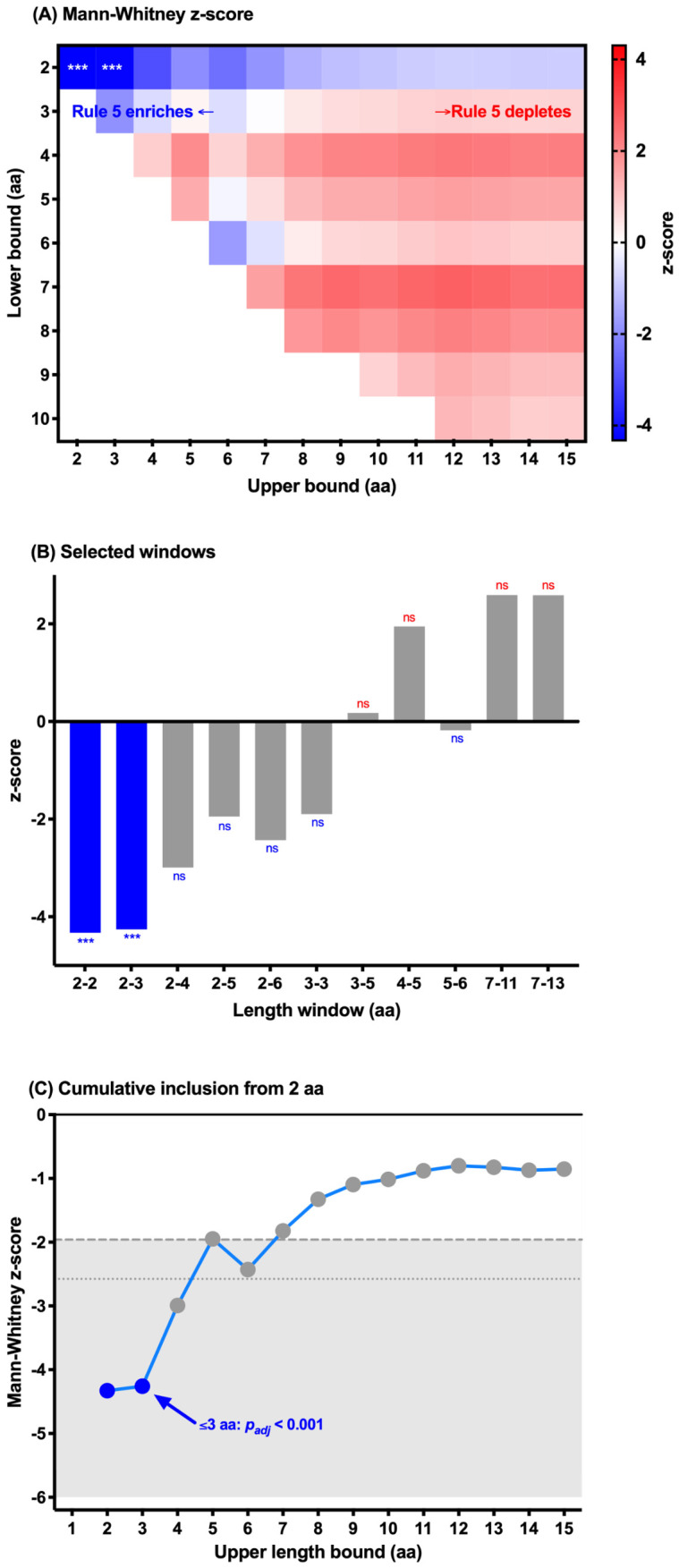
Length-stratified external benchmarking of Rule 5 against 1429 unique ACE-inhibitory peptides compiled from AHTPDB, BIOPEP-UWM, and MBPDB. (**A**) Heatmap of Mann–Whitney z-scores across all length windows (lower bound × upper bound); blue indicates that Rule 5-compliant peptides are significantly more potent, red indicates reversed enrichment. Asterisks denote Benjamini–Hochberg-corrected significance across 87 windows tested (*** *p*_adj_ < 0.001). (**B**) z-scores for selected length windows; *** *p*_adj_ < 0.001, ns = not significant after BH-FDR correction. (**C**) Cumulative inclusion curve with lower bound fixed at 2 aa; the applicability boundary of Rule 5 falls at ≤3 amino acids (*p*_adj_ < 0.001). Significance thresholds: dashed line, z = −1.96 (uncorrected *p* = 0.05); dotted line, z = −2.576 (uncorrected *p* = 0.01).

Among di- and tripeptides (2–3 aa, n = 470), Rule 5 enrichment was highly significant: compliant peptides had a median IC_50_ of 28.0 µM versus 79.0 µM for non-compliant peptides (Mann–Whitney z = −4.259, *p*_adj_ < 0.001, Benjamini–Hochberg-corrected across 87 length windows; Table S15; Figure 6A,B). Extending the window to 2–4 aa (n = 631; z = −2.991) did not survive BH-FDR correction (*p*_adj_ = 0.081), and enrichment attenuated further by 2–5 aa (z = −1.946, *p* > 0.05). The cumulative inclusion curve (Figure 6C) identified ≤3 amino acids as the applicability boundary of Rule 5 (*p*_adj_ < 0.001), beyond which the inclusion of longer peptides progressively diluted the enrichment signal.

For peptides of ≥7 residues, the direction of enrichment reversed, with compliant sequences showing weaker activity in several length windows. This reversal confirms that C-terminal motif information alone becomes insufficient—and may even be misleading—when additional residues dominate conformation and binding. The docking analysis offers one possible structural explanation through size-dependent pocket accessibility, but the database result itself is the primary evidence defining the applicability boundary.

It should be noted that Rule 5 frequency, as defined by Equation (2), represents the proportion of theoretically predicted peptide fragments matching the C-terminal motif and does not account for actual peptide abundance in the hydrolysate. Peptide abundance in practice is governed by hydrolysis efficiency, substrate folding and accessibility, enzyme–product inhibition, and peptide stability during processing. Rule 5 therefore predicts structural potential—the likelihood that a given protein–protease combination generates sequences with the preferred C-terminal motif—rather than the absolute quantity of active peptides. Quantitative peptidomics would be required to correlate motif frequency with actual peptide concentrations.

**Figure 7:**
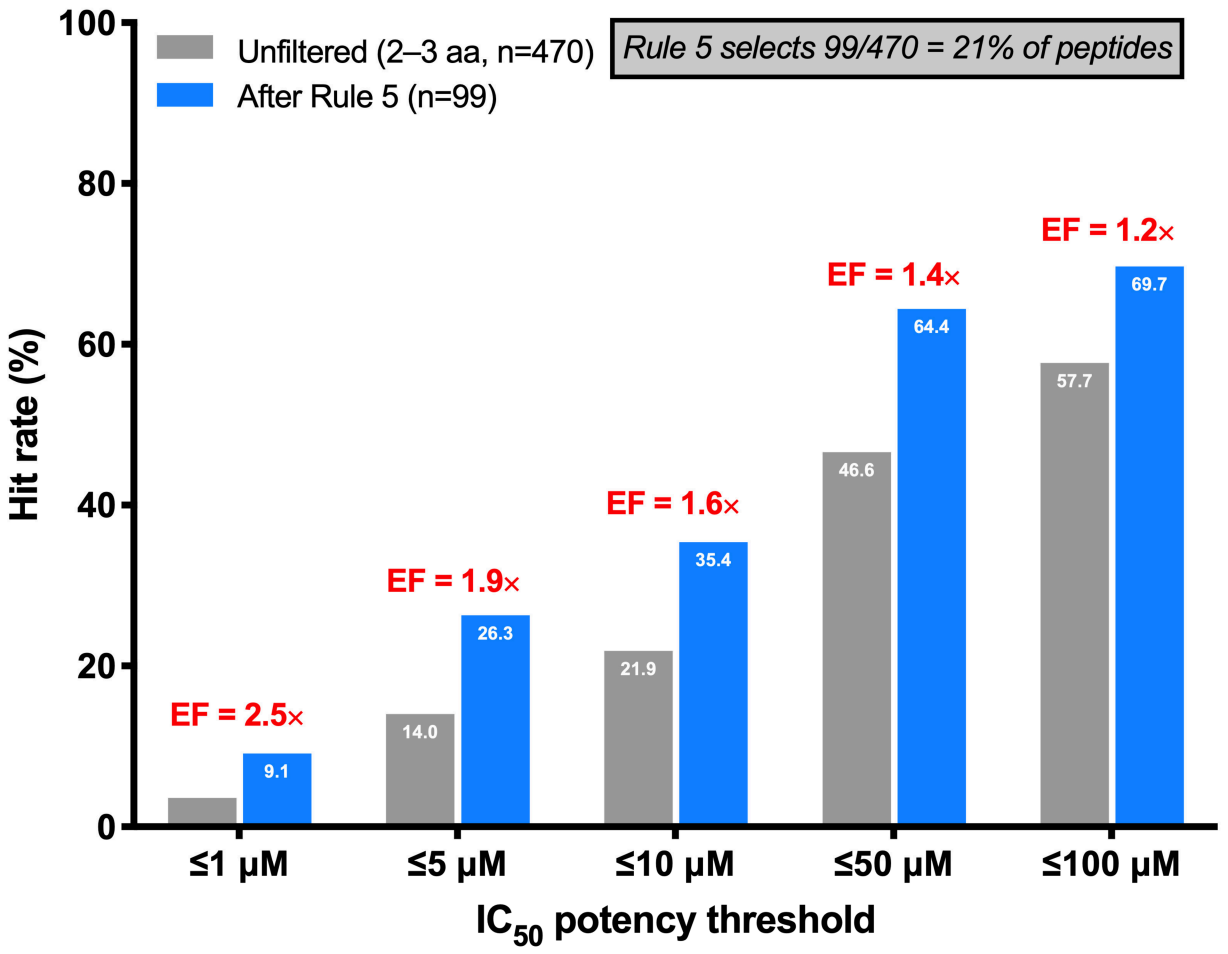
Screening enrichment of Rule 5 among 2–3 aa ACE-inhibitory peptides from external databases (n = 470). Gray bars: baseline proportion without screening; blue bars: proportion after Rule 5 filtering. EF, enrichment factor. Rule 5 selects 99/470 (21%) of peptides while enriching for potent inhibitors at all IC_50_ thresholds.

Within the 2–3-residue domain, Rule 5 selected 99 of 470 peptides (21.1%) and increased the proportion of sub-micromolar inhibitors from 3.6% in the unscreened set to 9.1% after filtering (EF = 2.5) (Figure 7). At IC_50_ ≤ 10 µM, the proportion increased from 21.9% to 35.4% (EF = 1.6). Rule 5 should therefore be interpreted as an enrichment tool that reduces the candidate pool while increasing the proportion of potent inhibitors, not as a binary guarantee of potency.

### 3.6. Molecular Dynamics Assessment of Contact Persistence

**Figure 8:**
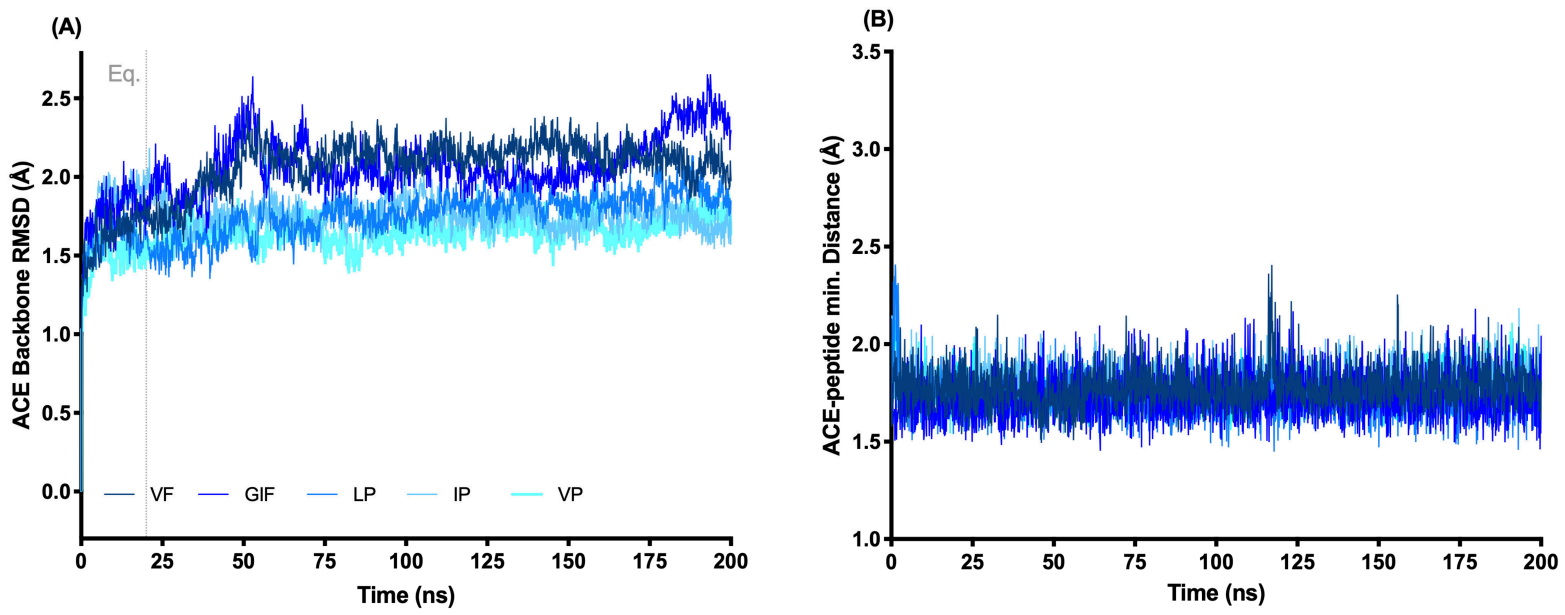
ACE backbone stability and peptide–ACE contact persistence during 200 ns MD simulations. (**A**) ACE backbone RMSD (chain A, residues 1–566) over time; the dashed vertical line marks the 20 ns equilibration cutoff. (**B**) Minimum heavy-atom distance between ACE and each peptide. All five systems maintained peptide–ACE contact throughout the simulated trajectories, with no complete dissociation events observed. Minimum distance was used to assess contact persistence and does not establish binding-pose retention, residence time, or thermodynamic stability.

All five docked peptide–ACE complexes were subjected to 200 ns MD simulations (Figure 8). ACE backbone RMSD stabilized within the first 20 ns and remained between 1.67 ± 0.09 and 2.09 ± 0.17 Å over 20–200 ns, indicating preservation of the overall protein fold under the simulated conditions.

The minimum heavy-atom distance between ACE and each peptide remained 2.66–2.71 Å (mean ± SD across five peptides: 2.70 ± 0.02 Å), consistent with persistent van der Waals contact throughout the 200 ns trajectories. These results support persistent peptide–protein contact from the selected starting poses. Because minimum distance does not quantify binding free energy, pose uniqueness, or residence time, the simulations are not interpreted as confirmation of thermodynamic stability or of the experimentally observed inhibition mechanism.

### 3.7. Study Limitations and Future Directions

Several limitations should be considered. First, BIOPEP-UWM assumes complete cleavage of accessible sequence sites, whereas experimental hydrolysis is influenced by folding, aggregation, protease accessibility, and product inhibition. Second, Rule 5 was refined within a 24-hydrolysate derivation set; although the 16 commercial hydrolysates and the 1429-peptide database provide independent evaluation, further testing in additional protein systems remains desirable. Third, the five short-peptide candidates were supported only by low-mass MALDI features and virtual-digestion predictions; matrix interference and limited mass accuracy prevented definitive precursor-ion assignment, and LP/IP could not be resolved isomerically. Accordingly, their sequence-specific biochemical results derive from independently synthesized standards. The remaining MALDI-based assignments may be ambiguous for isomeric peptides, particularly LP and IP, and quantitative peptidomics would be required to relate motif abundance directly to hydrolysate potency. Fourth, the docking comparison confounded motif compliance with peptide length because all compliant candidates were short and all comparison peptides were longer. Finally, MD simulations began from docked poses and monitored contact persistence rather than binding free energy.

Rule 5 is therefore restricted to first-pass prioritization of peptides containing 2–3 residues. Notably, di- and tripeptides within this applicability domain are efficiently absorbed via PepT1-mediated intestinal transport, which may support their bioavailability potential, although gastrointestinal stability and actual transport rates were not evaluated in this study. Longer ACE-inhibitory peptides require models that incorporate full-sequence physicochemical descriptors, conformational sampling, or machine learning. For functional-food translation, future studies should additionally quantify peptide abundance in real hydrolysates and examine gastrointestinal stability, intestinal transport, food-matrix effects, and antihypertensive efficacy in appropriate biological models.

## 4. Conclusions

This study developed an interpretable C-terminal rule for prioritizing short ACE-inhibitory peptides from food protein hydrolysates. Rule 5 (P1’ ∈ {W, Y, F, P}; P2’ ∈ {L, I, V, K, R, H}) showed the strongest internal performance in a 24-hydrolysate derivation set and retained predictive utility in an independent set of 16 composition-weighted commercial hydrolysates. Five peptides selected at the intersection of virtual cleavage prediction, Rule 5 compliance, and candidate-associated low-mass MALDI features—VF, GIF, LP, IP, and VP—were all confirmed to inhibit ACE, demonstrating that the workflow can prioritize sequences that show ACE inhibition when synthesized and tested independently.

Benchmarking against 1429 quantitative ACE-inhibitory peptides established that the rule is length-dependent rather than universal. It significantly enriched potent inhibitors among di- and tripeptides (2–3 residues; BH-FDR *p*_adj_ < 0.001), with a 2.5-fold enrichment at the sub-micromolar threshold, but lost predictive value as longer peptides were included. Docking and MD results were consistent with efficient accommodation and persistent ACE contact by the short candidates, while also emphasizing that peptide length strongly influenced the structural comparisons.

Accordingly, Rule 5 should be used as a transparent first-pass enrichment filter. It can help food scientists pre-select protein–protease combinations enriched in short ACE-inhibitory peptides, thereby reducing the cost and experimental burden of empirical hydrolysate screening before fractionation, peptide synthesis, and kinetic verification. However, it does not replace quantitative peptidomics, biochemical confirmation, gastrointestinal stability testing, in vivo efficacy evaluation, or more complex modeling of longer sequences. Translation to functional-food applications would additionally require demonstration of peptide stability during food processing and gastrointestinal digestion, intestinal absorption, and antihypertensive efficacy in appropriate animal or clinical models.

## Figures and Tables

**Table 1:** Protein–enzyme combinations selected for construction of the 24-hydrolysate rule-development set, stratified by P1’-preferred-residue frequency tertile (*n* = 24).

#	Tertile ^a^	Protein	BIOPEP ID ^b^	Enzyme	No. of Peptides ^c^	P1’ Freq. (%) ^d^	ACE Inh. (%)	IC_50_ (mg mL^−1^)	End Point (h) ^e^	Final DH (%)
1	High	Lactoferrin	1212	Prolyl oligopeptidase	30	96.67	63.3 ± 0.4	0.44 ± 0.00	0.67	8.34 ± 0.31
2	High	κ-Casein	1117	Prolyl oligopeptidase	20	95.00	70.9 ± 0.3	0.39 ± 0.01	0.33	5.67 ± 0.13
3	High	β-Lactoglobulin A	1851	Prolyl oligopeptidase	9	88.89	71.6 ± 0.3	0.24 ± 0.01	0.33	1.32 ± 0.03
4	High	Myoglobin	1272	Prolyl oligopeptidase	4	75.00	96.3 ± 0.0	0.03 ± 0.00	0.33	0.47 ± 0.04
5	High	α-Lactalbumin	1854	Prolyl oligopeptidase	3	66.67	68.8 ± 0.3	0.29 ± 0.00	0.33	0.34 ± 0.03
6	High	Gluten	1329	Pepsin (pH 1.3)	41	53.66	45.3 ± 0.5	0.58 ± 0.00	3.0	8.71 ± 0.26
7	High	Gliadin	1319	Papain	82	43.90	34.8 ± 0.7	0.69 ± 0.00	3.0	5.02 ± 0.28
8	High	Lactoferrin	1212	Chymotrypsin A	125	37.60	66.9 ± 0.3	0.42 ± 0.01	4.0	8.62 ± 0.73
9	Medium	α-Lactalbumin	1854	Chymotrypsin A	28	32.14	68.1 ± 0.3	0.41 ± 0.00	4.0	5.60 ± 0.38
10	Medium	κ-Casein	1117	Pepsin (pH 1.3)	16	31.25	30.4 ± 0.7	0.77 ± 0.01	3.0	7.99 ± 0.24
11	Medium	Gluten	1329	Pepsin (pH > 2)	85	30.59	36.2 ± 0.6	0.64 ± 0.01	2.0	3.20 ± 0.31
12	Medium	Gliadin	1319	Thermolysin	55	29.09	23.8 ± 0.8	0.99 ± 0.01	1.33	34.25 ± 1.89
13	Medium	Lactoferrin	1212	Pepsin (pH 1.3)	78	25.64	44.6 ± 0.6	0.61 ± 0.01	4.0	4.19 ± 0.02
14	Medium	Gluten	1329	Stem bromelain	65	24.62	16.7 ± 0.8	2.38 ± 0.04	3.0	4.44 ± 0.30
15	Medium	β-Lactoglobulin A	1851	Chymotrypsin A	33	24.24	86.2 ± 0.1	0.12 ± 0.00	4.0	16.29 ± 0.24
16	Medium	Myoglobin	1272	Chymotrypsin A	39	23.08	47.7 ± 0.5	0.53 ± 0.00	3.0	3.76 ± 0.26
17	Low	Myoglobin	1272	Papain	40	12.50	45.8 ± 0.5	0.59 ± 0.02	1.0	0.81 ± 0.03
18	Low	Lactoferrin	1212	Papain	185	11.89	51.9 ± 0.5	0.48 ± 0.01	3.0	11.88 ± 0.67
19	Low	β-Lactoglobulin A	1851	Pepsin (pH > 2)	36	11.11	50.2 ± 0.5	0.49 ± 0.02	3.0	3.54 ± 0.35
20	Low	α-Lactalbumin	1854	Thermolysin	30	10.00	8.3 ± 0.9	2.74 ± 0.06	1.0	21.66 ± 0.56
21	Low	Gluten	1329	Trypsin	11	9.09	76.2 ± 0.2	0.26 ± 0.00	3.0	3.46 ± 0.26
22	Low	Lactoferrin	1212	Thermolysin	144	8.33	25.5 ± 0.7	0.94 ± 0.02	1.0	24.28 ± 2.13
23	Low	κ-Casein	1117	Stem bromelain	37	5.41	7.8 ± 0.9	2.75 ± 0.02	2.0	2.55 ± 0.16
24	Low	Myoglobin	1272	Thermolysin	40	2.50	24.6 ± 0.8	1.55 ± 0.02	1.0	23.57 ± 1.64
Pearson’s r (P1’ frequency vs ACE Inh.%)					+0.550 **					
Pearson’s r (P1’ frequency vs log_10_(1/IC_50_))					+0.503 *					

^a^ Protein–enzyme combinations were stratified into tertiles (High, Medium, Low) based on P1’ frequency among non-zero combinations (n = 591 out of 672 total from 84 commercial proteins × 8 enzymes in BIOPEP-UWM); tertile cut-points: Low ≤ 13.01%, Medium 13.08–32.58%, High ≥ 32.84%. ^b^ BIOPEP ID: accession number in the BIOPEP-UWM peptide database. ^c^ Number of peptide fragments (≥2 amino acids; free amino acids excluded) generated by in silico hydrolysis. ^d^ P1’ inhibitory frequency (%) = (fragments with P1’ residue ∈ {W, Y, F, P}/total fragments) × 100%; P1’, C-terminal residue position per Schechter & Berger [12]. * *p* < 0.05; ** *p* < 0.01 (Pearson’s product-moment correlation, two-tailed, n = 24). ^e^ Endpoint indicates the hydrolysis time at which DH reached a plateau according to the criterion described in Section 2.4. Final DH values are expressed as mean ± SD from three independently prepared hydrolysis batches (n = 3).

**Table 2:** Summary of Rule 5-compliant peptide frequency and composition-weighted Rule 5-compliant peptide frequency for commercial food protein hydrolysates (Rule 5: P1’ ∈ {W, Y, F, P} and P2’ ∈ {L, I, V, K, R, H}). This set was not used to modify Rule 5 residue definitions.

Protein Source	Enzyme	No. of Subunits ^a^	Inh. Freq. Range (%) ^b^	Wtd. Avg. Inh. Freq. (%) ^c^	ACE Inh. (%) ^d^	IC_50_ (mg mL^−1^) ^d^
Milk protein	Thermolysin	8	0.00–14.63	6.36	61.7 ± 0.5	0.39 ± 0.01
	Pepsin (pH > 2)	8	1.85–15.56	5.98	28.4 ± 1.4	0.92 ± 0.03
	Papain	8	2.13–12.73	5.28	9.4 ± 0.6	2.78 ± 0.05
	Chymotrypsin	8	7.50–18.60	10.02	88.7 ± 0.2	0.18 ± 0.00
Beef protein	Thermolysin	9	0.00–4.39	0.90	20.4 ± 1.4	1.26 ± 0.03
	Pepsin (pH > 2)	9	0.00–11.76	4.34	15.9 ± 1.2	1.43 ± 0.02
	Papain	9	0.00–6.38	2.88	18.7 ± 1.3	1.33 ± 0.01
	Chymotrypsin	9	0.00–12.12	5.46	78.6 ± 0.2	0.28 ± 0.00
Wheat gluten	Thermolysin	5	4.08–9.43	7.01	37.9 ± 0.7	0.68 ± 0.01
	Pepsin (pH > 2)	5	2.25–8.33	4.71	68.7 ± 0.5	0.36 ± 0.00
	Papain	5	3.80–6.10	4.81	11.8 ± 1.0	2.34 ± 0.04
	Chymotrypsin	5	2.17–8.70	5.80	39.7 ± 0.8	0.67 ± 0.01
Wheat gliadin	Thermolysin	3	4.08–9.43	7.10	47.6 ± 0.3	0.53 ± 0.01
	Pepsin (pH > 2)	3	2.25–4.41	3.24	25.7 ±1.6	0.94 ± 0.01
	Papain	3	3.80–6.10	5.02	17.1 ± 0.5	1.36 ± 0.01
	Chymotrypsin	3	2.17–6.38	4.73	30.1 ± 1.3	0.77 ± 0.01
Pearson’s r ^e^					+0.608 *	+0.581 **

^a^ Number of protein subunits included in the BIOPEP-UWM in silico hydrolysis for each commercial protein source. ^b^ Rule 5 freq., Rule 5-compliant peptide frequency (%) = (Pred. inh. frag./Total frag.) × 100%. Pred. inh. frag., predicted inhibitory fragments: peptides (≥2 amino acids) whose C-terminal residue (P1’) ∈ {W, Y, F, P} and penultimate residue (P2’) ∈ {L, I, V, K, R, H}. ^c^ Wtd. Rule 5 freq., composition-weighted Rule 5-compliant peptide frequency (%) = Σ [(% of protein subunit/Σ % of protein subunits) × Rule 5-compliant peptide frequency]. ^d^ ACE inhibition at 0.5 mg mL^−1^ and IC_50_ values determined experimentally using three independently prepared hydrolysis batches (mean ± SD for ACE inhibition, n = 3). ^e^ Pearson’s product-moment correlation coefficients between composition-weighted Rule 5-compliant peptide frequency and (i) ACE inhibition (%) or (ii) log_10_(1/IC_50_) (n = 16 protein–enzyme combinations). Significance: * *p* < 0.05; ** *p* < 0.01 (two-tailed).

## Data Availability

The dataset generated in this study is publicly available on Zenodo at https://doi.org/10.5281/zenodo.21708604. Additional materials are available from the corresponding authors upon reasonable request.

## Supplementary Materials

The following supporting information can be downloaded at: https://www.mdpi.com/article/10.3390/foods15152764/s1. References [33,34,35,36] are cited in Supplementary Materials.
