# Supplementary material for: A Length-Aware C-Terminal Rule for Prioritizing Short ACE-Inhibitory Peptides from Food Protein Hydrolysates
Source: Foods. 2026 Aug 6;15(15):2764. doi: 10.3390/foods15152764 (PMC13465518; doi:10.3390/foods15152764)
Supplement: Supplementary file 1 [file foods-15-02764-s001.zip › foods-4471739-supplementary.pdf]

Electronic Supplementary Information

Title:

Electronic Supplementary Information for: A Length-Aware C-Terminal Rule for Prioritizing Short ACE-Inhibitory Peptides from Food Protein Hydrolysates

Names of authors:

Mei-Ling Li [1,†], Ying-Jang Lai [2,†], Pei-Yu Wu [1], Jen-Chieh Li [1], Shang-Ming Huang [1,*] and Kuo-Chiang Hsu [1,3,*]

Affiliation and address of authors:

1 Department of Nutrition, China Medical University, No. 100, Sec.1, Jingmao Rd., Beitun Dist., Taichung 406, Taiwan

2 Department of Food Science, National Quemoy University, No. 1, University Rd., Jinning Township, Kinmen 892, Taiwan

3 Department of Food Nutrition and Health Biotechnology, Asia University, 500 Lioufeng Rd., Wufeng, Taichung 413, Taiwan

* Correspondence: zxzxmj2323@mail.cmu.edu.tw (S.-M.H.); kchsu@mail.cmu.edu.tw (K.-C.H.); Tel.: +886-4-22053366 (ext. 7512) (S.-M.H.); +886-4-22053366 (ext. 7522) (K.-C.H.)

† These authors contributed equally to this work.

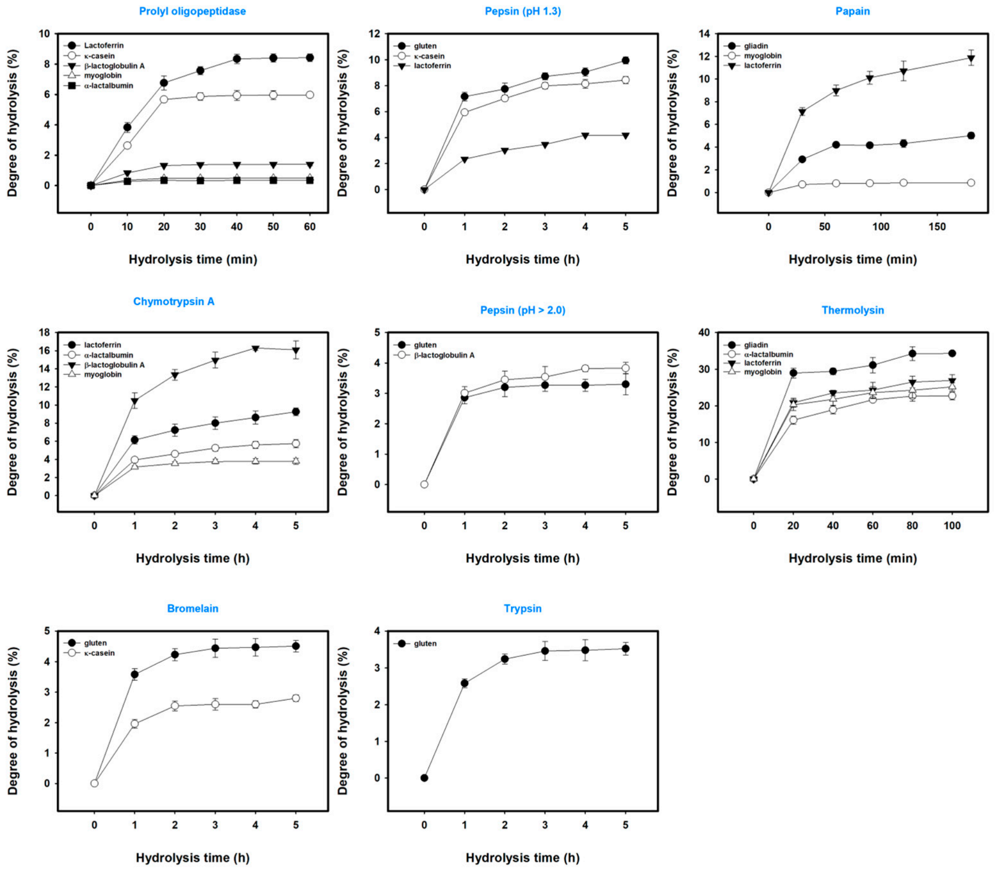

Figure S1. Time-course of degree of hydrolysis (DH, %) for the 24 protein–protease combinations used to construct the rule-development set. Each panel represents a single protease: prolyl oligopeptidase (lactoferrin, κ-casein, β-lactoglobulin A, myoglobin, α-lactalbumin), pepsin at pH 1.3 (gluten, κ-casein, lactoferrin), papain (gliadin, myoglobin, lactoferrin), α-chymotrypsin (lactoferrin, α-lactalbumin, β-lactoglobulin A, myoglobin), pepsin at pH > 2.0 (gluten, β-lactoglobulin A), thermolysin (gliadin, α-lactalbumin, lactoferrin, myoglobin), bromelain (gluten, κ-casein), and trypsin (gluten). Protein substrates (4%, w/v) were hydrolyzed at a 3% (w/w) enzyme-to-substrate ratio under optimal conditions for each protease (see Section 2.3). DH was determined by the TNBS method (Section 2.4). The plateau time at which DH reached a steady state was used as the experimental hydrolysis endpoint to approximate the extensive cleavage assumed in the virtual digestion. Data are expressed as mean ± SD from three independently prepared hydrolysis batches (n = 3).

(A) α-Lactalbumin/Thermolysin

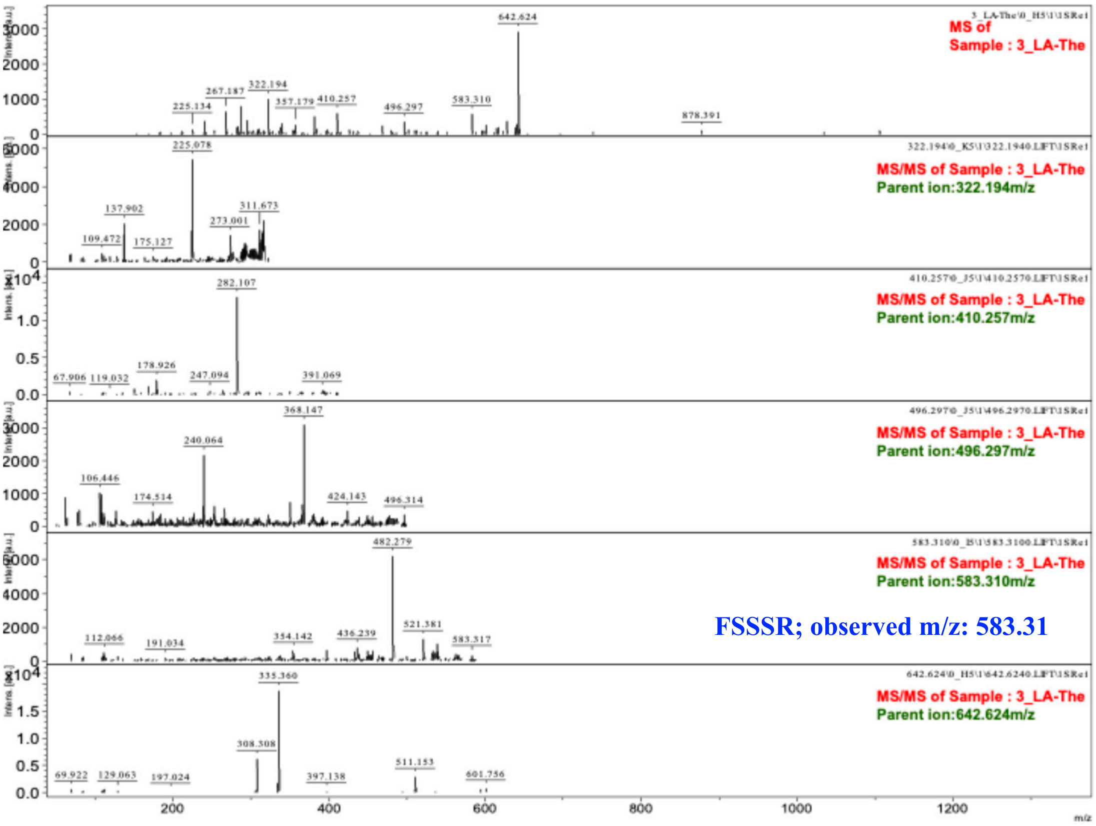

(B) Lactoferrin/Thermolysin

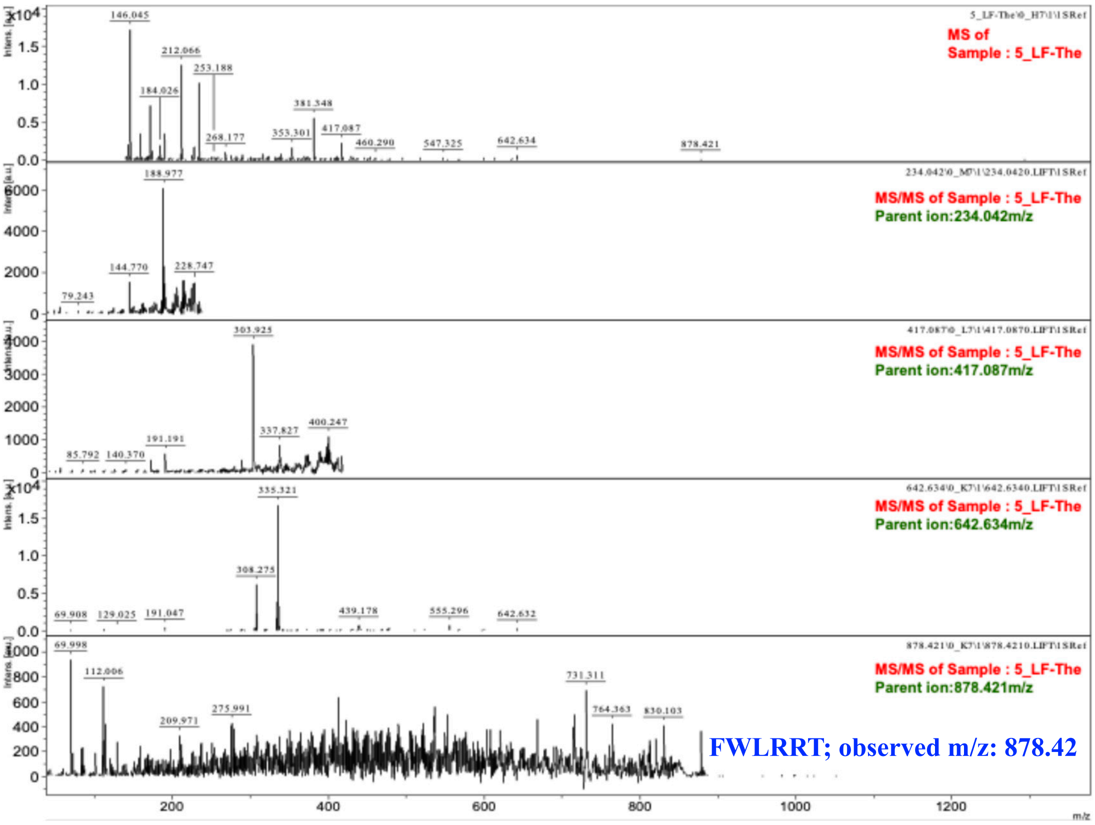

(C) Myoglobin/Thermolysin

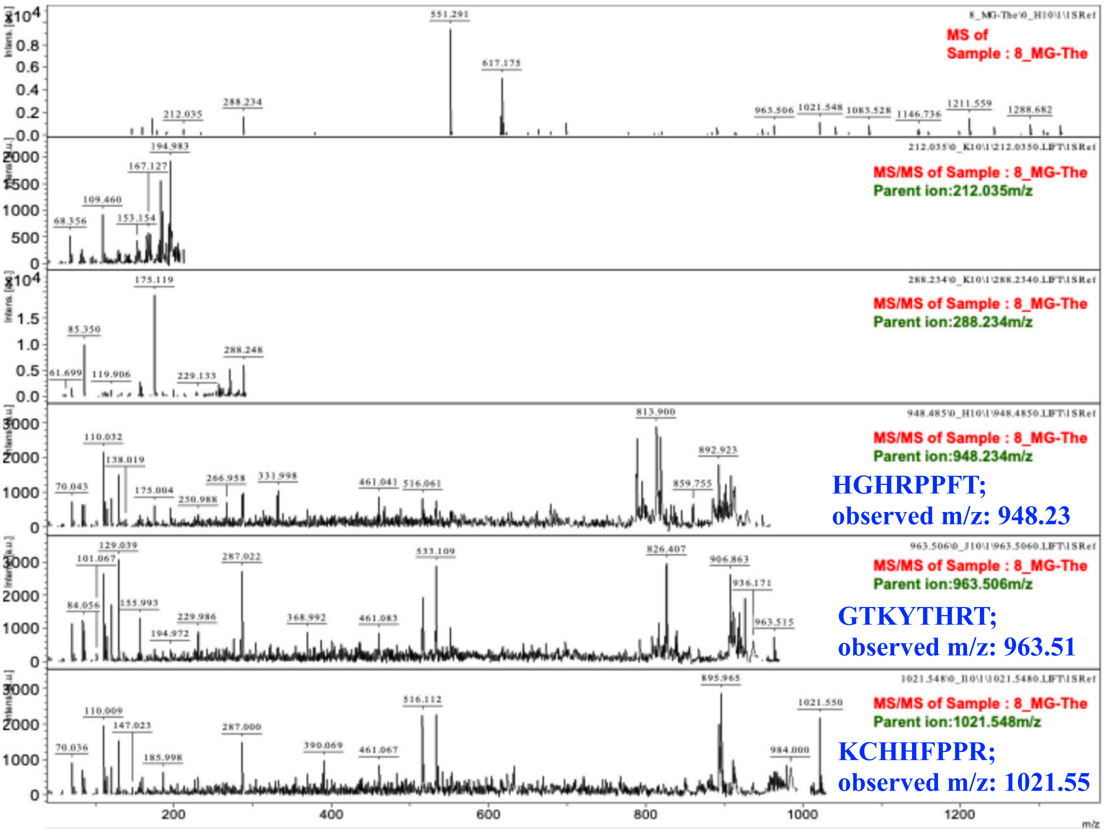

(D) Myoglobin/Papain

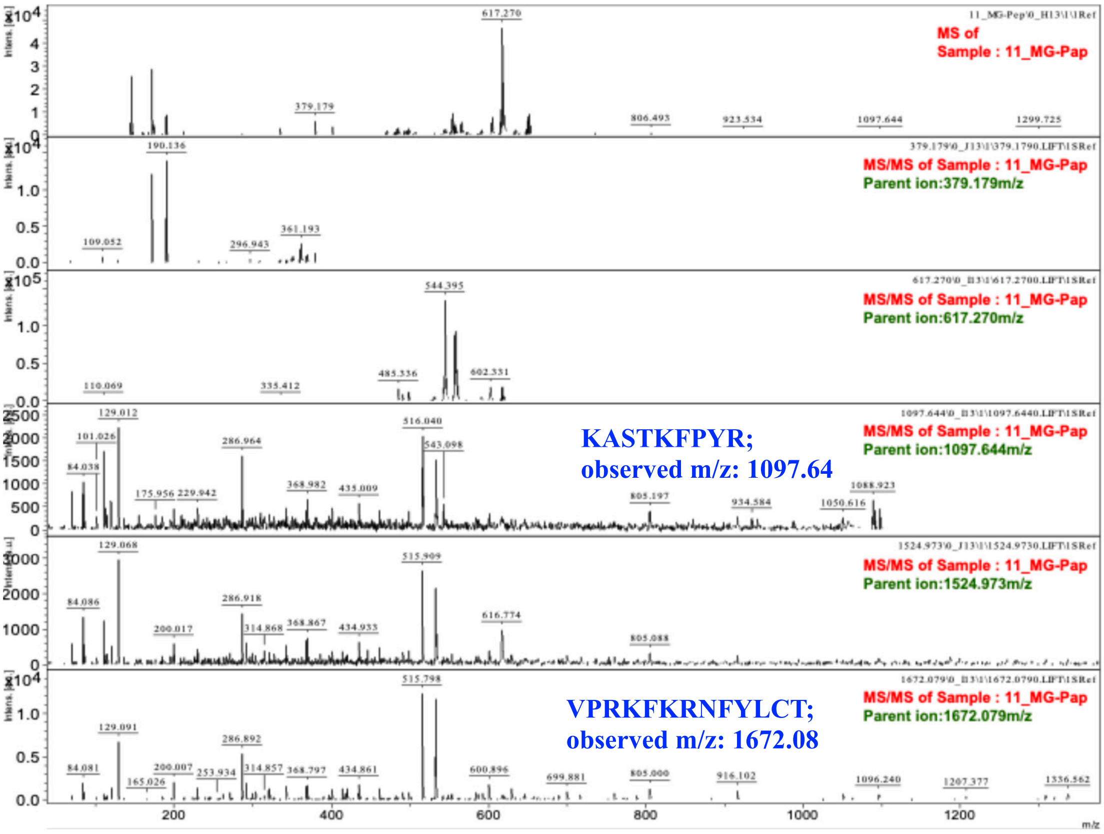

(E) β-Lactoglobulin A /Pepsin (pH > 2.0)

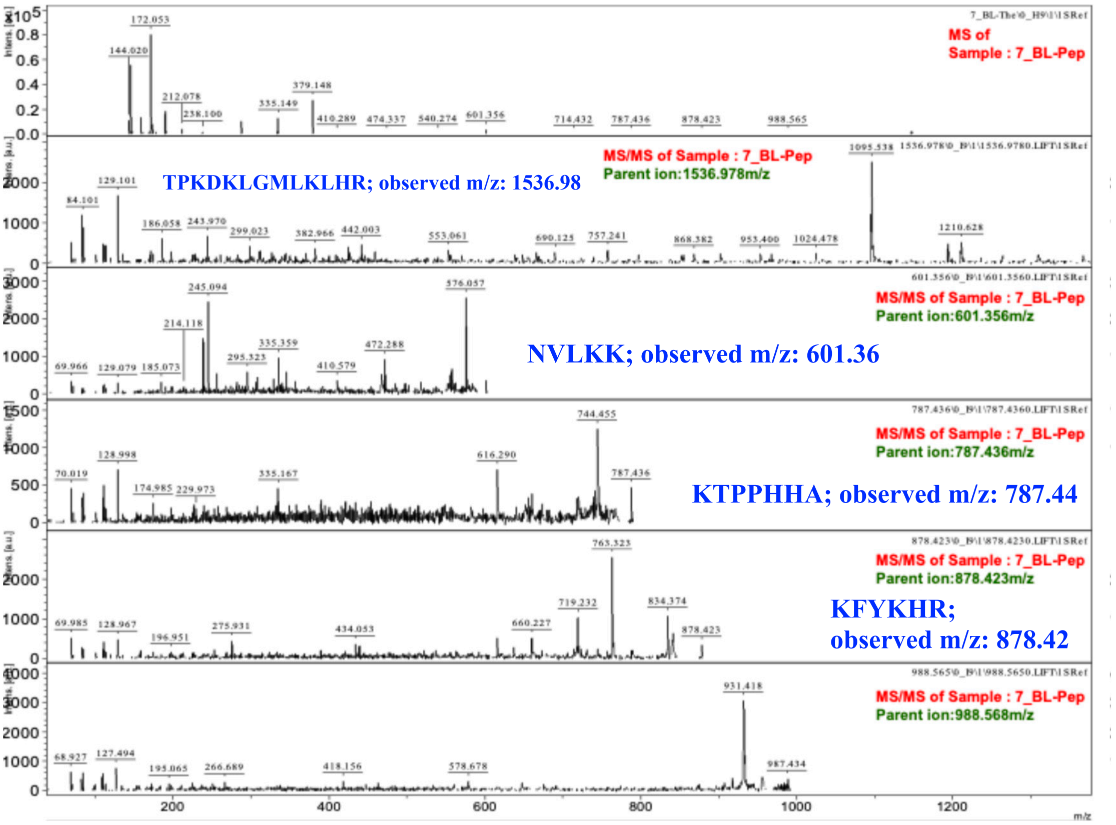

(F) κ-Casein /Pepsin (pH 1.3)

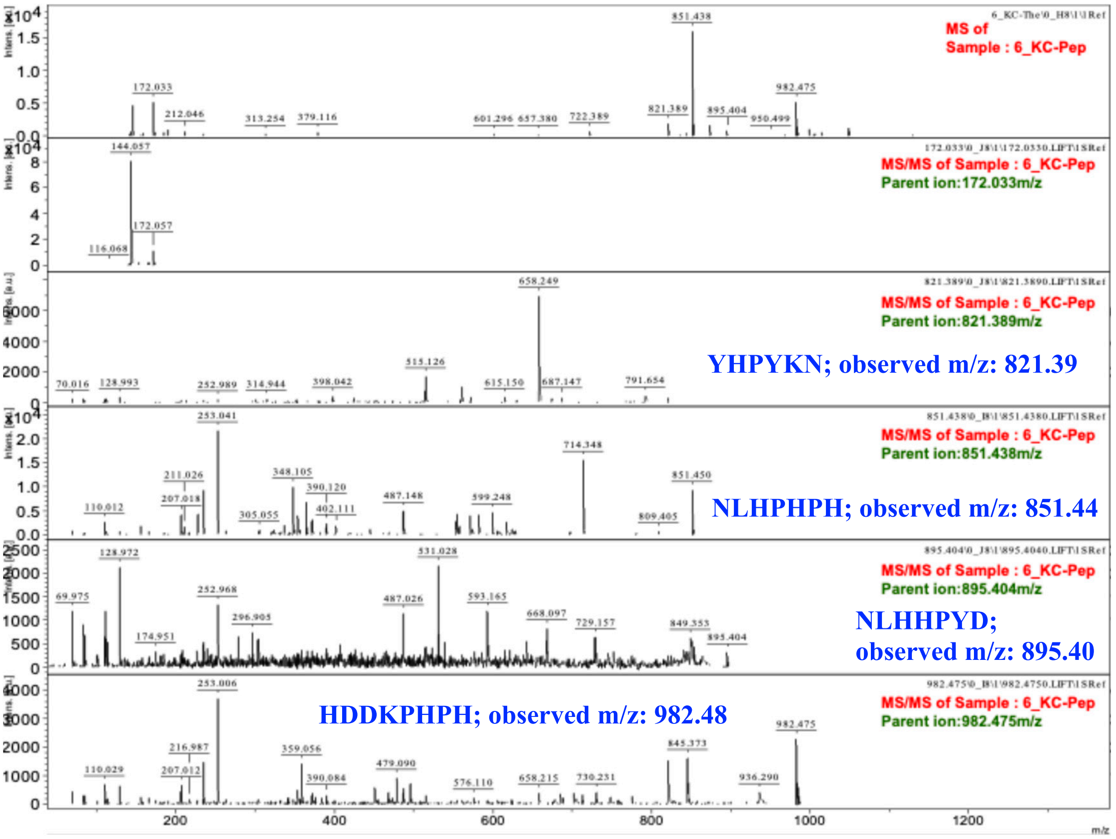

(G)Gluten /Pepsin (pH 1.3)

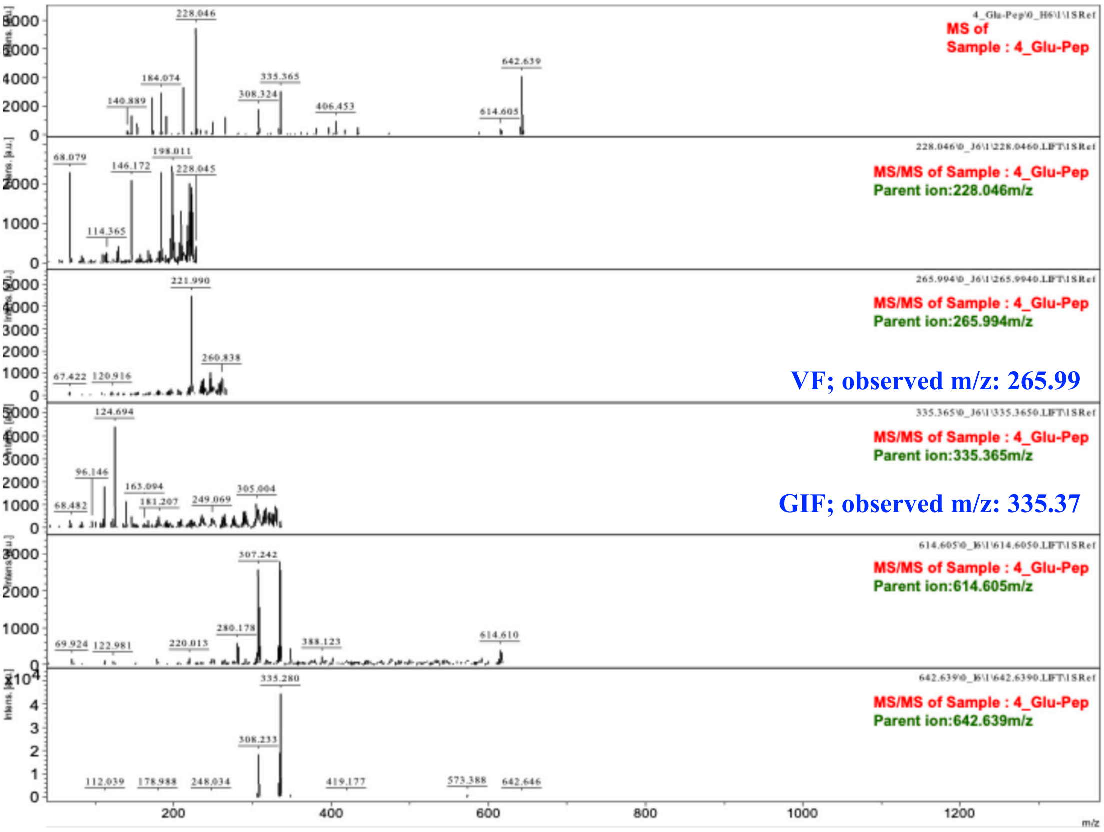

(H)Gluten /Pepsin (pH > 2.0)

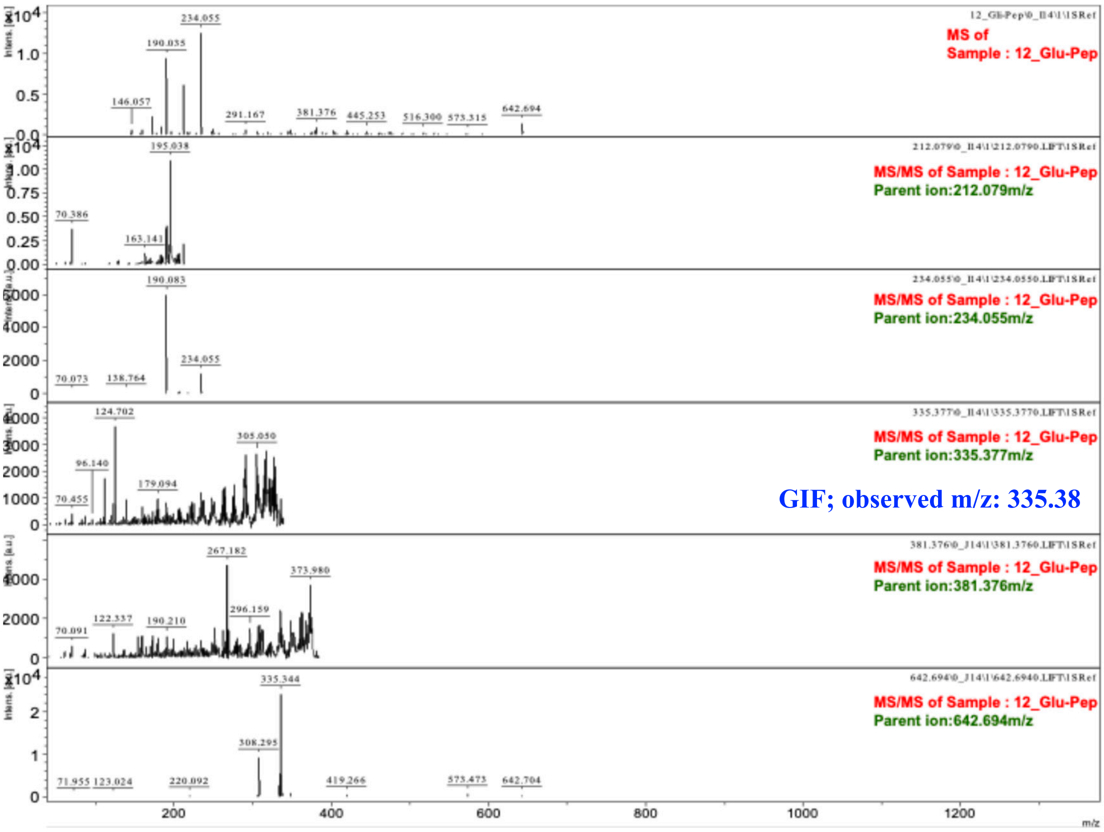

(I) Gluten /Bromelain

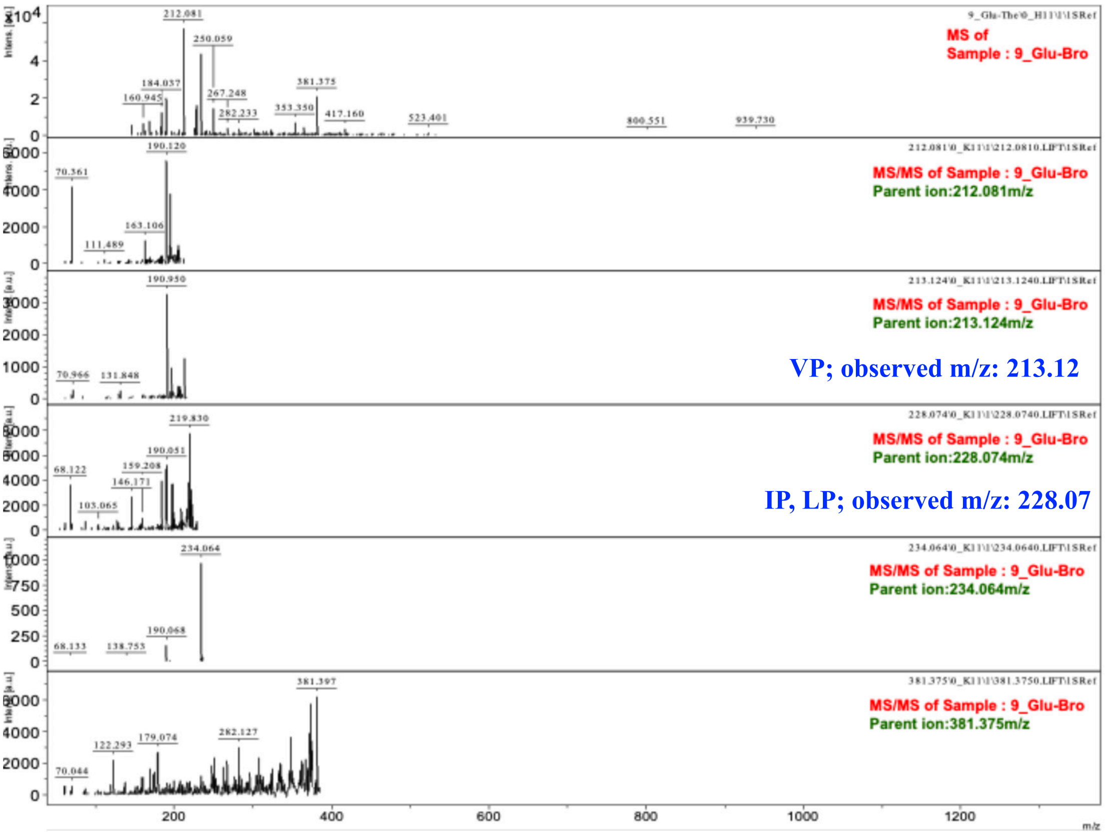

(J) Gliadin /Papain

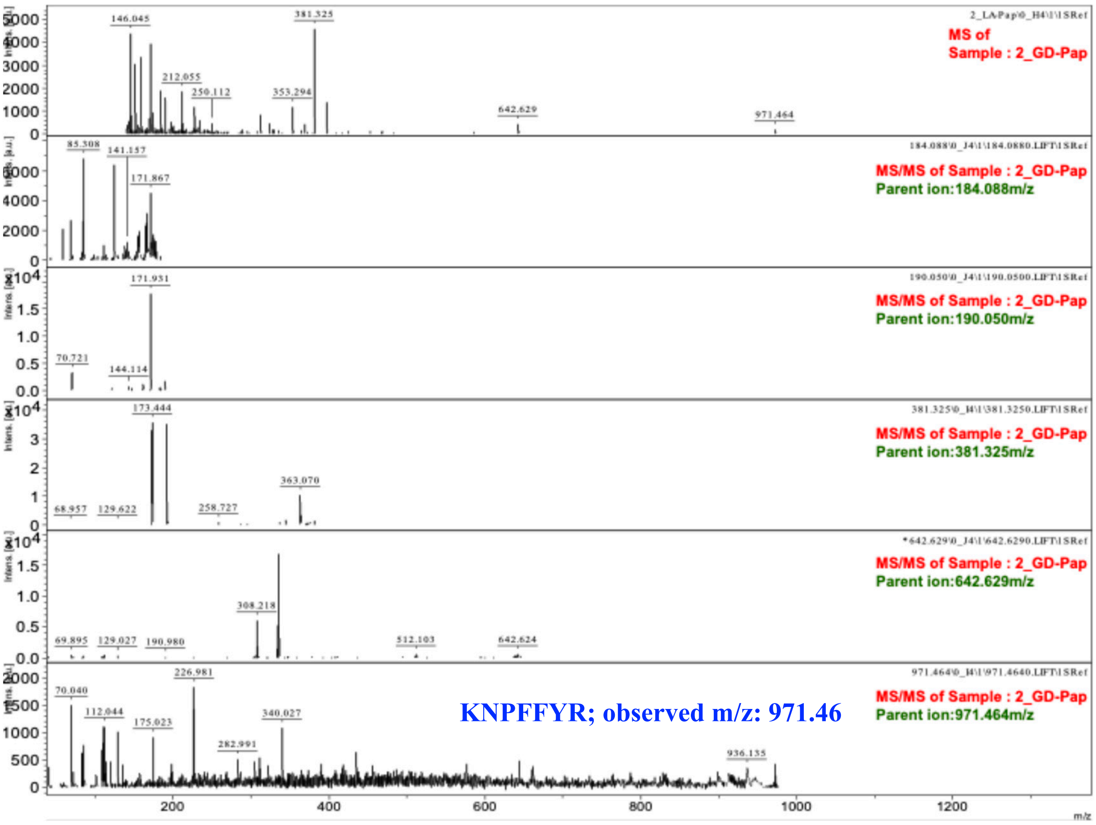

Figure S2. MALDI-TOF/TOF mass spectra supporting peptide assignments in selected protein hydrolysates. Each panel represents one protein–enzyme hydrolysate combination: (A) α-lactalbumin / thermolysin; (B) lactoferrin / thermolysin; (C) myoglobin / thermolysin; (D) myoglobin / papain; (E) β-lactoglobulin A / pepsin (pH > 2.0); (F) κ-casein / pepsin (pH 1.3); (G) gluten / pepsin (pH 1.3); (H) gluten / pepsin (pH > 2.0); (I) gluten / bromelain; (J) gliadin / papain. For peptides of 5–13 residues, annotated peaks showed acceptable agreement with theoretical monoisotopic $[M+H]^+$ values and were cross-referenced against the corresponding in silico digestions (Table S12). The five short candidates (VF, GIF, LP/IP, and VP) occurred in the matrix-dominated low-mass region; because their observed features did not provide sufficiently accurate $[M+H]^+$ matching, they are presented as putative candidate-associated features rather than definitive precursor-ion identifications. LP and IP are isobaric and the shared feature cannot resolve either the individual identity or the simultaneous presence of both isomers. Spectra were acquired on a Bruker UltraFlex III MALDI-TOF/TOF; parameters in Table S6. Complete peptide-assignment data are provided in Table S12.

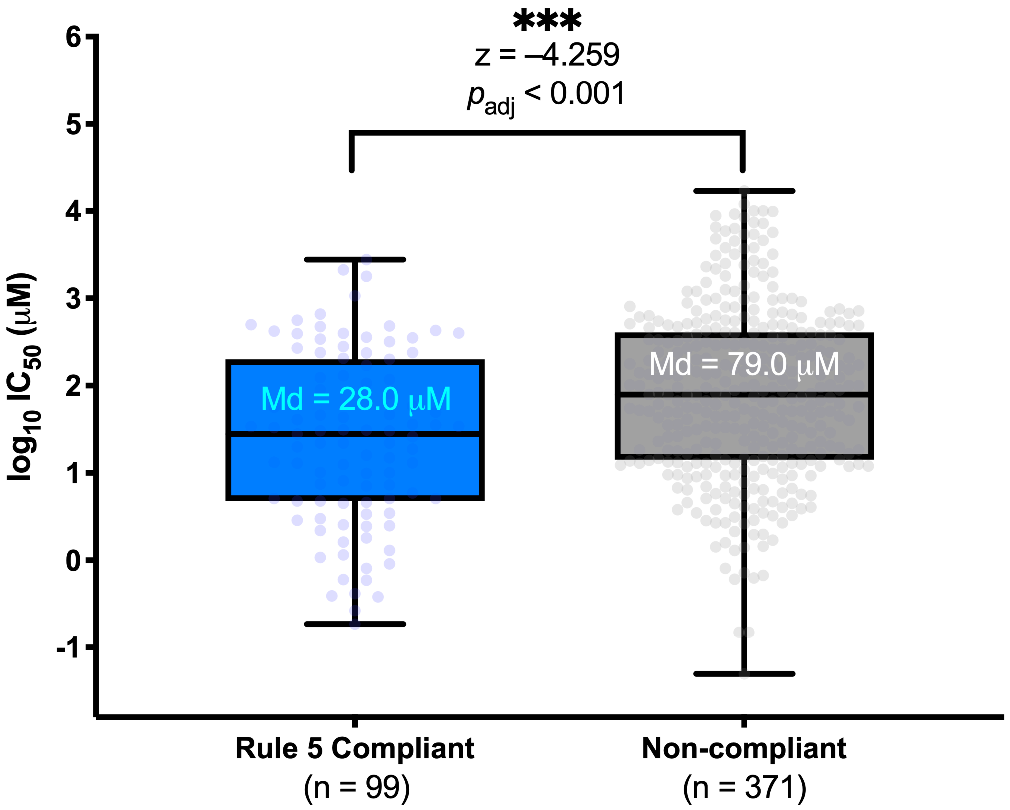

Figure S3. Distribution of $\log_{10}$-transformed IC_{50} values for Rule 5-compliant (n = 144) and non-compliant (n = 487) ACE-inhibitory peptides within the exploratory 2–4-residue comparison window compiled from AHTPDB, BIOPEP-UWM, and MBPDB. Box plots show median (horizontal line), interquartile range (box), and whiskers (1.5× IQR); individual data points are overlaid with jitter. Md, median IC_{50} in μM. **$p < 0.01$, Mann–Whitney U test (z = −2.991); note: after Benjamini–Hochberg correction across 87 length windows, this 2–4 aa window did not retain significance ($p_{adj} = 0.081$). The statistically supported applicability domain is ≤3 aa (see main text Section 3.5 and Table S15).

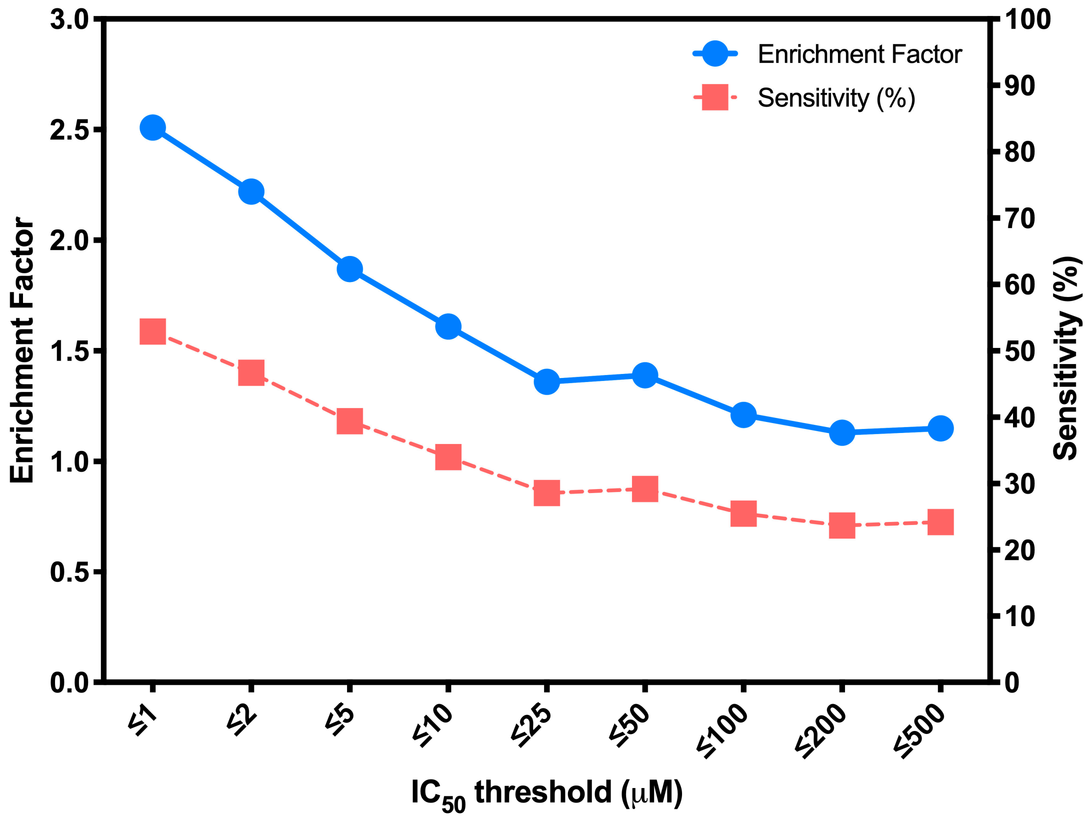

Figure S4. Enrichment factor and sensitivity of Rule 5 as a first-pass enrichment filter across IC_{50} potency thresholds for the 2–3 aa applicability domain (n = 470; $p_{adj} < 0.001$, BH-FDR corrected). Left axis (blue circles, solid line): enrichment factor = (fraction of potent peptides in Rule 5–compliant group) / (fraction in the full unscreened set). Right axis (red squares, dashed line): sensitivity = proportion of all potent peptides captured by Rule 5. Enrichment factor consistently exceeds 1.0 across all thresholds, with maximal enrichment at the most stringent cutoff ($IC_{50} \leq 1$ μM: EF = 2.5×). Rule 5 selects 99/470 (21.1%) of peptides in this domain.

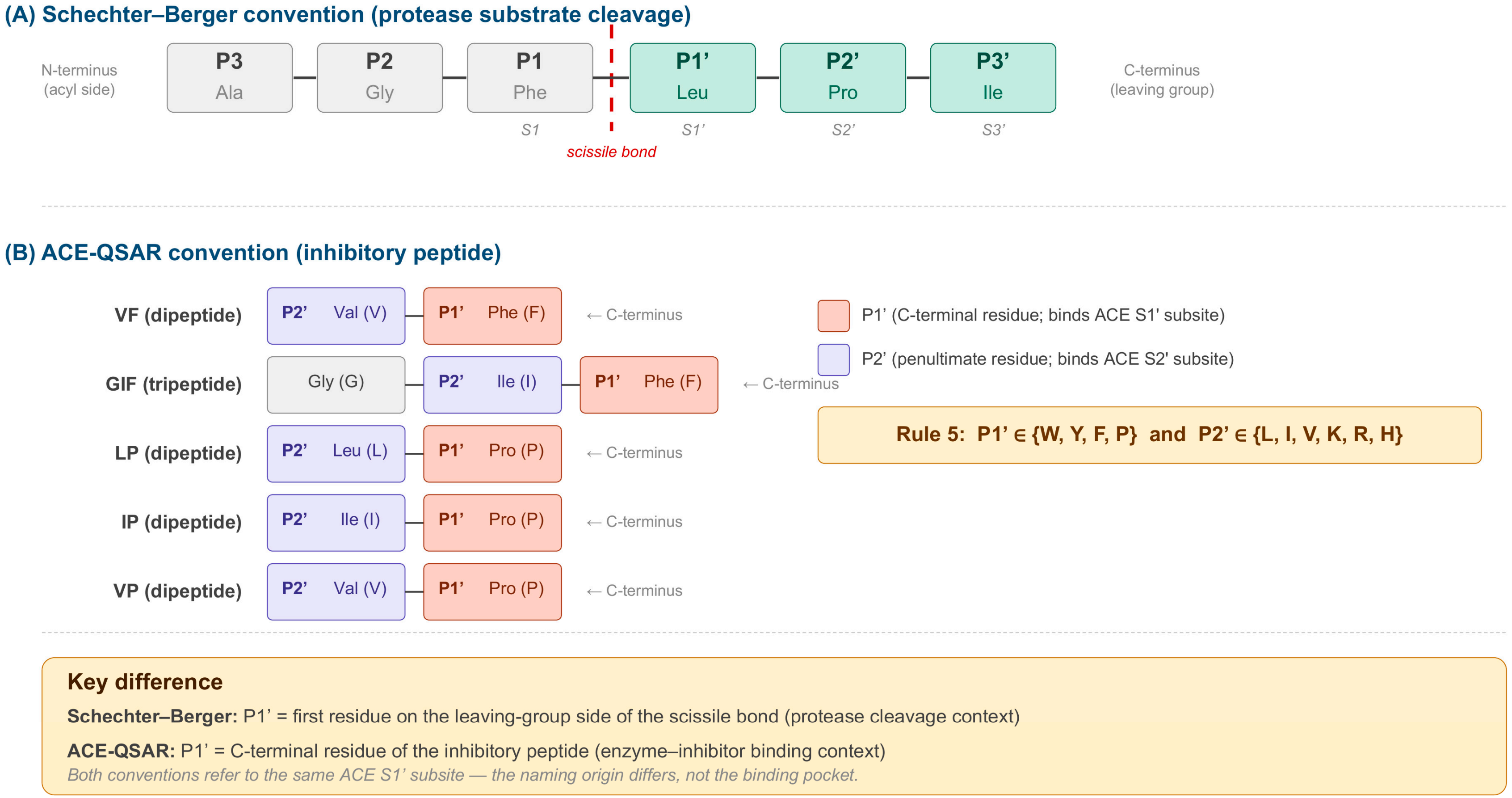

Figure S5. Comparison of positional nomenclature conventions for ACE-inhibitory peptides.

Table S1. List of 84 commercially available food proteins selected from the BIOPEP-UWM database for *in silico* analysis.

No.	BIOPEP ID	Protein name	Length (aa)	Amino Acid Sequence
1	1086	Alpha S1-casein gen. Var. A, bovine *(Bos taurus)*	186	RPKHPIKHQGLPQPFPQVFGKEKVNELSKDIGSESTEDQAMEDIKEMEAESISSSEEIVPNSVEQKHIQKEDVPSERYLGYLEQLLRLKKYKVPQLEIVPNSAEERLHSMKQGIHAQQKE PMIGVNQELAYFYPELFRQFYQLDAYPSGAWYYVPLGTQYTDAPSFSDIPNPIGSENSEKTTMPLW
2	1087	Alpha-S1-casein gen. Var. B, bovine *(Bos taurus)*	199	RPKHPIKHQGLPQEVLNENLLRFFVAPFPQVFGKEKVNELSKDIGSESTEDQAMEDIKEMEAESISSSEEIVPNSVEQKHIQKEDVPSERYLGYLEQLLRLKKYKVPQLEIVPNSAEERL HSMKQGIHAQQKEPMIGVNQELAYFYPELFRQFYQLDAYPSGAWYYVPLGTQYTDAPSFSDIPNPIGSENSEKTTMPLW
3	1088	Alpha-S1-casein gen. Var. C, bovine *(Bos taurus)*	199	RPKHPIKHQGLPQEVLNENLLRFFVAPFPQVFGKEKVNELSKDIGSESTEDQAMEDIKEMEAESISSSEEIVPNSVEQKHIQKEDVPSERYLGYLEQLLRLKKYKVPQLEIVPNSAEERL HSMKQGIHAQQKEPMIGVNQELAYFYPELFRQFYQLDAYPSGAWYYVPLGTQYTDAPSFSDIPNPIGSENSGKTTMPLW
4	1089	Alpha-S1-casein gen. Var. D, precursor, bovine *(Bos taurus)*	214	MKLLILTCLVAVALARPKHPIKHQGLPQEVLNENLLRFFVAPFPEVFGKEKVNELSKDIGSESTEDQAMEDIKQMEAESISSSEEIVPNSVEQKHIQKEDVPSERYLGYLEQLLRLKKYK VPQLEIVPNSAEERLHSMKEGIHAQQKEPMIGVNQELAYFYPELFRQFYQLDAYPSGAWYYVPLGTQYTDAPSFSDIPNPIGSENSEKTTMPLW
5	1090	Alpha-S2-casein gen. Var. A, precursor, bovine *(Bos taurus)*	222	MKFFIFTCLLAVALAKNTMEHVSSSEESIISQETYKQEKNMAINPSKENLCSTFCKEVVRNANEEEYSIGSSSEESAEVATEEVKITVDDKHYQKALNEINQFYQKFPQYLQYLYQGPIV LNPWDQVKRNAVPITPTLNREQLSTSEENSKKTVDMESTEVFTKKTKLTEEEKNRLNFLKKISQRYQKFALPQYLKTVYQHQKAMKPWIQPKTKVIPYVRYL
6	1753	Ovalbumin, egg albumin, chicken *(Gallus gallus)*	386	MGSIGAASMEFCFDVFKELKVHHANENIFYCPIAIMSALAMVYLGAKDSTRTQINKVVRFDKLPGFGDSIEAQCGTSVNVHSSLRDILNQITKPNDVYSFSLASRLYAEERYPILPEYLQ CVKELYRGGLEPINFQTAADQARELINSWVESQTNGIIRNVLQPSSVDSQTAMVLVNAIVFKGLWEKAFKDEDTQAMPFRVTEQESKPVQMMYQIGLFRVASMASEKMKILELPFASGTM SMLVLLPDEVSGLEQLESIINFEKLTEWTSSNVMEERKIKVYLPRMKMEEKYNLTSVLMAMGITDVFSSSANLSGISSAESLKISQAVHAAHAEINEAGREVVGSAEAGVDAASVSEEFR ADHPFLFCIKHIATNAVLFFGRCVSP
7	1277	Beta-actin *(Bos taurus)*	375	MDDDIAALVVDNGSGMCKAGFAGDDAPRAVFPSIVGRPRHQGVMVGMGQKDSYVGDEAQSKRGILTLKYPIEHGIVTNWDDMEKIWHHTFYNELRVAPEEHPVLLTEAPLNPKANREKMT QIMFETFNTPAMYVAIQAVLSLYASGRTTGIVMDSGDGVTHTVPIYEGYALPHAILRLDLAGRDLTDYLMKILTERGYSFTTTAEREIVRDIKEKLCYVALDFEQEMATAASSSSLEKSY ELPDGQVITIGNERFRCPEALFQPSFLGMESCGIHETTFNSIMKCDVDIRKDLYANTVLSGGTTMYPGIADRMQKEITALAPSTMKIKIIAPPERKYSVWIGGSILASLSTFQQMWISKQ EYDESGPSIVHRKCF
8	1239	Precursor of beta-conglycinin, alpha' chain, soybean *(Glycine max)*	639	MMRARFPLLLLGVVFLASVSVSFGIAYWEKQNPSHNKCLRSCNSEKDSYRNQACHARCNLLKVEEEEECEEGQIPRPRPQHPERERQQHGEKEEDEGEQPRPFPFPRPRQPHQEEEHEQK EEHEWHRKEEKHGGKGSEEEQDEREHPRPHQPHQKEEEKHEWQHKQEKHQGKESEEEEEDQDEDEEQDKESQESEGSESQREPRRHKNKNPFHFNSKRFQTLFKNQYGHVRVLQRFNKRS QQLQNLRDYRILEFNSKPNTLLLPHHADADYLIVILNGTAILTLVNNDDRDSYNLQSGDALRVPAGTTFYVVNPDNDENLRMIAGTTFYVVNPDNDENLRMITLAIPVNKPGRFESFFLS STQAQQSYLQGFSKNILEASYDTKFEEINKVLFGREEGQQQGEERLQESVIVEISKKQIRELSKHAKSSSRKTISSEDKPFNLGSRDPIYSNKLGKLFEITQRNPQLRDLDVFLSVVDMN EGALFLPHFNSKAIVVLVINEGEANIELVGIKEQQQRQQQEEQPLEVRKYRAELSEQDIFVIPAGYPVMVNATSDLNFFAFGINAENNQRNFLAGSKDNVISQIPSQVQELAFPRSAKDI ENLIKSQSESYFVDAQPQQKEEGNKGRKGPLSSILRAFY
9	1775	Beta-conglycinin, alpha chain, precursor, soybean *(Glycine max)*	605	MMRARFPLLLLGLVFLASVSVSFGIAYWEKENPKHNKCLQSCNSERDSYRNQACHARCNLLKVEKEECEEGEIPRPRPRPQHPEREPQQPGEKEEDEDEQPRPIPFPRPQPRQEEEHEQR EEQEWPRKEEKRGEKGSEEEDEDEDEEQDERQFPFPRPPHQKEERNEEEDEDEEQQRESEESEDSELRRHKNKNPFLFGSNRFETLFKNQYGRIRVLQRFNQRSPQLQNLRDYRILEFNS KPNTLLLPNHADADYLIVILNGTAILSLVNNDDRDSYRLQSGDALRVPSGTTYYVVNPDNNENLRLITLAIPVNKPGRFESFFLSSTEAQQSYLQGFSRNILEASYDTKFEEINKVLFSR EEGQQQGEQRLQESVIVEISKEQIRALSKRAKSSSRKTISSEDKPFNLRSRDPIYSNKLGKFFEITPEKNPQLRDLDIFLSIVDMNEGALLLPHFNSKAIVILVINEGDANIELVGLKEQ QQEQQQEEQPLEVRKYRAELSEQDIFVIPAGYPVVVNATSNLNFFAIGINAENNQRNFLAGSQDNVISQIPSQVQELAFPGSAQAVEKLLKNQRESYFVDAQPKKKEEGNKGRKGPLSSI LRAFY
10	1317	Gamma-gliadin, wheat *(Triticum aestivum)*	282	MNIQVDPSSQVPWPQQQPFPQPHQPFSQQPQQTFPQPQQTFPHQPQQQFSQPQQPQQQFIQPQQPFPQQPQQTYPQRPQQPFPQTQQPQQPFPQSQQPQQPFPQPQQQFPQPQQPQQSFP QQQPSLIQQSLQQQLNPCKNFLLQQCKPVSLVSSLWSMILPRSDCQVMRQQCCQQLAQIPQQLQCAAIHSIVHSIIMQQEQQEQRQGVQILVPLSQQQQVGQGTLVQGQGIIQPQQPAQL EVIRSLVLQTLATMCNVYVPPYCSTIRAPFASIVAGIGGQYR
11	1319	Gamma-gliadin, wheat *(Triticum aestivum)*	311	MNIQVDPSSQVQWLQQQLVPQLQQPLSQQPQQTFPQPQQTFPHQPQQQVPQPQQPQQPFLQPQQPFPQQPQQPFPQTQQPQQPFPQQPQQPFPQTQQPQQPFPQQPQQPFPQTQQPQQPF PQLQQPQQPFPQPQQQLPQPQQPQQSFPQQQRPFIQPSLQQQLNPCKNILLQQCKPASLVSSLWSIIWPQSDCQVMRQQCCQQLAQIPQQLQCAAIHSVVHSIIMQQQQQQQQQQGMHIF LPLSQQQQVGQGSLVQGQGIIQPQQPAQLEAIRSLVLQTLPSMCNVYVPPECSIMRAPFASIVAGIGGQYR
12	1420	Alpha-gliadin, wheat *(Triticum aestivum)*	290	MVRVPVPQLQPQNPSQQQPQEQVPLVQQQQFPGQQQPFPPQQPYPQPQPFPSQQPYLQLQPFPQPQLPYPQPQLPYPQPQLPYPQPQPFRPQQPYPQSQPQYSQPQQPISQQQQQQQQQQ QQKQQQQQQQQILQQILQQQLIPCRDVVLQQHSIAYGSSQVLQQSTYQLVQQLCCQQLWQIPEQSRCQAIHNVVHAIILHQQQQQQQQQQQQPLSQVSFQQPQQQYPSGQGSFQPSQQNP QAQGSVQPQQLPQFEEIRNLALETLPAMCNVYIPPYCTIAPVGIFGTNYR
13	1329	Glutenin, low molecular weight *(LMW)*, wheat (Triticum aestivum)	388	MKTFLVFALLALAAASAVAQISQQQQPPPFSQQQQPPFSQQQQPPFSQQQQSPFSQQQQQPPFSQQQQPPFSQQPLISQQQQLPFSQQQQPQFSQQQQPPYSQQQQPPYSQQQQPPFSQQ QQPPFSQQQQPSFSQQQQQPPFTQQQQPPFSQQSPISQQQQQQQQQQQQPFTQQQQPPFSQQPPISQQQQPPFSQQQQPPFSQQQQIPVIHPSVLQQLNPCKVFLQQQCIPVAMQRCLAR SQMLQQSICHVMQRQCCQQLRQIPEQSRHESIRAIIYSIILQQQQQQQQQQQQQQQQGQSIIQYQQQPPQQLGQCVSQPQQQLQQQLGQQPQQQQLAHGTFLQPHQIAQLEVMTSIALRTL PRMCSVNVPLYETTTSVPLGVGIGVGVY
14	1283	Glutenin, low molecular weight *(LMW)*, precursor, wheat (Triticum aestivum) subunit	356	MKTFLVFALLALAAASAVAQISQQQQAPPFSQQQQPPFSQQQQPPFSQQQQSPFSQQQQQPPFAQQQQPPFSQQPPISQQQQPPFSQQQQPQFSQQQQPPYSQQQQPPYSQQQQPPFSQQ QQPPFSQQQQQPPFTQQQQQQQQQQPFTQQQQPPFSQQPPISQQQQPPFLQQRPPFSRQQQIPVIHPSVLQQLNPCKVFLQQQCIPVAMQRCLARSQMLQQSICHVMQQQCCQQLRQIP EQSRHESIRAIIYSIILQQQQQQQQQQQQQQQGQSIIQYQQQQPQQLGQCVSQPLQQLQQQLGQQPQQQQLAHQIAQLEVMTSIALRTLPTMCNVNVPLYETTTSVPLGVGIGVGVY
15	1458	Avenin, precursor, oat *(Avena sativa)*	222	MKTFLIIALLAMAVATATATTTVQYNPSEQYQPYPEQQEPFVQQQQPFVQQQQPFVQQQQMFLQPLLQQQLNPCKQFLVQQCSPVAAVPFLRSQILRQAICQVTRQQCCRQLAQIPEQLR CPAIHSVVQSIILQQQQQQQQFIQPQLQQQVFQPQLQLQQQVFQPQLQQQVFQPQLQQVFNQPQMQGQIEGMRAFALQALPAMCDVYVPPQCPVATAPLGGF
16	1451	Avenin-3 precursor, oat *(Avena sativa)*	220	MKTFLIFALLAMAATMATAQFDPSEQYQPYPEQQQPILQQQQMLLQQQQQMLLQQQPLLQVLQQQLNPCRQFLVQQCSPVAVVPFLRSQILQQSSCQVMRQQCCRQLEQIPEQLRCPAIH SVVQAIIMQQQQFFQPQMQQQFFQPQMQQVTQGIFQPQMQQVTQGIFQPQLQQVTQGIFQPQMQGQIEGMRAFALQALPAMCDVYVPPHCPVATAPLGGF
17	1550	13 kda prolamin, precursor, rice *(Oryza sativa)*	156	MKIIFVFALLAIVACNASARFDPLSQSYRQYQLQSHLLLQQQVLSPCSEFVRQQYSIVATPFWQPATFQLINNQVMQQQCCQQLRLVAQQSHYQAISIVQAIVQQLQLQQFSGVYFDQTQ

No.	BIOPEP ID	Protein name	Length (aa)	Amino Acid Sequence
				AQAQTLLTFNLPSICGIYPNYYSAPRSIATVGGVWY
18	1592	Prolamin, rice *(Oryza sativa)*	156	MKIIFFFALLAIAACSASAQFDAVTQVYRQYQLQPHLMLQQQMLSPCGEFVRQQCSTVATPFFQSPVFQLRNCQVMQQQCCQQLRMIAQQSHCQAISSVQAIVQQLRLQQFASVYFDQSQ<br>AQAQAMLALNMPSICGIYPSYNTAPCSIPTVGGIWY
19	1524	Convicilin, precursor, garden pea *(Pisum sativum)*	613	MATTIKSRFPLLLLLGIIFLASVVCVTYANYDEGSEPRVPAQRERGRQEGEKEEKRHGEWRPSYEKEEDEEEGQRERGRQEGEKEEKRHGEWRPSYEKQEDEEEKQKYRYQREKEDEEEK<br>QKYQYQREKKEQKEVQPGRERWEREEDEEQVDEEWRGSQRREDPEERARLRHREERTKRDRRHQREGEEEERSSESQERRNPFLFKSNKFLTLFENENGHIRLLQRFDKRSDLFENLQNY<br>RLVEYRAKPHTIFLPQHIDADLILVVLSGKAILTVLSPNDRNSYNLERGDTIKLPAGTTSYLVNQDDEEDLRLVDLVIPVNGPGKFEAFDLAKNKNQYLRGFSKNILEASYNTRYETIEK<br>VLLEEQEKDRKRRQQGEETDAIVKVSREQIEELKKLAKSSSKKSLPSEFEPINLRSHKPEYSNKFGKLFEITPEKKYPQLQDLDLFVSCVEINEGALMLPHYNSRAIVVLLVNEGKGNLE<br>LLGLKNEQQEREDRKERNNEVQRYEARLSPGDVVIIPAGHPVAITASSNLNLLGFGINAENNERNFLSGSDDNVISQIENPVKELTFPGSVQEINRLIKNQKQSHFANAEPEQKEQGSQG<br>KRSPLSSILGTFY
20	1779	Allergen Ara h 1, clone P17, precursor, peanut *(Arachis hypogaea)*	614	MRGRVSPLMLLLGILVLASVSATQAKSPYRKTENPCAQRCLQSCQQEPDDLKQKACESRCTKLEYDPRCVYDTGATNQRHPPGERTRGRQPGDYDDDRRQPRREEGGRWGPAEPRERERE<br>EDWRQPREDWRRPSHQQPRKIRPEGREGEQEWGTPGSEVREETSRNNPFYFPSRRFSTRYGNQNGRIRVLQRFDQRSKQFQNLQNHRIVQIEARPNTLVLPKHADADNILVIQQGQATVT<br>VANGNNRKSFNLDEGHALRIPSGFISYILNRHDNQNLRVAKISMPVNTPGQFEDFFPASSRDQSSYLQGFSRNTLEAAFNAEFNEIRRVLLEENAGGEQEERGQRRRSTRSSDNEGVIVK<br>VSKEHVQELTKHAKSVSKKGSEEEDITNPINLRDGEPDLSNNFGRLFEVKPDKKNPQLQDLDMMLTCVEIKEGALMLPHFNSKAMVIVVVNKGTGNLELVAVRKEQQQRGRREQEWEEEE<br>EDEEEEGSNREVRRYTARLKEGDVFIMPAAHPVAINASSELHLLGFGINAENNHRIFLAGDKDNVIDQIEKQAKDLAFPGSGEQVEKLIKNQRESHFVSARPQSQSPSSPEKEDQEEENQ<br>GGKGPLLSILKAFN
21	1241	Phaseolin a-type precursor, french bean *(Phaseolus vulgaris)*	436	MMRARVPLLLLGILFLASLSASFATSLREEEESQDNPFYFNSDNSWNTLFKNQYGHIRVLQRFDQQSKRLQNLEDYRLVEFRSKPETLLLPQQADAELLLVVRSGSAILVLVKPDDRREY<br>FFLTQGDNPIFSDNQKIPAGTIFYLVNPDPKEDLRIIQLAMPVNNPQIHEFFLSSTEAQQSYLQEFSKHILEASFNSKFEEINRVLFEEEGQQEEGQQEGVIVNIDSEQIEELSKHAKSS<br>SRKSHSKQDNTIGNEFGNLTERTDNSLNVLISSIEMKEGALFVPHYYSKAIVILVVNEGEAHVELVGPKGNKETLEFESYRAELSKDDVFVIPAAYPVAIKATSNVNFTGFGINANNNNR<br>NLLAGKTDNVISSIGRALDGKDVLGLTFSGSGEEVMKLINKQSGSYFVDGHHHQQEQQKGSHQQEQQKGRKGAFVY
22	1242	Phaseolin b-type precursor, french bean *(Phaseolus vulgaris)*	421	MMRARVPLLLLGILFLASLSASFATSLREEEESQDNPFYFNSDNSWNTLFKNQYGHIRVLQRFDQQSKRLQNLEDYRLVEFRSKPETLLLPQQADAELLLVVRSGSAILVLVKPDDRREY<br>FFLTSDNPIFSDHQKIPAGTIFYLVNPDPKEDLRIIQLAMPVNNPQIHEFFLSSTEAQQSYLQEFSKHILEASFNSKFEEINRVLFEEEGQQEGVIVNIDSEQIKELSKHAKSSSRKSLS<br>KQDNTIGNEFGNLTERTDNSLNVLISSIEMEEGALFVPHYYSKAIVILVVNEGEAHVELVGPKGNKETLEYESYRAELSKDDVFVIPAAYPVAIKATSNVNFTGFGINANNNNRNLLAGK<br>TDNVISSIGRALDGKDVLGLTFSGSGDEVMKLINKQSGSYFVDAHHHQQEQQKGRKGAFVY
23	1468	13S globulin seed storage protein, precursor, common buckwheat *(Fagopyrum esculentum)*	565	MSTKLILSFSLCLMVLSCSAQLLPWRKGQRSRPHRGHQQFHHQCDVQRLTASEPSRRVRSEAGVTEIWDNDTPEFRCAGFVAVRVVIQPGGLLLPSYSNAPYITFVEQGRGVQGVVVPGC<br>PETFQSESEFEYPQSQRDQRSRQSESEESSRGDQRTRQSESEEFSRGDQRTRQSESEEFSRGDQRTRQSESEEFSRGDQRTRQSESEEFSRGDQHQKIFRIRDGDVIPSPAGVVQWTHND<br>GDNDLISITLYDANSFQNQLDGNVRNFFLAGQSKQSREDRRSQRQTREEGSDRQSRESDDDEALLEANILTGFQDEILQEIFRNVDQETISKLRGDNDQRGFIVQARDLKLRVPEEYEEE<br>LQRERGDRKRGGSGRSNGLEQAFCNLKFKQNVNRPSRADVFNPRAGRINTVNSNNLPILEFIQLSAQHVVLYKNAILGPRWNLNAHSALYVTRGEGRVQVVGDEGRSVFDDNVQRGQILV<br>VPQGFAVVLKAGREGLEWVELKNDDNAITSPIAGKTSVLRAIPVEVLANSYDISTKEAFRLKNGRQEVEVFLPFQSRDEKERERF
24	1149	Kafirin PGK1 precursor, sorghum *(Sorghum vulgare)*	269	MATKIFALLALHALLVSGTTAAIIPQCSLAPNAIIPQFIPPVTALGNEHLAVQAYPGQQVLSASILQQPIAQLQQQSLAHLTVQTITAQQQQQQQQQQQQQFLSSLSALAVANQAAYLQ<br>QQLLTSNPHSLANAAAYQQQQQLQLAMANPTAYVQQQLLLSNPQAATNAATYLQQQQFQQILPALSQLRMANPTAYLQQQQLLPINQLALANTDAYLQQQQLLPVNPLVVANPLVAAFLQ<br>QQQLSSFNQISLVNPALSWQQPIIGGAIF
25	1353	Glutenin, high molecular weight *(HMW)*, wheat (*Triticum aestivum*), subunit	971	MAKRLVLFVAVVVALVALTVAEGEASEQLQCERELQELQERELKACQQVMDQQLRDISPECHPVVVSPVAGQYEQQIVMPPKGGSFYPGETTPPQQLQQRIFWGIPALLKRYYPSVTSPQ<br>QVSYYPGQASPQRPGQGQQPGQGQQSGQGQQGYYPTSPQQPGQWQQPEQGQPGYYPTSPQQPGQLQQPAQGQQPGQGQQGRQPGQGQPGYYPTSSQLQPGQLQQPAQGQQGQQPGQGQQG<br>QQPGQGQQPGQGQQGQQPGQGQQPGQGQQGQQLGQGQQGYYPTSLQQSGQGQPGYYPTSLQQLGQGQSGYYPTSPQQPGQGQQPGQLQQPAQGQQPEQGQQGQQPGQGQQGQQPGQGQQP<br>GQGQPGYYPTSPQQSGQGQPGYYPTSSQQPTQSQQPGQGQQGQQVGQGQQAQQPGQGQQLGQGQPGYYPTSPLQSGQGQSGYYPTSPQQPGQGQQPGQLQQPAQGQQPEQGQQGQQPGQG<br>QQGQQPGQGQQPGQGQPGYYPTSPQQSGQGQPGYYPTSSQQPTQSQQPGQGQQGQQVGQGQQAQQPGQGQQPGQGQPGYYPTSPLQSGQGQPGYYLTSPQQSGQGQQPGQLQQSAQGQKG<br>QQPGQGQQPGQGQQGQQPGQGQQGQQPGQGQPGYYPTSPQQSGQGQQPGQWQQPGQGQPGYYPTSPLQPGQGQPGYDPTSPQQPGQGQQPGQLQQPAQGQQGQQLAQGQQGQQPAQVQQ<br>GQQPAQGQQGQQLGQGQQGQQPGQGQQPAQGQQGQQPGQGQQGQQPGQGQQPGQGQPWYYPTSPQESGQGQQPGQWQQPGQWQQPGQGQPGYYLTSPLQLGQGQQGYYPTSLQQPGQGQQ<br>PGQWQQSGQGQHGYYPTSPQLSGQGQRPGQWLQPGQGQQGYYPTSPQQSGQGQQLGQWLQPGQGQQGYYPTSLQQTGQGQQSGQGQQGYYSSYHVSVEHQAASLKVAKAQQLAAQLPAMC<br>RLEGGDALSASQ
26	1322	Glutenin, high molecular weight *(HMW)*, wheat (*Triticum aestivum*) subunit 1Dx2.1	836	MAKRLVLFVAVVVALVALTVAEGEASEQLQCERELQELQERELKACQQVMDQQLRDISPECHPVVVSPVAGQYERQIVVPPKGGSFYPGETTPPQQLQQRIFWGIPALLKRYYPSVTSPQ<br>QVSYYPGQASPQRPGQGQQPGQGQQSGQGQQGYYPTSPQQPGQWQQPEQGQPGYYPTSPQQPGQLQQPAQGQQPGQGQQGQQPGQGQPGYYPTSSQLQPGQLQQPAQGQQGQQPGQGQQG<br>QQPGQGQQPGQGQQGQQPGQGQQPGQGQQGQQLGQGQQGYYPTSLQQSGQGQPGYYPTSLQQLGQGQSGYYPTSPQQPGQGQQPGQLQQPAQGQQPGQGQQGQQPGQGQQGQQPGQGQQP<br>GQGQPGYYPTSPQQSGQGQPGYYPTSSQQPTQSQQPGQGQQGQQVGQGQQAQQPGQGQQPGQGQPGYYPTSPQQSGQGQPGYYLTSPQQSGQGQQPGQLQQSAQGQKGQQPGQGQQPGRG<br>QQGQQPGQGQQGQQPGQGQPGYYPTSPQQSGQGQQPGQWQQPGQGQPGYYPTSPLQPGQGQPGYDPTSPQQPGQGQQPGQLQQPAQGQQGQQLAQGQQGQQPAQVQQEQQPAQGQQGQQL<br>GQGQQGQQPGQGQQGQQPAQGQQGQQPGQGQQGQQPGQGQQPGQGQPWYYPTSPQESGQGQQPGQWQQPGQGQPGYYLTSPLQLGQGQQGYYPTSLQQPGQGQQPGQWQQSGQGQHEYYP<br>TSPQLSGQGQRPGQWLQPGQGQQGYYPTSPQQSGQGQQLGQWLQPGQGQQGYYPTSLQQTGQGQQSGQGQQGYYSSYHVSVEHQAASLKVAKAQQLAAQLPAMCRLEGGDALSASQ
27	1595	Omega secalin, rye *(Secale cereale)*	357	MKTFLIFVLAMTMSIITTARQLNPSEQELQSPQQPVPKEQSYPQQPYPSHQPFPTPQQYSPYQPQQPFPQPQQPTPIQPQQPFPQQPQQPFSQPQQQLPLQPQQPFPQPQQPIPQQPQQS<br>FPQQPQRPEQQFPQQPQQIIPQQTQQPFPLQPQQPFPQQPQRPFAQQPEQIISQQPFPLQPQQPFSQPQQPFPQQPEQIIPQQPQQPSPLQPQQPFSQQPQRPQQPFPQQPQQIIPQQPQ<br>QPFPLQPQQPVPQQPQRPFGQQPEQIISQRPQQPFLLQPQQPFSQPQQPFLQQPGQIIPQQPQQPFPLQPQQPFPQQPEQIISEQPQQPFLLQPQQPSPQQPQLPFPQPQQPFVVVV
28	1627	C-hordein, barley *(Hordeum vulgare)*	310	MKTFLTFVLLAMVMSIVTTARQLNPSSQELQSPQQSYLQQPYPQNPYLPQQPFPVQQPFHTPQQYFPYLPEELSPQYQIPTPLQPQQPFPQQPQQPLPRPQQPFPWQPQQPFPQPQQPIP<br>YQPQQPFNQQPQQIISQQPQQPFPQQPQQPFPQPQQPFPWQPQQPFPQPQQPFPLQPQQPFPWQPQQPFPQPQQPIAHQPQQPFSFSQQPQQPFPLQPQQPFPQQPQQPFPQQPQQIIFQ<br>QPQQSYPVQPQQPFPQPQPVPQQRPQQASPLQPQQPFPQGSEQIIPQQPQQPFPLQPHQPYTQQTIWSMV
29	1632	D-Hordein, barley *(Hordeum vulgare)*	757	MAKRLVLFVAVIVALVALTTAEREINGNNIFLDSRSRQLQCERELQESSLEACRRVVDQQLVGQLPWSTGLQMQCCQQLRDVSPECRPVALSQVVRQYEQQTEVPSKGGSFYPGGTAPPL<br>QQGGWWGTSVKWYYPDQTSSQQSWQGQQGYHQSVTSSQQPGQGQQGSYPGSTFPQQPGQGQQPGQRQPWSYPSATFPQQPGQGQGQQGYYPGATSLLQPGQGQQGPYQSATSPQQPGQGQ<br>GQQEPYPIATSPHQPGQWQQPGQGQQGYYPSVTSPQQSGQGQQGYPSTTSPQQSGQGQQLGQGQQPGQGQQGYPSATFPQQPGQWQQGSYPSTTSPQQSGQGQQGYNPSGTSTQQPGQVQ<br>QLGQGQQGYYPIATSPQQPGQGQQLGQGQQPGHGQQLVQGQQQGQGQQGHYPSMTSPHQTGQGQKGYYPSAISPQQSGQGQQGYQPSGASSQGSVQGACQHSTSSPQQQAQGCQASSPKQ<br>GLGSLYYPSGAYTQQKPGQGYNPGGTSPLHQQGGGFGGGLTTEQPQGGKQPFHCQQTTVSPHQGQQTTVSPHQGQQTTVSPHQGQQTTVSPHQGQQTTVSPHQGQQTTVSPHQGQQTTVS<br>PHPGQQTTVSPHQGQQTTVSPHPGQQTTVSPHQGQQTTVSPHQGQQTTVSPHQGQQTTVSPHQGQQTTVSPHQGQQTTVSPHQGQQPGEQPCGFPGQQTTVSLHHGQQSNELYYGSPYHV<br>SVEQPSASLKVAKAQQLAAQLPAMCRLEGGGGLLASQ

No.	BIOPEP ID	Protein name	Length (aa)	Amino Acid Sequence
30	1418	Omega-gliadin storage protein, wheat *(Triticum aestivum)*	280	MKTFLIFVLLAMAMKIATAARELNPSNKELQSPQQSFSYQQQPFPQQPYPQQPYPSQQPYPSQQPFPTPQQQFPEQSQQPFTQPQQPTPIQPQQPFPQQPQQPQQPFPQPQQPFPWQPQQPFPQTQQSFPLQPQQPFPQQPQQPFPQPQLPFPQQSEQIIPQQLQQPFPLQPQQPFPQQPQQPFPQPQQPIPVQPQQSFPQQSQQSQQPFAQPQQLFPELQQPIPQQPQQPFPLQPQQPFPQQPQQPFPQQPQQSFPQQPQQPYPQQQPYGSSLTSIGGQ
31	1402	Gamma-gliadin, wheat *(Triticum aestivum)*	337	MKTLLILTIIAVALTTTTANIQVDPSGQVQWPQQQQPFPQPQQPFSQQPQQIFPQPQQTFPHQPQQAFPQPQQTFPHQPQQQFPQPQQPQQPFPQQPQQQFPQPQQPQQPFPQQPQQQFPQPQQPQQPFPQPQQPQLPFPQQPQQPFPQPQQPQQPFPQLQQPQQPLPQPQQPQQPFPQQQQPLIQPYLQQQMNPCKNYLLQQCNPVSLVSSLVSMILPRSDCKVMRQQCCQQLARIPQQLQCAAIHGIVHSIIMQQEQQQQQQQQQQQQQQGIQIMRPLFQLVQGQGIIQPQQPAQLEVIRSLVLGTLPTMCNVFVPPECSTTKAPFASIVADIGGQ
32	1212	Lactoferrin, bovine *(Bos taurus)*	689	APRKNVRWCTISQPEWFKCRRWQWRMKKLGAPSITCVRRAFALECIRAIAEKKADAVTLDGGMVFEAGRDPYKLRPVAAEIYGTKESPQTHYYAVAVVKKGSNFQLDQLQGRKSCHTGLGRSAGWIIPMGILRPYLSWTESLEPLQGAVAKFFSASCVPCIDRQAYPNLCQLCKGEGENQCACSSREPYFGYSGAFKCLQDGAGDVAFVKETTVFENLPEKADRDQYELLCLNNSRAPVDAFKECHLAQVPSHAVVARSVDGKEDLIWKLLSKAQEKFGKNKSRSFQLFGSPPGQRDLLFKDSALGFLRIPSKVDSALYLGSRYLTTLKNLRETAEEVKARYTRVVWCAVGPEEQKKCQQWSQQSGQNVTCATASTTDDCIVLVLKGEADALNLDGGYIYTAGKCGLVPVLAENRKSSKHSSLDCVLRPTEGYLAVAVVKKANEGLTWNSLKDKKSCHTAVDRTAGWNIPMGLIVNQTGSCAFDEFFSQSCAPGADPKSRLCALCAGDDQGLDKCVPNSKEKYYGYTGAFRCLAEDVGDVAFVKNDTVWENTNGESTADWAKNLNREDFRLLCLDGTRKPVTEAQSCHLAVAPNHAVVSRSDRAAHVKQVLLHQQALFGKNGKNCPDKFCLFKSETKNLLFNDNTECLAKLGGRPTYEEYLGTEYVTAIANLKKCSTSPLLEACAFLTR
33	1213	Lactoferrin caprine*(lactotransferrin)*, (Capra hircus)	689	APRKNVRWCAISLPEWSKCYQWQRRMRKLGAPSITCIRRTSALECIRAIAGKNADAVTLDSGMVFEAGLDPYKLRPVAAEIYGTEKSPQTHYYAVAVVKKGSNFQLDQLQGQKSCHMGLGRSAGWNIPVGILRPFLSWTESAEPLQGAVARFFSASCVPCVDGKAYPNLCQLCKGVGENKCACSSQEPYFGYSGAFKCLQDGAGDVAFVKETTVFENLPEKADRDQYELLCLNNTRAPVDAFKECHLAQVPSHAVVARSVDGKENLIWELLRKAQEKFGKNKSQSFQLFGSPEGRRDLLFKDSALGFVRIPSKVDSALYLGSRYLTALKNLRETAEELKARCTRVVWCAVGPEEQSKCQQWSEQSGQNVTCATASTTDDCIALVLKGEADALSLDGGYIYTAGKCGLVPVMAENRKSSKYSSLDCVLRPTEGYLAVAVVKKANEGLTWNSLKGKKSCHTAVDRTAGWNIPMGLIANQTGSCAFDEFFSQSCAPGADPKSSLCALCAGDDQGLDKCVPNSKEKYYGYTGAFRCLAEDVGDVAFVKNDTVWENTNGESSADWAKNLNREDFRLLCLDGTTKPVTEAQSCYLAVAPNHAVVSRSDRAAHVEQVLLHQQALFGKNGKNCPDQFCLFKSETKNLLFNDNTECLAKLGGRPTYEKYLGTEYVTAIANLKKCSTSPLLEACAFLTR
34	1665	Lactoferrin, sheep, *(Ovis aries)*	708	MKLFVPALLSLGALGLCLAAPRKNVRWCAISPPEGSKCYQWQRRMRKLGAPSITCVRRTSALECIRAIAGKKADAVTLDSGMVFEAGLDPYKLRPVAAEIYGTEKSPQTHYYAVAVVKKGSNFQLDQLQGQKSCHMGLGRSAGWNIPMGILRPFLSWTESAEPLQGAVARFFSASCVPCVDGKAYPNLCQLCKGVGENKCACSSQEPYFGYSGAFKCLQDGAGDVAFVKETTVFENLPEKADRDQYELLCLNNTRAPVDAFKECHLAQVPSHAVVARSVDGKENLIWELLRKAQEKFGKNKSQRFQLFGSPQGQKDLLFKDSALGFVRIPSKVDSALYLGSRYLTALKNLRETAEEVKARCTPVVWCAVGPQEHSKCQQWSEQSGQNVTCAMASTTDECIALVLKGEADALSLDGGYIYTAGKCGLVPVMAENRESSKYSSLDCVLRPTEGYLAVAVVKKANEGLTWNSLKGKKSCHTAVDRTAGWNIPMGLIANQTGSCAFDEFFSQSCAPGADPKSSLCALCAGDDQGLNKCVPNSKEKYYGYTGAFRCLAEDVGDVAFVKNDTVWENTNGESSADWAKNLNREDFRLLCLDGTTKPVTEAQSCYLAVAPNHAVVSRSDRAAHVEQVLLHQQALFGKNGKNCPDQFCLFKSETKNLLFNDNTECLAKLGGRPTYEKYLGTEYVTAIANLKKCSTSPLLEACAFLTR
35	1397	Gamma-gliadin, wheat *(Triticum aestivum)*	327	MKTLLILTILAMAITIGTANIQVDPSGQVQWLQQQLVPQLQQPLSQQPQQTFPQPQQTFPHQPQQQVPQPQQPQQPFLQPQQPFPQQPQQPFPQTQQPQQPFPQQPQQPFPQTQQPQQPFPQQPQQPFPQTQQPQQPFPQLQQPQQPFPQPQQQLPQPQQPQQSFPQQQRPFIQPSLQQQLNPCKNILLQQCKPASLVSSLWSIIWPQSDCQVMRQQCCQQLAQIPQQLQCAAIHSVVHSIIMQQQQQQQQQQGMHIFLPLSQQQQVGQGSLVQGQGIIQPQQPAQLEAIRSLVLQTLPSMCNVYVPPECSIMRAPFASIVAGIGGQ
36	1749	Ovotransferrin *(conalbumin)*, precursor, chicken (Gallus gallus)	705	MKLILCTVLSLGIAAVCFAAPPKSVIRWCTISSPEEKKCNNLRDLTQQERISLTCVQKATYLDCIKAIANNEADAISLDGGQAFEAGLAPYKLKPIAAEVYEHTEGSTTSYYAVAVVKKGTEFTVNDLQGKTSCHTGLGRSAGWNIPIGTLLHRGAIEWEGIESGSVEQAVAKFFSASCVPGATIEQKLCRQCKGDPKTKCARNAPYSGYSGAFHCLKDGKGDVAFVKHTTVNENAPDQKDEYELLCLDGSRQPVDNYKTCNWARVAAHAVVARDDNKVEDIWSFLSKAQSDFGVDTKSDFHLFGPPGKKDPVLKDLLFKDSAIMLKRVPSLMDSQLYLGFEYYSAIQSMRKDQLTPSPRENRIQWCAVGKDEKSKCDRWSVVSNGDVECTVVDETKDCIIKIMKGEADAVALDGGLVYTAGVCGLVPVMAERYDDESQCSKTDERPASYFAVAVARKDSNVNWNNLKGKKSCHTAVGRTAGWVIPMGLIHNRTGTCNFDEYFSEGCAPGSPPNSRLCQLCQGSGGIPPEKCVASSHEKYFGYTGALRCLVEKGDVAFIQHSTVEENTGGKNKADWAKNLQMDDFELLCTDGRRANVMDYRECNLAEVPTHAVVVRPEKANKIRDLLERQEKRFGVNGSEKSKFMMFESQNKDLLFKDLTKCLFKVREGTTYKEFLGDKFYTVISSLKTCNPSDILQMCSFLEGK
37	1729	Serum albumin, precursor, bovine *(Bos taurus)*	607	MKWVTFISLLLLFSSAYSRGVFRRDTHKSEIAHRFKDLGEEHFKGLVLIAFSQYLQQCPFDEHVKLVNELTEFAKTCVADESHAGCEKSLHTLFGDELCKVASLRETYGDMADCCEKQEPERNECFLSHKDDSPDLPKLKPDPNTLCDEFKADEKKFWGKYLYEIARRHPYFYAPELLYYANKYNGVFQECCQAEDKGACLLPKIETMREKVLASSARQRLRCASIQKFGERALKAWSVARLSQKFPKAEFVEVTKLVTDLTKVHKECCHGDLLECADDRADLAKYICDNQDTISSKLKECCDKPLLEKSHCIAEVEKDAIPENLPPLTADFAEDKDVCKNYQEAKDAFLGSFLYEYSRRHPEYAVSVLLRLAKEYEATLEECCAKDDPHACYSTVFDKLKHLVDEPQNLIKQNCDQFEKLGEYGFQNALIVRYTRKVPQVSTPTLVEVSRSLGKVGTRCCTKPESERMPCTEDYLSLILNRLCVLHEKTPVSEKVTKCCTESLVNRRPCFSALTPDETYVPKAFDEKLFTFHADICTLPDTEKQIKKQTALVELLKHKPKATEEQLKTVMENFVAFVDKCCAADDKEACFAVEGPKLVVSTQTALA
38	1603	B1-hordein, precursor, barley *(Hordeum vulgare)*	293	MKTFLIFALLAIAATSTIAQQQPFPQQPIPQQPQPYPQQPQPYPQQPFPPQQPFPQQPVPQQPQPYPQQPFPPQQPFPQQPPFWQQKPFPQQPPFGLQQPILSQQQPCTPQQTPLPQGQLYQTLLQLQIQYVHPSILQQLNPCKVFLQQQCSPVPVPQRIARSQMLQQSSCHVLQQQCCQQLPQIPEQFRHEAIRAIVYSIFLQEQPQQLVEGVSQPQQQLWPQQVGQCSFQQPQPQQVGQQQQVPQSAFLQPHQIAQLEATTSIALRTLPMMCSVNVPLYRILRGVGPSVGV
39	1782	Glycinin, peanut *(Arachis hypogaea)*	530	MAKLLELSFCFCFLVLGASSISFRQQPEENACQFQRLNAQRPDNRIESEGGYIETWNPNNQEFECAGVALSRLVLRRNALRRPFYSNAPQEIFIQQGRGYFGLIFPGCPSTYEEPAQQGRRYQSQRPPRRLQEEDQSQQQQDSHQKVHRFNEGDLIAVPTGVAFWLYNDHDTDVVAVSLTDTNNNDNQLDQFPRRFNLAGNHEQEFLRYQQQSRQSRRRSLPYSPYSPHSRPRREEREFRPRGQHSRRERAGQEEEDEGGNIFSGFTPEFLEQAFQVDDRQIVQNLWGENESEEEGAIVTVRGGLRILSPDGTRGADEEEEYDEDQYEYHEQDGRRGRGSRGGGNGIEETICTACVKKNIGGNRSPHIYDPQRWFTQNCHDLNLLILRWLGLSAEYGNLYRNALFVPHYNTNAHSIIYALRGRAHVQVVDSNGNRVYDEELQEGHVLVVPQNFAVAGKSQSENFEYVAFKTDSRPSIANFAGENSFIDNLPEEVVANSYGLPREQARQLKNNNPFKFFVPPFQQSPRAVA
40	1463	11S globulin, precursor, oat *(Avena sativa)*	551	MATTSFPSMLFYFCIFLLFHGSMAQLFGQSSTPWQSSRQGGLRGCRFDRLQAFEPLRQVRSQAGITEYFDEQNEQFRCTGVSVIRRVIEPQGLVLPQYHNAPALVYILQGRGFTGLTFPGCPATFQQQFQPFDQSQFAQGQSQSQTIKDEHQRVQRFKQGDVVALPAGIVHWCYNDGDAPIVAIYVFDVNNNANQLEPRQKKFLLAGNNKFLLAGNNANQLEPRQKEFLLAGNNKREQQSGNNIFSGLSVQLLSEALGISQQAAQGSKSNDQRGRVIRVSQGLQFLKPIVSQQVPVEQQVYQPIQTQDVQATQYQVGQSTQYQVGKSTPYQGGQSSQYQAGQSWDQSFNGLEENFCSLEARKNIENPQHADTYNPRAGRITRLNSKNFPILNIVQMSATRVNLYQNAILSPFWNINAHSVIYMIQGHARVQVVNNNGQTVFSDILHRGQLLIVPQHFVVLKNAEREGCQYISFKTNPNSMVSHIAGKTSILRALPIDVLANAYRISRQEARNLKNNRGEEFGAFTPKLTQTGFQSYQDIEEASSSAVRASE
41	1535	Glutelin type II, precursor, rice *(Oryza sativa)*	499	MASINRPIVFFTVCLFLLCDGSLAQQLLGQSTSQWQSSRRGSPRGCRFDRLQAFEPIRSVRSQAGTTEFFDVSNELFQCTGVSVVRRVIEPRGLLLPHYTNGASLVYIIQGRGITGPTFPGCPETYQQQFQQSGQAQLTESQSQSHKFKDEHQKIHRFRQGDVIALPAGVAHWCYNDGEVPVVAIYVTDINNGANQLDPRQRDFLLAGNKRNPQAYRREVEEWSQNIFSGFSTELLSEAFGISNQVARQLQCQNDQRGEIVRVERGLSLLQPYASLQEQEQGQMQSREHYQEGGYQQSQYGSGCPNGLDETFCTMRVRQNIDNPNRADTYNPRAGRVTNLNSQNFPILNLVQMSAVKVNLYQNALLSPFWNINAHSIVYITQGRAQVQVVNNNGKTVFNGELRRGQLLIVPQHYVVVKKAQREGCAYIAFKTNPNSMVSHIAGKSSIFRALPTDVLANAYRISREEAQRLKHNRGDEFGAFTPLQYKSYQDVYNVAESS
42	1538	Glutelin, precursor, rice *(Oryza sativa)*	500	MATIAFSRLSIYFCVLLLCHGSMAQLFGPNVNPWHNPRQGGFRECRFDRLQAFEPLRRVRSEAGVTEYFDEKNEQFQCTGTFVIRRVIEPQGLLVPRYSNTPGMVYIIQGRGSMGLTFPGCPATYQQQFQQFLPEGQSQSQKFRDEHQKIHQFRQGDIVALPAGVAHWFYNEGDAPVVALYVFDLNNNANQLEPRQKEFLLAGNNNREQQMYGRSIEQHSGQNIFSGFNNELLSEALGVNALVAKRLQGQNDQRGEIIRVKNGLKLLRPAFAQQQEQAQQQEQAQAQYQVQYSEEQQPSTRCNGLDENFCTIKARLNIENPSHADTYNPRAGRITRLNSQKFPILNLVQLSATRVNLYQNAILSPFWNVNAHSLVYIVQGHARVQVVSNLGKTVFNGVLRPGQLLIIPQHYVVLKKAEHEGCQYISFKTNANSMVSHLAGKNSIFRAMPVDVIANAYRISREQARSLKNNRGEELGAFTP

No.	BIOPEP ID	Protein name	Length (aa)	Amino Acid Sequence
				RYQQQTYPGFSNESENEALE
43	1161	11S globulin beta subunit pumpkin *(Cucurbita species)*	480	MARSSLFTFLCLAVFINGCLSQIEQQSPWEFQGSEVWQQHRYQSPRACRLENLRAQDPVRRAEAEAIFTEVWDQDNDEFQCAGVNMIRHTIRPKGLLLPGFSNAPKLIFVAQGFGIRGIAIPGCAETYQTDLRRSQSAGSAFKDQHQKIRPFREGDLLVVPAGVSHWMYNRGQSDLVLIVFADTRNVANQIDPYLRKFYLAGRPEQVERGVEEWERSSRKGSSGEKSGNIFSGFADEFLEEAFQIDGGLVRKLKGEDDERDRIVQVDEDFEVLLPEKDEEERSRGRYIESESESENGLEETICTLRLKQNIGRSVRADVFNPRGGRISTANYHTLPILRQVRLSAERGVLYSNAMVAPHYTVNSHSVMYATRGNARVQVVDNFGQSVFDGEVREGQVLMIPQNFVVIKRASDRGFEWIAFKTNDNAITNLLAGRVSQMRMLPLGVLSNMYRISREEAQRLKYGQQEMRVLSPGRSQGRRE
44	1602	Gamma-hordein 3, barley *(Hordeum vulgare)*	289	ITTTTMQFNPSGLELERPQQLFPQWQPLPQQPPFLQQEPEQPYPQQQPLPQQQPFPQQPQLPHQHQFPQQLPQQQFPQQMPLQPQQQFPQQMPLQPQQQPQFPQQKPFGQYQQPLTQQPYPQQQPLAQQQPSIEEQHQLNLCKEFLLQQCTLDEKVPLLQSVISFLRPHISQQNSCQLKRQQCCQQLANINEQSRCPAIQTIVHAIVMQQQVQQQVGHGFVQSQLQQLGQGMPIQLQQQPGQAFVLPQQQAQFKVVGSLVIQTLPMLCNVHVPPYCSPFGSMATGSGGQ
45	1304	Alpha-gliadin, wheat *(Triticum aestivum)*	287	MKTFLILVLLAIVATTAPTAVRFPVPQLQPQNPSQQLPQEQVPLVQQQQFLGQQQPFPPQQPYPQPQFPSQLPYLQLQPFPQPQLPYSQPQPFRPQQPYPQPQPQYSQPQQPISQQQQQQQQQQQQQQQQQILQQILQQQLIPCMDVVLQQHNKAHGRSQVLQQSTYQLLRELCCQHLWQIPEQSQCQAIHNVVHAIILHQQQKQQQQQPSSQVSFQQPLQQYPLGQGSFRPSQQNPQTQGSVQPQQLPQFEEIRNLALQTLPSMCNVYIPPYCTIAPFGIFGTN
46	1593	Glutelin, rice *(Oryza sativa)*	510	MASMSTILPLCLGLLLFFQVSMAQFSFGGSPLQSPRGFRGDQDSRHQCRFEHLTALEATHQQRSEAGFTEYYNIEARNEFRCAGVSVRRLVVESKGLVLPMYANAHKLVYIVQGRGVFGMALPGCPETFQSVRSPFEQEVATAGEAQSSIQKMRDEHQQLHQFHQGDVIAVPAGVAHWLYNNGDSPVVAFTVIDTSNNANQLDPKRREFFLAGKPRSSWQQQSYSYQTEQLSRNQNIFAGFSPDLLSEALSVSKQTVLRLQGLSDPRGAIIRVENGLQALQPSLQVEPVKEEQTQAYLPTKQLQPTWLRSGGACGQQNVLDEIMCAFKLRKNIDNPQSSDIFNPHGGRITRANSQNFPILNIIQMSATRIVLQNNALLTPHWTVNAHTVMYVTAGQGHIQVVDHRGRSVFDGELHQQQILLIPQNFAVVVKARREGFAWVSFKTNHNAVDSQIAGKASILRALPVDVVANAYRLSREDSRHVKFNRGDEMAVFAPRRGPQQYAEWQINEK
47	1156	Legumin 1, mayze *(Zea mayze)*	483	MAAAIVLSGQVRPLPSSLPLSLLLLLLLCCSGTSWGWSTSRGGAARECGFDGKLEALEPRHKVQSEAGSVQYFSRFNEADRELTCAGIFAVRVVVDAMGLLLPRYSNVHSLVYIVQGRGIIGFSFPGCQEETQQQQYGYGYGYGHHHHQHDHHKIHRFEQGDVVAMPAGAQHWLYNDGDAPLVAVYVFDENNNINQLEPSMRKFLLAGGFSKGQPHFAENIFKGIDARFLSEALGVSMHVAEKLQSRRDQRGEIVRVEPEHGFHQLNPSPSSSSFSFPSSQVQYQTCQRDVDRHNVCAMEVRHSVERLDQADVYSPGAGRITRLTSHKFPVLNLVQMSAVRVDLYQDAIMSPFWNFNAHSAMYGIRGSARVQVASDNGTTVFDDVLRAGQLLIVPQGYLVATKAQGEGFQYIAFETNPDTMVSHVAGKNSVLSDLPAAVIASSYAISMEEAAELKNGRKHELAVLTPAGSGSYQQGQAGSAQQ
48	1469	13S globulin seed storage protein 2, precursor, common buckwheat *(Fagopyrum esculentum)*	504	MSTKLILSFSLCLMVLSCSAQLWPWQKGQGSRPHHGRQQHQFQHQCDIQRLTASEPSRRVRSEAGVTEIWDHDTPEFRCTGFVAVRVVIQPGGLLLPSYSNAPYITFVEQGRGVQGVVIPGCPETFQSDSEFEYPQSQRGRHSRQSESEEESSRGDQHQKIFRIREGDVIPSPAGVVQWTHNDGNDDLISVTLLDANSYHKQLDENVRSFFLAGQSQRETREEGSDRQSRESDDDEALLGANILSGFQDEILHELFRDVDRETISKLRGENDQRGFIVQAQDLKLRVPQDFEEEYERERGDRRRGQGGSGRSNGVEQGFCNLKFRRNFNTPTNTYVFNPRAGRINTVNSNSLPILEFLQLSAQHVVLYKNAIIGPRWNLNAHSALYVTRGEGRVQVVGDEGKSVFDDKVQRGQILVVPQGFAVVLKAGREGLEWVELKNSGNAITSPIGGRTSVLRAIPVEVLANSYDISTKEAYKLKNGRQEVEVFRPFQSRDEKERERFSIV
49	1240	Precursor of G4 glycinin, soybean *(Glycine max)*	562	MGKPFTLSLSSLCLLLLSSACFAISSSKLNECQLNNLNALEPDHRVESEGGLIQTWNSQHPELKCAGVTVSKLTLNRNGLHSPSYSPYPRMIIIAQGKGALGVAIPGCPETFEEPQEQSNRRGSRSQKQQLQDSHQKIRHFNEGDVLVIPPSVPYWTYNTGDEPVVAISLLDTSNFNNQLDQTPRVFYLAGNPDIEYPETMQQQQQQKSHGGRKQGQHQQEEEEEGGSVLSGFSKHFLAQSFNTNEDIAEKLESPDDERKQIVTVEGGLSVISPKWQEQQDEDEDEDEDDEDEQIPSHPPRRPSHGKREQDEDEDEDEDKPRPSRPSQGKRNKTGQDEDEDEDEDQPRKSREWRSKKTQPRRPRQEEPRERGCETRNGVEENICTLKLHENIARPSRADFYNPKAGRISTLNSLTLPALRQFQLSAQYVVLYKNGIYSPHWNLNANSVIYVTRGQGKVRVVNCQGNAVFDGELRRGQLLVVPQNFVVAEQAGEQGFEYIVFKTHHNAVTSYLKDVFRAIPSEVLAHSYNLRQSQVSELKYEGNWGPLVNPESQQGSPRVKVA
50	1787	7 S globulin, oriental sesame *(Sesamum indicum)*	585	MSCGGRLCLVLFALLLASAVVASESKDPELKQCKHQCKAQQQISKEQKEACIQACKEYIRQKHQGEHGRGGGDILEEEVWNRKSPIERLRECSRGCEQQHGEQREECLRRCQEEYQREKGRQDDDNPTDPEKQYQQCRLQCRRQGEGGGFSREHCERRREEKYREQQGREGGRGEMYEGREREEEQEEQGRGRIPYVFEDQHFITGFRTQHGRMRVLQKFTDRSELLRGIENYRVAILEAEPQTFIVPNHWDAESVVFVAKGRGTISLVRQDRRESLNIKQGDILKINAGTTAYLINRDNNERLVLAKLLQPVSTPGEFELFFGAGGENPESFFKSFSDEILEAAFNTRRDRLQRIFGQQRQGVIVKASEEQVRAMSRHEEGGIWPFGGESKGTINIYQQRPTHSNQYGQLHEVDASQYRQLRDLDLTVSLANITQGAMTAPHYNSKATKIALVVDGEGYFEMACPHMSRSRGSYQGETRGRPSYQRVASRLTRGTVVIIPAGHPFVAVASSNQNLQVLCFEVNANNNEKFPLAGRRNVMNQLEREAKELAFGMPAREVEEVSRSQQEEFFFKGPRQQQQGRADA
51	1163	11S globulin seed storage protein G3 precursor , common sunflower *(Helianthus annuus)*	493	MASKATLLLAFTLLFATCIARHQQRQQQQNQCQLQNIEALEPIEVIQAEAGVTEIWDAYDQQFQCAWSILFDTGFNLVAFSCLPTSTPLFWPSSREGVILPGCRRTYEYSQEQQFSGEGGRRGGGEGTFRTVIRKLENLKEGDVVAIPTGTAHWLHNDGNTELVVVFLDTQNHENQLDENQRRFFLAGNPQAQAQSQQQQQRQPRQQSPQRQRQRQRQGQGQNAGNIFNGFTPELIAQSFNVDQETAQKLQGQNDQRGHIVNVGQDLQIVRPPQDRRSPRQQQEQATSPRQQQEQQQGRRGGWSNGVEETICSMKFKVNIDNPSQADFVNPQAGSIANLNSFKFPILEHLRLSVERGELRPNAIQSPHWTINAHNLLYVTEGALRVQIVDNQGNSVFDNELREGQVVVIPQNFAVIKRANEQGSRWVSFKTNDNAMIANLAGRVSASAASPLTLWANRYQLSREEAQQLKFSQRETVLFAPSFSRGQGIRASR
52	1502	Legumin A , precursor, garden pea *(Pisum sativum)*	517	MAKLLALSLSFCFLLLGGCFALREQPQQNECQLERLDALEPDNRIESEGGLIETWNPNNKQFRCAGVALSRATLQRNALRRPYYSNAPQEIFIQQGNGYFGMVFPGCPETFEEPQESEQGEGRRYRDRHQKVNRFREGDIIAVPTGIVFWMYNDQDTPVIAVSLTDIRSSNNQLDQMPRRFYLAGNHEQEFLQYQHQQGGKQEQENEGNNIFSGFKRDYLEDAFNVNRHIVDRLQGRNEDEEKGAIVKVKGGLSIISPPEKQARHQRGSRQEEDEDEDEEKQPRHQRGSRQEEEEDEDEERQPRHQRRRGEEEEEDKKERGGSQKGKSRRQGDNGLEETVCTAKLRLNIGPSSSPDIYNPEAGRIKTVTSLDLPVLRWLKLSAEHGSLHKNAMFVPHYNLNANSIIYALKGRARLQVVNCNGNTVFDGELEAGRALTVPQNYAVAAKSLSDRFSYVAFKTNDRAGIARLAGTSSVINNLPLDVVAATFNLQRNEARQLKSNNPFKFLVPARESENRASA
53	1790	11S globulin seed storage protein 2, precursor, oriental sesame *(Sesamum indicum)*	459	MVAFKFLLALSLSLLVSAAIAQTREPRLTQGQQCRFQRISGAQPSLRIQSEGGTTELWDERQEQFQCAGIVAMRSTIRPNGLSLPNYHPSPRLVYIERGQGLISIMVPGCAETYQVHRSQRTMERTEASEQQDRGSVRDLHQKVHRLRQGDIVAIPSGAAHWCYNDGSEDLVAVSINDVNHLSNQLDQKFRAFYLAGGVPRSGEQEQQARQTFHNIFRAFDAELLSEAFNVPQETIRRMQSEEEERGLIVMARERMTFVRPDEEEGEQEHRGRQLDNGLEETFCTMKFRTNVESRREADIFSRQAGRVHVVDRNKLPILKYMDLSAEKGNLYSNALVSPDWSMTGHTIVYVTRGDAQVQVVDHNGQALMNDRVNQGEMFVVPQYYTSTARAGNNGFEWVAFKTTGSPMRSPLAGYTSVIRAMPLQVITNSYQISPNQAQALKMNRGSQSFLLSPGGRRS
54	1100	Beta-casein, gen. Var. B, bovine *(bos taurus)*	209	RELEELNVPGEIVESLSSSEESITRINKKIEKFQSEEQQQTEDELQDKIHPFAQTQSLVYPFPGPIPNSLPQNIPPLTQTPVVVPPFLQPEVMGVSKVKEAMAPKHKEMPFPKYPVEPFTERQSLTLTDVENLHLPLPLLQSWMHQPHQPLPPTVMFPPQSVLSLSQSKVLPVPQKAVPYPQRDMPIQAFLLYQEPVLGPVRGPFPIIV
55	1098	Beta-casein gen. Var. A2 *(bos taurus)*	209	RELEELNVPGEIVESLSSSEESITRINKKIEKFQSEEQQQTEDELQDKIHPFAQTQSLVYPFPGPIPNSLPQNIPPLTQTPVVVPPFLQPEVMGVSKVKEAMAPKHKEMPFPKYPVQPFTESQSLTLTDVENLHLPPLLLQSWMHQPHQPLPPTVMFPPQSVLSLSQSKVLPVPEKAVPYPQRDMPIQAFLLYQQPVLGPVRGPFPIIV
56	1139	Legumin A2 , garden pea *(Pisum sativum)*	498	LREQPEQNECQLERLNALEPDNRIESEGGLIETWNPNNKQFRCAGVALSRATLQHNALRRPYYSNAPQEIFIQQGNGYFGMVFPGCPETFEEPQESEQGEGRRYRDRHQKVNRFREGDIIAVPTGIVFWMYNDQDTPVIAVSLTDIRSSNNQLDQMPRRFYLAGNHEQEFLRYQHQQGGKQEQENEGNNIFSGFKRDFLEDAFNVNRHIVDRLQGRNEDEEKGAIVKVKGGLSIISPPEKQARHQRGSRQEEDEDEDEERQPRHQRGSRQEEEEDEDEERQPRHQRRRGEEEEEDKKERRGSQKGKSRRQGDNGLEETVCTAKLRLNIGPSSSPDIYNPEAGRIKTVTSLDLPVLRWLKLSAEHGSLHKNAMFVPHYNLNANSIIYALKGRARLQVVNCNGNTVFDGELEAGRALTVPQNYAVAAKSLSDRFSYVAFKTNDRAGIARLAGTSSVINNLPLDVVAATFNLQRNEARQLKSNNPFKFLVPARQSENRASA

No.	BIOPEP ID	Protein name	Length (aa)	Amino Acid Sequence
57	1097	Beta-casein gen. Var. A1 *(bos taurus)*	209	RELEELNVPGEIVESLSSSEESITRINKKIEKFQSEEQQQTEDELQDKIHPFAQTQSLVYPFPGPIHNSLPQNIPPLTQTPVVVPPFLQPEVMGVSKVKEAMAPKHKEMPFPKYPVQPFT<br>ESQSLTLTDVENLHLPPLLLQSWMHQPHQPLPPTVMFPPQSVLSLSQSKVLPVPEKAVPYPQRDMPIQAFLLYQQPVLGPVRGPFPIIV
58	1099	Beta-casein gen. Var. A3 *(bos taurus)*	209	RELEELNVPGEIVESLSSSEESITRINKKIEKFQSEEQQQTEDELQDKIHPFAQTQSLVYPFPGPIHNSLPQNIPPLTQTPVVVPPFLQPEVMGVSKVKEAMAPKQKEMPFPKYPVQPFT<br>ESQSLTLTDVENLHLPPLLLQSWMHQPHQPLPPTVMFPPQSVLSLSQSKVLPVPEKAVPYPQRDMPIQAFLLYQQPVLGPVRGPFPIIV
59	1238	Vicilin, garden pea *(Pisum sativum)*	432	SRSDPQNPFIFKSNKFQTLFENENGHIRLLQKFDQRSKIFENLQNYRLLEYKSKPHTIFLPQHTDADYILVVLSGKAILTVLKPDDRNSFNLERGDTIKLPAGTIAYLVNRDDNEELRVL<br>DLAIPVNRPGQLQSFLLSGNQNQQNYLSGFSKNILEASFNTDYEEIEKVLLEEHEKETQHRRSLKDKRQQSQEENVIVKLSRGQIEELSKNAKSTSKKSVSSESEPFNLRSRGPIYSNEF<br>GKFFEITPEKNPQLQDLDIFVNSVEIKEGSLLLPHYNSRAIVIVTVNEGKGDFELVGQRNENQQEQRKEDDEEEEQGEEEINKQVQNYKAKLSSGDVFVIPAGHPVAVKASSNLDLLGFG<br>INAENNQRNFLAGDEDNVISQIQRPVKELAFPGSAQEVDRILENQKQSHFADAQPQQRERGSRETRDRLSSV
60	1235	Cruciferin, 11S globulin, rape *(Brassica napus)*	486	RQSLGVPPQLGNACNLDNLDVLQPTETIKSEAGRVEYWDHNNPQIRCAGVSVSRVIIEQGGLYLPTFFSSPKISYVVQGMGISGRVVPGCAETFMDSQPMQGQQQGQPWQGQQGQQG<br>QQGQQGQQGQQGQQGQQGQQGQQGQQQQGFRDMHQKVEHVRHGDIIAITAGSSHWIYNTGDQPLVIICLLDIANYQNQLDRNPRTFRLAGNNPQGGSQQQQQQQNMLSGFDPQVLAQAL<br>KIDVRLAQELQNQQDSRGNIVRVKGPFQVVRPPLRQPYESEQWRHPRGPPQSPQDNGLEETICSMRTHENIDDPARADVYKPNLGRVTSVNSYTLPILQYIRLSATRGILQGNAMVLPKY<br>NMNANEILYCTQGQARIQVVNDNGQNVLDQQVQKGQLVVIPQGFAYVVQSHQNNFEWISFKTNANAMVSTLAGRTSALRALPLEVITNAFQISLEEARRIKFNTLETTLTRARGGQPQLI<br>EEIVEA
61	1117	Kappa-casein gen. Var. A, precursor, bovine *(bos taurus)*	190	MMKSFFLVVTILALTLPFLGAQEQNQEQPIRCEKDERFFSDKIAKYIPIQYVLSRYPSYGLNYYQQKPVALINNQFLPYPYYAKPAAVRSPAQILQWQVLSNTVPAKSCQAQPTTMARHP<br>HPHLSFMAIPPKKNQDKTEIPTINTIASGEPTSTPTTEAVESTVATLEDSPEVIESPPEINTVQVTSTAV
62	1109	Kappa-casein, goat *(Capra hircus)*	192	MMKSFFLVVTILALTLPFLGAQEQNQEQPICCEKDERFFDDKIAKYIPIQYVLSRYPSYGLNYYQQRPVALINNQFLPYPYYAKPVAVRSPAQTLQWQVLPNTVPAKSCQDQPTTLARHP<br>HPHLSFMAIPPKKDQDKTEVPAINTIASAEPTVHSTPTTEAIVNTVDNPEASSESIASASETNTAQVTSTEV
63	1748	Ovomucoid, precursor, chicken *(Gallus gallus)*	210	MAMAGVFVLFSFVLCGFLPDAAFGAEVDCSRFPNATDKEGKDVLVCNKDLRPICGTDGVTYTNDCLLCAYSIEFGTNISKEHDGECKETVPMNCSSYANTTSEDGKVMVLCNRAFNPVCG<br>TDGVTYDNECLLCAHKVEQGASVDKRHDGGCRKELAAVSVDCSEYPKPDCTAEDRPLCGSDNKTYGNKCNFCNAVVESNGTLTLSHFGKC
64	1092	Lysozyme C precursor, chicken *(Gallus gallus)*	147	MRSLLILVLCFLPLAALGKVFGRCELAAAMKRHGLDNYRGYSLGNWVCAAKFESNFNTQATNRNTDGSTDYGILQINSRWWCNDGRTPGSRNLCNIPCSALLSSDITASVNCAKKIVSDG<br>NGMNAWVAWRNRCKGTDVQAWIRGCRL
65	1741	Avidin precursor, chicken *(Gallus gallus)*	152	MVHATSPLLLLLLLSLALVAPGLSARKCSLTGKWTNDLGSNMTIGAVNSRGEFTGTYITAVTATSNEIKESPLHGTQNTINKRTQPTFGFTVNWKFSESTTVFTGQCFIDRNGKEVLKTM<br>WLLRSSVNDIGDDWKATRVGINIFTRLRTQKE
66	1854	Alpha-lactalbumin, gen. Var. A, bovine *(bos taurus)* with disulfide bonds	139	EQLTK{C}EVFQELKDLKGYGGVSLPEWV{C}TTFHTSGYDTQAIVQNNDSTEYGLFQINNKIW{C}KDDQNPHSSNI{C}NIS{C}DKFLDDDLTDDIM{C}VKKILDKVGINYWLAHK<br>AL{C}SEKLDQWL{C}EKL
67	1856	Alpha-lactalbumin, gen. Var. B, bovine *(bos taurus)* with disulfide bonds	138	EQLTK{C}EVFRELKDLKGYGGVSLPEWV{C}TTFHTSGYDTQAIVQNNDSTEYGLFQINNKIW{C}KDDQNPHSSNI{C}NIS{C}DKFLDDDLTDDIM{C}VKKILDKVGNYWLAHKA<br>L{C}SEKLDQWL{C}EKL
68	1272	Myoglobin *(Bos taurus)*	154	MGLSDGEWQLVLNAWGKVEADVAGHGQEVLIRLFTGHPETLEKFDKFKHLKTEAEMKASEDLKKHGNTVLTALGGILKKKGHHEAEVKHLAESHANKHKIPVKYLEFISDAIIHVLHAKH<br>PSDFGADAQAAMSKALELFRNDMAAQYKVLGFHG
69	1082	Alpha-lactalbumin, sheep *(Ovis aries)*	123	EQLTKCEAFQKLKDLKDYGGVSLPEWVCTAFHTSGYDTQAIVQNNDSTEYGLFQINNKIWCKDDQNPHSRNICNISCDKFLDDDLTDDIVCAKKILDKVGINYWLAHKALCSEKLDQWLC<br>EKL
70	1125	Myoglobin	135	VLHVWAKVEADVAGHGQFKSHPETLEKFDRFKKTEAEMKASEDLKKHGVTVLTALGAILKKKGHHEAELKPLAQSHATKHKIIKYLEFISEAIIHVLHSRHPGDFGADAQGAMNKALELF<br>RKDIAAKYKELGYQG
71	1762	Parvalbumin-beta 1, Atlantic salmon *(Salmo salar)*	109	MACAHLCKEADIKTALEACKAADTFSFKTFFHTIGFASKSADDVKKAFKVIDQDASGFIEVEELKLFLQNFCPKARELTDAETKAFLKAGDADGDGMIGIDEFAVLVKQ
72	1738	Parvalbumin-beta 1, cod *(Alaska pollack)*	109	MACAHLCKEADIKTALEACKAADTFSFKTFFHTIGFASKSADDVKKAFKVIDQDASGFIEVEELKLFLQNFCPKARELTDAETKAFLKAGDADGDGMIGIDEFAVLVKQ
73	1758	Parvalbumin, cyp c 1.01, common carp *(Cyprinus carpio)*	109	MAFAGILNDADITAALQGCQAADSFDYKSFFAKVGLSAKTPDDIKKAFAVIDQDKSGFIEEDELKLFLQNFSAGARALTDAETKAFLKAGDSDGDGKIGVDEFAALVKA
74	1851	Beta-lactoglobulin, gen. Var. A, bovine *(bos taurus)* with disulfide bonds	170	LIVTQTMKGLDIQKVAGTWYSLAMAASDISLLDAQSAPLRVYVEELKPTPEGDLEILLQKWENDE{C}AQKKIIAEKTKIPAVFKIDALNENKVLVLDTDYKKYLLF{C}MENSAEPEQS<br>LV{C}QCLVRTPEVDDEALEKFDKALKALPMHIRLSFNPTQLEEQ{C}HI
75	1852	Beta-lactoglobulin, gen. Var. B, bovine *(bos taurus)* with disulfide bonds	169	LIVTQTMKGLDIQKVAGTWYSLAMAASDISLLDAQSAPLRVYVEELKPTPEGDLEILLQKWENGE{C}AQKKIIAEKTKIPAVFKIDALNENKVLVLDTDYKKYLLF{C}MENSAPEQSL<br>A{C}QCLVRTPEVDDEALEKFDKALKALPMHIRLSFNPTQLEEQ{C}HI
76	1853	Beta-lactoglobulin, gen. Var. C, bovine *(bos taurus)* with disulfide bonds	170	LIVTQTMKGLDIQKVAGTWYSLAMAASDISLLDAQSAPLRVYVEELKPTPEGDLEILLHKWENGE{C}AQKKIIAEKTKIPAVFKIDALNENKVLVLDTDYKKYLLF{C}MENSAEPEQS<br>LA{C}QCLVRTPEVDDEALEKFDKALKALPMHIRLSFNPTQLEEQ{C}HI
77	1104	Beta-lactoglobulin precursor, goat *(Capra hircus)*	162	IIVTQTMKGLDIQKVAGTWYSLAMAASDISLLDAQSAPLRVYVEELKPTPEGNLEILLQKWENGECAQKKIIAEKTKIPAVFKIDALNENKVLVLDTDYKKYLLFCMENSAEPEQSLACQ<br>CLVRTPEVDKEALEKFDKALKALPMHIRLAFNPTQLEGQCHV
78	1105	Beta-lactoglobulin, sheep *(Ovis aries)*	162	IIVTQTMKGLDIQKVAGTWYSLAMAASDISLLDAQSAPLRVYVEELKPTPEGNLEILLQKWENGECAQKKIIAEKTKIPAVFKIDALNENKVLVLDTDYKKYLLFCMENSAEPEQSLACQ<br>CLVRTPEVDNEALEKFDKALKALPMHIRLAFNPTQLEGQCHV

No.	BIOPEP ID	Protein name	Length (aa)	Amino Acid Sequence
79	1739	Parvalbumin-beta 2, Atlantic salmon *(Salmo salar)*	108	MSFAGLNDADVAAALAACTAADSFNHKAFFAKVGLASKSSDDVKKAFYVIDQDKSGFIEEDELKLFLQNFSASARALTDAETKAFLADGDKDGDGMIGVDEFAAMIKG
80	1740	Parvalbumin-beta 1, Atlantic cod *(Gadus morhua)*	109	MAFAGILNDADITAALAACKAEGSFDHKAFFTKVGLAAKSPADIKKVFEIIDQDKSDFVEEDELKLFLQNFSAGARALSDAETKVFLKAGDSDGDGKIGVDEFGAMIKA
81	1759	Parvalbumin, cyp c 1.02, common carp *(Cyprinus carpio)*	109	MAFAGVLNDADITAALEACKAADSFNHKTFFAKVGLTSKSADDVKKAFAIIDQDKSGFIEEDELKLFLQNFKAGARALTDGETKTFLKAGDSDGDGKIGVDEFTALVKA
82	1761	Parvalbumin-beta, chub mackerel *(Scomber japonicus)*	109	MAFASVLKDAEVTAALDGCKAAGSFDHKKFFKACGLSGKSTDEVKKAFAIIDQDKSGFIEEEELKLFLQNFKAGARALSDAETKAFLKAGDSDGDGKIGIDEFAAMIKG
83	1128	Tropomyosin 1 alpha chain, chicken *(Gallus gallus)*	284	MDAIKKKMQMLKLDKENALDRAEQAEADKKAAEERSKQLEDELVALQKKLKGTEDELDKYSESLKDAQEKLELADKKATDAESEVASLNRRIQLVEEELDRAQERLATALQKLEEAEKAA<br>DESERGMKVIENRAQKDEEKMEIQEIQLKEAKHIAEEADRKYEEVARKLVIIEGDLERAEERAELSESKCAELEEELKTVTNNLKSLEAQAEKYSQKEDKYEEEIKVLTDKLKEAETRAE<br>FAERSVTKLEKSIDDLEDELYAQKLKYKAISEELDHALNDMTSI
84	1130	Tropomyosin beta chain, chicken *(Gallus gallus)*	284	MEAIKKKMQMLKLDKENAIDRAEQAEADKKQAEDRCKQLEEEQQGLQKKLKGTEDEVEKYSESVKEAQEKLEQAEKKATDAEAEVASLNRRIQLVEEELDRAQERLATALQKLEEAEKAA<br>DESERGMKVIENRAMKDEEKMELQEMQLKEAKHIAEEADRKYEEVARKLVVLEGELERSEERAEVAESKCGDLEEELKIVTNNLKSLEAQADKYSTKEDKYEEEIKLLGEKLKEAETRAE<br>FAERSVAKLEKTIDDLEDEVYAQKMKYKAISEELDNALNDITSL

Table S2. Summary of *in silico* enzymatic digestion of 84 commercially available food proteins using eight proteases. Cleavage specificity data were obtained from the BIOPEP-UWM database (https://biochemia.uwm.edu.pl/biopep-uwm/).

No.	Enzyme	EC Number	Cleavage Specificity	Peptides (≥ 2 aa)	Free Amino Acids	Total Fragments
1	Chymotrypsin A	EC 3.4.21.1	C-term: **F, H, L, M, N, W, Y**	5,174	1,762	6,936
2	Trypsin	EC 3.4.21.4	C-term: **K, R**	2,533	307	2,840
3	Pepsin (pH 1.3)	EC 3.4.23.1	C-term: **F, L**	3,169	552	3,721
4	Prolyl oligopeptidase	EC 3.4.21.26	C-term: **P**	2,208	97	2,305
5	Thermolysin	EC 3.4.24.27	N-term: **A, F, I, L, V, Y**	5,838	3,494	9,332
6	Papain	EC 3.4.22.2	C-term: **F, G, L, R, T**<br>N-term: **A, Q**	7,766	4,755	12,521
7	Stem bromelain	EC 3.4.22.32	C-term: **A, F, G, L, R, S, T, V**	6,898	6,774	13,672
8	Pepsin (pH > 2)	EC 3.4.23.1	C-term: **A, D, E, F, G, K, L, M, N, Q, T, Y**<br>N-term: **I, V**	6,600	14,039	20,639

C-term, cleavage at the C-terminal side of the specified amino acid residue; N-term, cleavage at the N-terminal side of the specified amino acid residue. Amino acids are represented by one-letter codes. Peptides refers to fragments with ≥ 2 amino acid residues; free amino acids refer to single residue fragments generated from digestion.

Table S3. Twenty-four protein subunit–enzyme combinations selected from 84 proteins × 8 enzymes (672 combinations) by P1' frequency tertile stratification for construction of the rule-development set.

No.	Protein subunit (BIOPEP name)	ID [a]	Enzyme	Total pep. [b]	P1' pep. [c]	P1' freq. (%)	Tertile group	Protein source [d]
1	Lactoferrin, bovine (*Bos taurus*)	1212	Prolyl oligopeptidase	30	29	96.7	High	Milk
2	kappa-casein gen. var. A, precursor, bovine (*Bos taurus*)	1117	Prolyl oligopeptidase	20	19	95.0	High	Milk
3	beta-lactoglobulin, gen. var. A, bovine (*Bos taurus*) with disulfide bonds	1851	Prolyl oligopeptidase	9	8	88.9	High	Milk
4	Myoglobin (*Bos taurus*)	1272	Prolyl oligopeptidase	4	3	75.0	High	Meat
5	alpha-lactalbumin, gen. var. A, bovine (*Bos taurus*) with disulfide bonds	1854	Prolyl oligopeptidase	3	2	66.7	High	Milk
6	Glutenin, low molecular weight (LMW), wheat (*Triticum aestivum*)	1329	Pepsin (pH 1.3)	41	22	53.7	High	Wheat
7	Gamma-gliadin, wheat (*Triticum aestivum*)	1319	Papain	82	36	43.9	High	Wheat
8	Lactoferrin, bovine (*Bos taurus*)	1212	Chymotrypsin A	125	47	37.6	High	Milk
9	alpha-lactalbumin, gen. var. A, bovine (*Bos taurus*) with disulfide bonds	1854	Chymotrypsin A	28	9	32.1	Medium	Milk
10	kappa-casein gen. var. A, precursor, bovine (*Bos taurus*)	1117	Pepsin (pH 1.3)	16	5	31.3	Medium	Milk
11	Glutenin, low molecular weight (LMW), wheat (*Triticum aestivum*)	1329	Pepsin (pH＞2)	85	26	30.6	Medium	Wheat
12	Gamma-gliadin, wheat (*Triticum aestivum*)	1319	Thermolysin	55	16	29.1	Medium	Wheat
13	Lactoferrin, bovine (*Bos taurus*)	1212	Pepsin (pH 1.3)	78	20	25.6	Medium	Milk
14	Glutenin, low molecular weight (LMW), wheat (*Triticum aestivum*)	1329	Stem bromelain	65	16	24.6	Medium	Wheat
15	beta-lactoglobulin, gen. var. A, bovine (*Bos taurus*) with disulfide bonds	1851	Chymotrypsin A	33	8	24.2	Medium	Milk
16	Myoglobin (*Bos taurus*)	1272	Chymotrypsin A	39	9	23.1	Medium	Meat
17	Myoglobin (*Bos taurus*)	1272	Papain	40	5	12.5	Low	Meat
18	Lactoferrin, bovine (*Bos taurus*)	1212	Papain	185	22	11.9	Low	Milk
19	beta-lactoglobulin, gen. var. A, bovine (*Bos taurus*) with disulfide bonds	1851	Pepsin (pH＞2)	36	4	11.1	Low	Milk
20	alpha-lactalbumin, gen. var. A, bovine (*Bos taurus*) with disulfide bonds	1854	Thermolysin	30	3	10.0	Low	Milk
21	Glutenin, low molecular weight (LMW), wheat (*Triticum aestivum*)	1329	Trypsin	11	1	9.1	Low	Wheat
22	Lactoferrin, bovine (*Bos taurus*)	1212	Thermolysin	144	12	8.3	Low	Milk
23	kappa-casein gen. var. A, precursor, bovine (*Bos taurus*)	1117	Stem bromelain	37	2	5.4	Low	Milk
24	Myoglobin (*Bos taurus*)	1272	Thermolysin	40	1	2.5	Low	Meat

[a] BIOPEP-UWM database protein ID.

[b] Total number of peptides (≥2 amino acids) generated by *in silico* digestion.

[c] Number of peptides with C-terminal residue (P1’) ∈ {W, Y, F, P}.

[d] Protein source category represented in the rule-development set: Milk = bovine milk protein; Meat = bovine beef protein; Wheat = wheat gluten/gliadin. See Table 1 for the corresponding commercial products.

Table S4. Structural rule frequencies for the 24 protein–enzyme combinations in the rule-development set (Table 1), calculated from in silico enzymatic hydrolysis of full-length protein sequences in the BIOPEP-UWM database.

#	Tertile	Protein	BIOPEP ID	Enzyme	No. of peptides	P1’ freq. (%)	Rule 1 freq. (%)	Rule 2 freq. (%)	Rule 3 freq. (%)	Rule 4 freq. (%)	Rule 5 freq. (%)	ACE Inh. (%)	IC_{50} (mg/mL)
1	High	Lactoferrin	1212	Prolyl oligopeptidase	30	96.67	26.67	10.00	46.67	0.00	43.33	63.3 ± 0.4	0.44 ± 0.00
2		κ-Casein	1117	Prolyl oligopeptidase	20	95.00	30.00	0.00	60.00	10.00	50.00	70.9 ± 0.3	0.39 ± 0.01
3		β-Lactoglobulin A	1851	Prolyl oligopeptidase	9	88.89	22.22	11.11	33.33	11.11	33.33	71.6 ± 0.3	0.24 ± 0.01
4		Myoglobin	1272	Prolyl oligopeptidase	4	75.00	25.00	0.00	75.00	0.00	75.00	96.3 ± 0.0	0.03 ± 0.00
5		α-Lactalbumin	1854	Prolyl oligopeptidase	3	66.67	33.33	0.00	33.33	0.00	33.33	68.8 ± 0.3	0.29 ± 0.00
6		Gluten	1329	Pepsin (pH 1.3)	41	53.66	7.32	0.00	7.32	0.00	7.32	45.3 ± 0.5	0.58 ± 0.00
7		Gliadin	1319	Papain	82	43.90	4.88	0.00	7.32	0.00	6.10	34.8 ± 0.7	0.69 ± 0.00
8		Lactoferrin	1212	Chymotrypsin A	125	37.60	5.60	0.80	12.00	0.00	12.00	66.9 ± 0.3	0.42 ± 0.01
9	Medium	α-Lactalbumin	1854	Chymotrypsin A	28	32.14	7.14	0.00	10.71	0.00	10.71	68.1 ± 0.3	0.41 ± 0.00
10		κ-Casein	1117	Pepsin (pH 1.3)	16	31.25	0.00	0.00	6.25	0.00	6.25	30.4 ± 0.7	0.77 ± 0.01
11		Gluten	1329	Pepsin (pH > 2)	85	30.59	7.06	0.00	7.06	0.00	7.06	36.2 ± 0.6	0.64 ± 0.01
12		Gliadin	1319	Thermolysin	55	29.09	1.82	0.00	5.45	0.00	5.45	23.8 ± 0.8	0.99 ± 0.01
13		Lactoferrin	1212	Pepsin (pH 1.3)	78	25.64	2.56	0.00	8.97	0.00	6.41	44.6 ± 0.6	0.61 ± 0.01
14		Gluten	1329	Stem bromelain	65	24.62	0.00	0.00	0.00	0.00	0.00	16.7 ± 0.8	2.38 ± 0.04
15		β-Lactoglobulin A	1851	Chymotrypsin A	33	24.24	6.06	0.00	15.15	0.00	15.15	86.2 ± 0.1	0.12 ± 0.00
16		Myoglobin	1272	Chymotrypsin A	39	23.08	0.00	0.00	7.69	0.00	7.69	47.7 ± 0.5	0.53 ± 0.00
17	Low	Myoglobin	1272	Papain	40	12.50	0.00	0.00	5.00	0.00	5.00	45.8 ± 0.5	0.59 ± 0.02
18		Lactoferrin	1212	Papain	185	11.89	1.08	0.00	4.32	0.00	2.70	51.9 ± 0.5	0.48 ± 0.01
19		β-Lactoglobulin A	1851	Pepsin (pH > 2)	36	11.11	5.56	0.00	8.33	0.00	5.56	50.2 ± 0.5	0.49 ± 0.02
20		α-Lactalbumin	1854	Thermolysin	30	10.00	0.00	0.00	3.33	0.00	0.00	8.3 ± 0.9	2.74 ± 0.06
21		Gluten	1329	Trypsin	11	9.09	9.09	0.00	9.09	0.00	9.09	76.2 ± 0.2	0.26 ± 0.00
22		Lactoferrin	1212	Thermolysin	144	8.33	0.69	1.39	2.78	0.00	2.78	25.5 ± 0.7	0.94 ± 0.02
23		κ-Casein	1117	Stem bromelain	37	5.41	0.00	0.00	0.00	0.00	0.00	7.8 ± 0.9	2.75 ± 0.02
24		Myoglobin	1272	Thermolysin	40	2.50	2.50	0.00	2.50	2.50	2.50	24.6 ± 0.8	1.55 ± 0.02

P1’ freq., frequency of peptide fragments with C-terminal residue (P1’) ∈ {W, Y, F, P} [6,7]. Rule 1: P1’ ∈ {W, Y, F, P} AND P2’ ∈ {V, L, I} [6,7]. Rule 2 (tripeptide model): P1’ ∈ {W, F, P}

AND P2’ ∈ {K, R} AND P3’ ∈ {V, L, I} [7]. Rule 3: P1’ ∈ {W, Y, F, P} AND P2’ ∈ {L, I, V, K, R, H, W, Y, F} [8]. Rule 4: P1’ ∈ {W, F, Y, H, P} AND P3’ ∈ {W, F, Y} (P2’ = any) [9]. Rule 5 (this study): P1’ ∈ {W, Y, F, P} AND P2’ ∈ {L, I, V, K, R, H}. ACE inhibition (%) at 0.5 mg mL^{-1} and hydrolysate IC_{50} values are experimental results obtained from three independently prepared hydrolysis batches (n = 3). Pearson’s correlation coefficients between rule frequencies and ACE inhibitory activity are reported in Table S5.

Table S5. Internal comparison of structure–activity rules and their Pearson correlations with experimental ACE inhibitory activity and $\log_{10}(1/IC_{50})$ in the 24-hydrolysate derivation set (n = 24).

Rule	Reference	Evidence level	Non-zero (n/24)	P1' condition	P2' condition	P3' condition	r vs ACE Inh. (%) [a]	r vs $\log_{10}(1/IC_{50})$ [a]	Remarks
P1'	This study	This study	24/24	{W,Y,F,P}	—	—	+0.550 **	+0.503 *	Baseline; P1' alone showed limited association with $\log_{10}(1/IC_{50})$
Rule 1	6, 7	Explicit (kinetics/residue-level)	18/24	{W,Y,F,P}	{V,L,I}	—	+0.659 ***	+0.588 **	Branched-chain aliphatic at P2'. Source model R^2 = 0.732 (Wu dipeptide PLS)
Rule 2	7	Explicit (PLS coefficients)	4/24	{W,F,P}	{K,R}	{V,L,I}	+0.234 ns	+0.159 ns	Tripeptide rule; too restrictive (low coverage). Source model R^2 = 0.471 (Wu tripeptide PLS)
Rule 3 [b]	8	Inferred (VIP→property mapping) [b]	22/24	{W,Y,F,P}	{L,I,V,K,R, H,W,Y,F}	—	+0.691 ***	+0.704 ***	P2' includes aromatic residues overlapping with P1'. Source model R^2 = 0.734 (Deng dipeptide PLS+BOSS)
Rule 4	9	Explicit (positional enrichment)	3/24	{W,F,Y,H,P}	Any	{W,F,Y}	+0.253 ns	+0.145 ns	Tripeptide rule; too restrictive (low coverage). Source: docking enrichment (no R^2)
**Rule 5 #**	*This study*	Derived (non-redundant; this study) #	21/24	{W,Y,F,P}	{L,I,V,K,R,H}	—	**+0.711** ***	**+0.741** ***	Highest internal correlation in the derivation set; aromatic residues excluded from P2' to obtain a parsimonious rule

[a] Pearson's product-moment correlation coefficient (n = 24 protein–enzyme combinations) between the rule score and (i) experimental ACE inhibition (%) or (ii) $\log_{10}(1/IC_{50})$. Non-zero (n/24) indicates combinations with at least one matched peptide under the rule (rule score > 0). Significance: ns (p > 0.05); *, p < 0.05; **, p < 0.01; ***, p < 0.001 (two-tailed).

P1', P2', and P3' denote the C-terminal, penultimate, and antepenultimate residue positions of hydrolysis-generated peptides, respectively, following Schechter & Berger [12] nomenclature. —, no constraint applied at this position. P1' row: baseline using only the C-terminal residue. Rules 1–4: literature-derived; Rule 5: this study. Evidence level denotes whether residue sets were explicitly reported at the residue level or inferred from descriptor/VIP-based property preferences.

[b] For Rule 3, the original authors reported structure–activity relationships at the level of physicochemical properties (hydrophobicity, steric bulk, electrostatic charge) rather than enumerating specific amino acid sets. The residue sets shown were inferred by the present authors by mapping the reported property preferences to corresponding amino acid categories.

#, Rule 5 was selected for subsequent evaluation because it showed the highest internal correlation with $\log_{10}(IC_{50})$ in the 24-hydrolysate derivation set while retaining a parsimonious, simplified P2' definition (see section 2.2.4 for rationale).

Table S6. MALDI-TOF/TOF instrument specifications and acquisition parameters.

Parameter	Setting
Instrument	
Manufacturer	Bruker Daltonics (Bremen, Germany)
Instrument type	MALDI-TOF/TOF
Instrument series	Bruker Flex Series (with LIFT capability)
Serial number	247420.00100
Target plate	MTP 384 ground steel (Part No. 0209513, Serial 0020931)
Ion source	Laser desorption / ionization (LD+)
Matrix	α-Cyano-4-hydroxycinnamic acid (HCCA)
Software	
Acquisition software	flexControl 3.0.185.0
Data processing software	flexAnalysis 3.0.96.0
Database search	Mascot (Matrix Science)
MS mode (Reflectron Positive)	
Acquisition method	RP_FingerPrint_small range_20160120.par
Polarity	Positive ion mode
Analyzer mode	Reflectron
Accelerating voltage (IS1)	25 kV
Extraction voltage (IS2)	21.6 kV
Lens voltage	9.9 kV
Reflector voltage	26.3 kV
Detector voltage	1.746 kV
Pulsed ion extraction (PIE) delay	90 ns
Laser attenuation	72–87% (optimized per sample)
Laser focus	Medium (33)
Laser repetition rate	100 Hz
Laser shots accumulated	400–2,600 shots (optimized per sample)
Digitizer sampling rate	2.0 GS/s (dwell time 0.5 ns)
Data points per spectrum	107,862
Matrix suppression (deflection)	Off (DPMASS = 700 Da, DEFLON = no)
Analysis method (flexAnalysis)	PMF.FAMSMethod; baseline subtraction: TopHat; building blocks: Amino Acids (500 ppm tolerance)
MS/MS mode (LIFT, Positive)	
Acquisition method	LIFT_low mass_201606_positive.lft
Fragmentation mode	LIFT (post-source decay with voltage boost)
LIFT 1 voltage	19 kV
LIFT 2 voltage	4.1 kV
Accelerating voltage (IS1)	8 kV
Extraction voltage (IS2)	7.2 kV
Lens voltage	3.6 kV
Reflector voltage	29.5 kV
Detector voltage	1.881 kV
FAST mode	On
Pulsed ion extraction (PIE) delay	Not applied (0 ns)
Laser attenuation	23–68% (optimized per precursor ion)
Laser shots accumulated	200–6,400 shots (optimized per precursor ion)

Digitizer sampling rate	1.0 GS/s (dwell time 1.0 ns)
Data points per spectrum	11,660
Analysis method (flexAnalysis)	SNAP_full_process.FALIFTMethod

Note. All parameters were extracted from the Bruker acqus parameter files embedded in the original .d data directories. Laser attenuation and accumulated shots varied across samples to optimize signal-to-noise ratios; ranges are reported. IS1 and IS2 refer to ion source 1 and 2 voltages, respectively. PIE = pulsed ion extraction; LIFT = Bruker's proprietary post-source decay (PSD) technology with voltage re-acceleration for enhanced fragment ion sensitivity; FAST = fragment analysis and structural TOF; SNAP = sophisticated numerical annotation procedure for peak detection.

Table S7. Molecular docking parameters and grid box coordinates for ACE–peptide docking simulations.

(A) General docking settings

Parameter	Value / Description
Docking software	AutoDock Vina 1.2.7 [17,18]
Scoring function	Vina empirical scoring function [18]
Receptor	Human testicular ACE (tACE, C-domain); PDB ID: 1O86; Resolution: 2.0 Å [19]
Receptor preparation	Lisinopril and crystallographic water molecules removed; catalytic Zn^{2+} ion and two Cl^{-} ions (CL1, CL2) retained; polar hydrogens added; Gasteiger charges assigned; converted to PDBQT via Meeko [23]
Ligand preparation	Peptide 3D conformations generated using PeptideBuilder [22] and energy-minimized with the MMFF94 force field; protonation states assigned at physiological pH; converted to PDBQT via Meeko [23] with RDKit [24] for molecular handling.
Exhaustiveness	128 (16× default; thorough sampling for peptides with multiple rotatable bonds)
Number of binding modes	20
Energy range	3 kcal mol^{-1}
Random seed	42 (for reproducibility)
CPU cores	8
Computing platform	Mac Mini M2 (Apple Silicon, ARM64)

(B) Grid box coordinates for three ACE binding sites

**Binding site**	**Center *x* (Å)**	**Center *y* (Å)**	**Center *z* (Å)**	**Box size (Å^3) / Key residues**
**Site 1 — Catalytic active site** (Zn^{2+}/lisinopril pocket) [19]	41.0	37.0	46.5	30 × 30 × 30<br>Zn^{2+} coordination: His383, His387, Glu411<br>S2: His353, Ala354<br>S1: Glu384, Tyr523, Phe512<br>S1’: Glu162<br>S2’: Gln281, Lys511, Tyr520, His513
**Site 2 — CL1 distal allosteric** (Cl^{-} binding site 1; 20.7 Å from Zn^{2+}) [24]	29.2	28.1	36.2	20 × 20 × 20<br>Key residues: Arg186, Arg489, Trp485, Trp486, Asp507; stabilizes enzyme–substrate complex [24]
**Site 3 — CL2 proximal allosteric** (Cl^{-} binding site 2; 10.4 Å from Zn^{2+}) [24]	36.3	45.1	44.8	20 × 20 × 20<br>Key residues: Arg522, Tyr224, Pro407, Pro519, Ile521; Cl^{-} breaks Arg522–Asp465 salt bridge, repositions Tyr523 for catalysis [24]

Note. Grid centers for Site 1 were determined from the crystallographic position of co-crystallized lisinopril (LPR) in PDB 1O86. Grid centers for Sites 2 and 3 were positioned at the crystallographic Cl^{-} ion coordinates (CL1 [chain A, residue 703]

and CL2 [chain A, residue 704], respectively; Natesh et al., 2003). Box size of 30 × 30 × 30 Å^3 for the catalytic pocket was chosen to encompass all active-site subsites (S2, S1, S1', S2'); 20 × 20 × 20 Å^3 for allosteric chloride channels accommodates the smaller binding cavities. An exhaustiveness of 128 (16× default) was selected to ensure thorough conformational sampling of peptide ligands with multiple rotatable bonds.

Table S8. Predicted Rule 5-compliant peptide frequency (Freq., %) and composition-weighted Rule 5-compliant peptide frequency (Wtd., %) of bovine milk protein subunits hydrolyzed by four proteases, calculated using Rule 5 (P1' ∈ {W, Y, F, P} AND P2' ∈ {L, I, V, K, R, H}). These commercial hydrolysates were not used to modify the Rule 5 residue definitions.

Commercial food protein	Subunits	ID [a]	Rel. abund. (%) [b]	Thermolysin		Pepsin (pH >2)		Papain		Chymotrypsin	
				Rule 5 freq. (%)	Wtd. Rule 5 freq. (%)	Rule 5 freq. (%)	Wtd. Rule 5 freq. (%)	Rule 5 freq. (%)	Wtd. Rule 5 freq. (%)	Rule 5 freq. (%)	Wtd. Rule 5 freq. (%)
Bovine milk powder	αS1-Casein	1086	33.79	6.82	2.30	7.14	2.41	5.56	1.88	8.11	2.74
	αS2-Casein	1090	9.17	4.44	0.41	15.56	1.43	12.73	1.17	18.60	1.71
	β-Casein	1097	27.64	6.00	1.66	1.85	0.51	2.13	0.59	7.50	2.07
	κ-Casein	1117	11.06	14.63	1.62	6.52	0.72	4.17	0.46	9.38	1.04
	α-Lactalbumin	1115	4.40	0.00	0.00	3.33	0.15	6.45	0.28	9.68	0.43
	β-Lactoglobulin	1821	12.31	2.70	0.33	5.56	0.68	6.67	0.82	15.15	1.87
	Serum albumin	1729	1.51	2.26	0.03	4.55	0.07	5.10	0.08	10.17	0.15
	Lactoferrin	1212	0.13	2.78	0.00	6.25	0.01	2.70	0.00	12.00	0.02
	**Wtd. average (%)**				**6.36**		**5.98**		**5.28**		**10.02**
	**ACE inh. (%)**				**61.7 ± 0.7**		**28.4 ± 1.7**		**9.4 ± 0.8**		**88.7 ± 0.2**
	**IC_{50} (mg mL^{-1})**				**0.39 ± 0.01**		**0.92 ± 0.03**		**2.78 ± 0.05**		**0.18 ± 0.00**

[a] ID: protein accession number in the BIOPEP-UWM database.

[b] Rel. abund., relative abundance: literature-reported mass fraction (%, w/w) of each listed subunit in total milk protein [S1]. Values shown here were normalized to 100% over listed subunits for weighting; minor proteins not listed.

Rule 5 freq. (%) = (No. of peptides with P1' ∈ {W, Y, F, P} AND P2' ∈ {L, I, V, K, R, H}) / (Total fragments ≥ 2 aa) × 100.

Wtd. Rule 5 freq. (%) = (Rel. abund. / Σ Rel. abund.) × Freq. Composition-weighted average = Σ Wtd. freq. Values rounded to 2 decimals; totals may differ due to rounding.

ACE inh. (%) at 0.5 mg mL^{-1} and IC_{50} determined experimentally for the mixed hydrolysate at the indicated enzyme (see Section 2.5).

Table S9. Predicted Rule 5-compliant peptide frequency (Freq., %) and composition-weighted Rule 5-compliant peptide frequency (Wtd., %) of beef protein subunits hydrolyzed by four proteases, calculated using Rule 5 (P1' ∈ {W, Y, F, P} AND P2' ∈ {L, I, V, K, R, H}). These commercial hydrolysates were not used to modify the Rule 5 residue definitions.

Commercial food protein	Subunits	ID [a]	Rel. abund. (%) [b]	Thermolysin		Pepsin (pH >2)		Papain		Chymotrypsin	
				Rule 5 freq. (%)	Wtd. Rule 5 freq. (%)	Rule 5 freq. (%)	Wtd. Rule 5 freq. (%)	Rule 5 freq. (%)	Wtd. Rule 5 freq. (%)	Rule 5 freq. (%)	Wtd. Rule 5 freq. (%)
Beef meat	Myosin light chain	1271	6.10	0.00	0.00	6.45	0.39	4.88	0.30	2.78	0.17
	Myosin heavy chain	1270	30.49	0.00	0.00	8.57	2.61	6.38	1.95	9.09	2.77
	Actin	1277	14.63	3.66	0.54	6.74	0.99	1.11	0.16	9.46	1.38
	Troponin-c	1138	2.44	1.75	0.04	2.04	0.05	1.61	0.04	9.09	0.22
	Hemoglobin α	1233	0.73	0.00	0.00	2.94	0.02	5.00	0.04	5.88	0.04
	Hemoglobin β	1227	0.73	0.00	0.00	11.76	0.09	0.00	0.00	12.12	0.09
	Myoglobin	1272	2.20	2.50	0.05	3.23	0.07	5.00	0.11	7.69	0.17
	Collagen α-1	1111	36.59	0.00	0.00	0.00	0.00	0.00	0.00	0.00	0.00
	Elastin	1107	6.10	4.39	0.27	1.89	0.12	4.76	0.29	10.00	0.61
	**Wtd. average (%)**				**0.90**		**4.34**		**2.88**		**5.46**
	**ACE inh. (%)**				**20.4 ± 1.7**		**15.9 ± 1.5**		**18.7 ± 1.6**		**78.6 ± 0.2**
	**IC_{50} (mg mL^{-1})**				**1.26 ± 0.03**		**1.43 ± 0.02**		**1.33 ± 0.01**		**0.28 ± 0.00**

[a] ID: protein accession number in the BIOPEP-UWM database.

[b] Rel. abund., relative abundance: literature-compiled approximate mass fraction (%, w/w) of each listed subunit in total beef protein [S2,S3]. Values shown here were normalized to 100% over listed subunits for weighting; minor proteins not listed. Representative values may vary by muscle type, breed, and age.

Rule 5 freq. (%) = (No. of peptides with P1' ∈ {W, Y, F, P} AND P2' ∈ {L, I, V, K, R, H}) / (Total fragments ≥ 2 aa) × 100.

Wtd. Rule 5 freq. (%) = (Rel. abund. / Σ Rel. abund.) × Freq. Composition-weighted average = Σ Wtd. freq. Values rounded to 2 decimals; totals may differ due to rounding.

ACE inh. (%) at 0.5 mg mL^{-1} and IC_{50} determined experimentally for the mixed hydrolysate at the indicated enzyme (see Section 2.5).

Table S10. Predicted Rule 5-compliant peptide frequency (Freq., %) and composition-weighted Rule 5-compliant peptide frequency (Wtd., %) of wheat gluten protein subunits hydrolyzed by four proteases, calculated using Rule 5 (P1' ∈ {W, Y, F, P} AND P2' ∈ {L, I, V, K, R, H}). These commercial hydrolysates were not used to modify the Rule 5 residue definitions.

Commercial food protein	Subunits	ID [a]	Rel. abund. (%) [b]	Thermolysin		Pepsin (pH >2)		Papain		Chymotrypsin	
				**Rule 5 freq. (%)**	**Wtd. Rule 5 freq. (%)**	**Rule 5 freq. (%)**	**Wtd. Rule 5 freq. (%)**	**Rule 5 freq. (%)**	**Wtd. Rule 5 freq. (%)**	**Rule 5 freq. (%)**	**Wtd. Rule 5 freq. (%)**
Wheat gluten	α/β-Gliadin	1422	30.00	9.43	2.83	4.41	1.32	4.41	1.32	4.00	1.20
	γ-Gliadin	1319	27.00	5.45	1.47	2.25	0.61	6.10	1.65	6.38	1.72
	ω-Gliadin	1418	10.00	4.08	0.41	2.25	0.23	3.80	0.38	2.17	0.22
	Glutenin (HMW)	1342	12.00	7.89	0.95	8.33	1.00	4.76	0.57	8.70	1.04
	Glutenin (LMW)	1333	21.00	6.45	1.35	7.41	1.56	4.23	0.89	7.69	1.61
	**Wtd. average (%)**				**7.01**		**4.71**		**4.81**		**5.80**
	**ACE inh. (%)**				**37.9 ± 0.8**		**68.7 ± 0.6**		**11.8 ± 1.2**		**39.7 ± 1.0**
	**IC_{50} (mg mL^{-1})**				**0.68 ± 0.01**		**0.36 ± 0.00**		**2.34 ± 0.04**		**0.67 ± 0.01**

[a] ID: protein accession number in the BIOPEP-UWM database.

[b] Rel. abund., relative abundance: wheat gliadin-type composition (%, w/w), normalized to 100% over α/β-, γ-, and ω-gliadins based on the qualitative gluten protein classification described by Wieser [S4]; representative proportions may vary with cultivar and growth conditions.

Rule 5 freq. (%) = (No. of peptides with P1' ∈ {W, Y, F, P} AND P2' ∈ {L, I, V, K, R, H}) / (Total fragments ≥ 2 aa) × 100.

Wtd. Rule 5 freq. (%) = (Rel. abund. / Σ Rel. abund.) × Freq. Composition-weighted average = Σ Wtd. freq. Values rounded to 2 decimals; totals may differ due to rounding.

ACE inh. (%) at 0.5 mg mL^{-1} and IC_{50} determined experimentally for the mixed hydrolysate at the indicated enzyme (see Section 2.5).

Table S11. Predicted Rule 5-compliant peptide frequency (Freq., %) and composition-weighted Rule 5-compliant peptide frequency (Wtd., %) of wheat gliadin protein subunits hydrolyzed by four proteases, calculated using Rule 5 (P1' ∈ {W, Y, F, P} AND P2' ∈ {L, I, V, K, R, H}). These commercial hydrolysates were not used to modify the Rule 5 residue definitions.

Commercial food protein	Subunits	ID [a]	Rel. abund. (%) [b]	Thermolysin		Pepsin (pH >2)		Papain		Chymotrypsin	
				Rule 5 freq. (%)	Wtd. Rule 5 freq. (%)	Rule 5 freq. (%)	Wtd. Rule 5 freq. (%)	Rule 5 freq. (%)	Wtd. Rule 5 freq. (%)	Rule 5 freq. (%)	Wtd. Rule 5 freq. (%)
Wheat gliadin	α/β-Gliadin	1422	45.92	9.43	4.33	4.41	2.03	4.41	2.03	4.00	1.84
	γ-Gliadin	1319	40.82	5.45	2.22	2.25	0.92	6.10	2.49	6.38	2.60
	ω-Gliadin	1418	13.27	4.08	0.54	2.25	0.30	3.80	0.50	2.17	0.29
	**Wtd. average (%)**				**7.10**		**3.24**		**5.02**		**4.73**
	**ACE inh. (%)**				**47.6 ± 0.3**		**25.7 ± 2.0**		**17.1 ± 0.6**		**30.1 ± 1.6**
	**IC_{50} (mg mL^{-1})**				**0.53 ± 0.01**		**0.94 ± 0.01**		**1.36 ± 0.01**		**0.77 ± 0.01**

[a] ID: protein accession number in the BIOPEP-UWM database.

[b] Rel. abund., relative abundance: wheat gliadin-type composition (%, w/w), normalized to 100% over α/β-, γ-, and ω-gliadins based on the qualitative gluten protein classification described by Wieser [S4]; representative proportions may vary with cultivar and growth conditions.

Rule 5 freq. (%) = (No. of peptides with P1' ∈ {W, Y, F, P} AND P2' ∈ {L, I, V, K, R, H}) / (Total fragments ≥ 2 aa) × 100.

Wtd. Rule 5 freq. (%) = (Rel. abund. / Σ Rel. abund.) × Freq. Composition-weighted average = Σ Wtd. freq. Values rounded to 2 decimals; totals may differ due to rounding.

ACE inh. (%) at 0.5 mg mL^{-1} and IC_{50} determined experimentally for the mixed hydrolysate at the indicated enzyme (see Section 2.5).

Table S12. Mass-spectrometric peptide assignments from protein hydrolysates of the 24 protein–enzyme combinations, supported by protease-specific virtual digestion.

No.	Peptide sequence	Length (aa)	Protein source	Protein subunit	Enzyme	Calc. M (Da)	Calc. [M+H]$^{+}$ (m/z)	Obs. feature m/z	Ion type	P1’	P2’	Rule 5 compliant
**1**	**VF**	**2**	**Wheat gluten**	**Gluten**	**Pepsin (pH 1.3)**	**264.15**	**265.15**	**265.99**	Putative low-mass feature	**F**	**V**	**Yes**
**2**	**GIF**	**3**	**Wheat gluten**	**Gluten**	**Pepsin (pH 1.3); Pepsin (pH > 2.0)**	**335.18**	**336.19**	**335.37**	Putative low-mass feature	**F**	**I**	**Yes**
**3**	**LP**	**2**	**Wheat gluten**	**Gluten**	**Bromelain**	**228.15**	**229.15**	**228.07**	Putative low-mass feature	**P**	**L**	**Yes**
**4**	**IP**	**2**	**Wheat gluten**	**Gluten**	**Bromelain**	**228.15**	**229.15**	**228.07**	Putative low-mass feature	**P**	**I**	**Yes**
**5**	**VP**	**2**	**Wheat gluten**	**Gluten**	**Bromelain**	**214.13**	**215.14**	**213.12**	Putative low-mass feature	**P**	**V**	**Yes**
6	FSSSR	5	Bovine milk	α-Lactalbumin	Thermolysin	582.28	583.28	583.31	[M+H]$^{+}$	R	S	No
7	KNPFFYR	7	Wheat gliadin	Gliadin	Papain	970.50	971.51	971.46	[M+H]$^{+}$	R	Y	No
8	FWLRRT	6	Bovine milk	Lactoferrin	Thermolysin	877.49	878.50	878.42	[M+H]$^{+}$	T	R	No
9	KFYKHR	6	Bovine milk	β-Lactoglobulin A	Pepsin (pH > 2.0)	877.49	878.50	878.42	[M+H]$^{+}$	R	H	No
10	KTPPHHA	7	Bovine milk	β-Lactoglobulin A	Pepsin (pH > 2.0)	786.41	787.42	787.44	[M+H]$^{+}$	A	H	No

11	NVLKK	5	Bovine milk	β-Lactoglobulin A	Pepsin (pH > 2.0)	600.40	601.40	601.36	$[M+H]^+$	K	K	No
12	TPKDKLGMLKLHR	13	Bovine milk	β-Lactoglobulin A	Pepsin (pH > 2.0)	1535.90	1536.90	1536.98	$[M+H]^+$	R	H	No
13	HDDKPHPH	8	Bovine milk	κ-Casein	Pepsin (pH 1.3)	981.44	982.45	982.48	$[M+H]^+$	H	P	No
14	NLHHPYD	7	Bovine milk	κ-Casein	Pepsin (pH 1.3)	894.40	895.41	895.40	$[M+H]^+$	D	Y	No
15	NLHPHPH	7	Bovine milk	κ-Casein	Pepsin (pH 1.3)	850.42	851.43	851.44	$[M+H]^+$	H	P	No
16	YHPYKN	6	Bovine milk	κ-Casein	Pepsin (pH 1.3)	820.39	821.39	821.39	$[M+H]^+$	N	K	No
17	GTKYTHRT	8	Equine skeletal muscle	Myoglobin	Thermolysin	962.49	963.50	963.51	$[M+H]^+$	T	R	No
18	HGHRPPFT	8	Equine skeletal muscle	Myoglobin	Thermolysin	947.47	948.48	948.23	$[M+H]^+$	T	F	No
19	KCHHFPPR	8	Equine skeletal muscle	Myoglobin	Thermolysin	1020.51	1021.51	1021.55	$[M+H]^+$	R	P	No
20	KASTKFPYR	9	Equine skeletal muscle	Myoglobin	Papain	1096.60	1097.61	1097.64	$[M+H]^+$	R	Y	No
21	VPRKFKRNFYLCT	13	Equine skeletal muscle	Myoglobin	Papain	1670.91	1671.92	1672.08	$[M+H]^+$	T	C	No

Calc. M, neutral monoisotopic mass calculated from amino acid residue masses. Calc. $[M+H]^+$, theoretical protonated molecular ion (M + 1.00728 Da). Obs. feature m/z, observed MALDI-TOF/TOF feature. For entries 1–5, the low-mass features were not treated as accurate $[M+H]^+$ precursor matches because of matrix interference and limited mass accuracy; these sequences are therefore putative candidates supported jointly by virtual digestion and low-mass spectral features. Entries 6–21 showed acceptable precursor-mass agreement.

P1', C-terminal residue; P2', penultimate C-terminal residue (ACE-QSAR convention [6,7]).

Rule 5-compliant: P1' ∈ {W, Y, F, P} AND P2' ∈ {L, I, V, K, R, H}.

LP and IP share identical molecular formulae ($C_{11}H_{20}N_2O_3$; M = 228.15 Da) and cannot be distinguished by precursor mass. The observed low-mass feature is therefore reported as an unresolved LP/IP-associated feature; virtual digestion identifies LP and IP only as alternative candidate sequences and does not establish that either or both were present.

Peptides 1–5 (shaded rows) are Rule 5-compliant and were selected because they represented the intersection of virtual cleavage prediction, Rule 5 compliance, and corresponding candidate-associated low-mass MALDI features; they were subsequently examined by IC_{50} determination, enzyme kinetics, and molecular docking.

Table S13. Database overlap analysis: effect of excluding Wu et al. [7] training peptides on Rule 5 enrichment significance.

Length window	n (full)	n (excl.)	z (full)	p (full)	z (excl.)	p (excl.)	Sig.?
2–2 aa	112	97	−4.329	< 0.001	−4.095	< 0.001	Yes
2–3 aa	470	402	−4.259	< 0.001	−3.006	0.003	Yes
2–4 aa	631	519	−2.991	0.003	−1.643	0.100	No
2–5 aa	726	604	−1.946	0.052	−1.104	0.270	No
2–15 aa	1429	1274	−0.947	0.344	−0.551	0.582	No

Full: all 1,429 peptides from AHTPDB, BIOPEP-UWM, and MBPDB. Excl.: 155 peptides overlapping with the Wu et al. [7] training set removed (n = 1,274). z: Mann–Whitney U test statistic. Sig.: significant at $p < 0.05$ (two-tailed).

Table S14. Model selection for ACE inhibition kinetics: competitive vs. mixed-type inhibition assessed by corrected Akaike Information Criterion (AICc) and F-test.

Peptide	P1’	AICc (Comp.)	AICc (Mixed)	ΔAICc	F-stat	*p*-value	Preferred model
**VF**	F	245.8	164.3	+81.5	98.2	< 0.0001	**Mixed**
**GIF**	F	289.1	183.7	+105.4	132.7	< 0.0001	**Mixed**
**LP**	P	156.2	160.6	−4.4	1.8	0.21	**Competitive**
**IP**	P	142.7	163.9	−21.2	0.4	0.55	**Competitive**
**VP**	P	168.4	187.1	−18.7	0.7	0.43	**Competitive**

ΔAICc = AICc(competitive) − AICc(mixed). Positive ΔAICc favors the mixed-type model; negative ΔAICc favors the competitive model. A difference of |ΔAICc| > 10 indicates strong evidence. F-test compares the nested competitive model (1 parameter: K_i) against the mixed-type model (2 parameters: K_i, K_i’). P1’: C-terminal residue of the inhibitory peptide.

Table S15. Benjamini–Hochberg false-discovery-rate (BH-FDR) corrected p-values for Mann–Whitney U tests across all 87 pairwise length windows.

LB (aa)	UB (aa)	z-score	p (raw)	p (BH-FDR)	p (Bonf.)	Sig. (BH)
2	2	-4.329	1.50e-5	8.93e-4	0.0013	***
2	3	-4.259	2.05e-5	8.93e-4	0.0018	***
2	4	-2.991	0.0028	0.0806	0.2419	**ns**
2	5	-1.946	0.0517	0.1798	1.0000	**ns**
2	6	-2.429	0.0151	0.1221	1.0000	**ns**
2	7	-1.821	0.0686	0.1990	1.0000	**ns**
2	8	-1.326	0.1848	0.3574	1.0000	**ns**
2	9	-1.095	0.2735	0.4548	1.0000	**ns**
2	10	-1.014	0.3106	0.5004	1.0000	**ns**
2	11	-0.879	0.3794	0.5381	1.0000	**ns**
2	12	-0.800	0.4237	0.5421	1.0000	**ns**
2	13	-0.824	0.4099	0.5397	1.0000	**ns**
2	14	-0.869	0.3848	0.5381	1.0000	**ns**
2	15	-0.853	0.3937	0.5381	1.0000	**ns**
3	3	-1.896	0.0580	0.1849	1.0000	**ns**
3	4	-0.627	0.5307	0.5919	1.0000	**ns**
3	5	0.176	0.8603	0.8703	1.0000	**ns**
3	6	-0.587	0.5572	0.6060	1.0000	**ns**
3	7	-0.048	0.9617	0.9617	1.0000	**ns**
3	8	0.399	0.6899	0.7231	1.0000	**ns**
3	9	0.597	0.5505	0.6060	1.0000	**ns**
3	10	0.635	0.5254	0.5919	1.0000	**ns**
3	11	0.752	0.4521	0.5534	1.0000	**ns**
3	12	0.814	0.4156	0.5397	1.0000	**ns**
3	13	0.773	0.4395	0.5463	1.0000	**ns**
3	14	0.721	0.4709	0.5534	1.0000	**ns**
3	15	0.731	0.4648	0.5534	1.0000	**ns**
4	4	0.838	0.4020	0.5381	1.0000	**ns**
4	5	1.946	0.0517	0.1798	1.0000	**ns**
4	6	0.740	0.4593	0.5534	1.0000	**ns**
4	7	1.374	0.1694	0.3350	1.0000	**ns**
4	8	1.869	0.0616	0.1849	1.0000	**ns**
4	9	2.081	0.0374	0.1628	1.0000	**ns**
4	10	2.091	0.0365	0.1628	1.0000	**ns**
4	11	2.225	0.0261	0.1487	1.0000	**ns**
4	12	2.301	0.0214	0.1432	1.0000	**ns**

4	13	2.252	0.0243	0.1487	1.0000	**ns**
4	14	2.170	0.0300	0.1487	1.0000	**ns**
4	15	2.188	0.0287	0.1487	1.0000	**ns**
5	5	1.419	0.1559	0.3290	1.0000	**ns**
5	6	-0.181	0.8564	0.8703	1.0000	**ns**
5	7	0.568	0.5700	0.6123	1.0000	**ns**
5	8	1.166	0.2436	0.4416	1.0000	**ns**
5	9	1.410	0.1585	0.3290	1.0000	**ns**
5	10	1.409	0.1588	0.3290	1.0000	**ns**
5	11	1.547	0.1219	0.2865	1.0000	**ns**
5	12	1.630	0.1031	0.2638	1.0000	**ns**
5	13	1.571	0.1162	0.2808	1.0000	**ns**
5	14	1.500	0.1336	0.2981	1.0000	**ns**
5	15	1.507	0.1318	0.2981	1.0000	**ns**
6	6	-1.722	0.0851	0.2243	1.0000	**ns**
6	7	-0.532	0.5947	0.6310	1.0000	**ns**
6	8	0.329	0.7422	0.7687	1.0000	**ns**
6	9	0.671	0.5022	0.5749	1.0000	**ns**
6	10	0.711	0.4771	0.5534	1.0000	**ns**
6	11	0.882	0.3778	0.5381	1.0000	**ns**
6	12	0.988	0.3232	0.5112	1.0000	**ns**
6	13	0.918	0.3586	0.5381	1.0000	**ns**
6	14	0.845	0.3981	0.5381	1.0000	**ns**
6	15	0.849	0.3959	0.5381	1.0000	**ns**
7	7	1.614	0.1065	0.2648	1.0000	**ns**
7	8	2.344	0.0191	0.1383	1.0000	**ns**
7	9	2.552	0.0107	0.1221	0.9318	**ns**
7	10	2.422	0.0154	0.1221	1.0000	**ns**
7	11	2.590	0.0096	0.1221	0.8350	**ns**
7	12	2.699	0.0070	0.1221	0.6051	**ns**
7	13	2.586	0.0097	0.1221	0.8447	**ns**
7	14	2.442	0.0146	0.1221	1.0000	**ns**
7	15	2.455	0.0141	0.1221	1.0000	**ns**
8	8	1.755	0.0793	0.2155	1.0000	**ns**
8	9	2.000	0.0455	0.1721	1.0000	**ns**
8	10	1.799	0.0720	0.2021	1.0000	**ns**
8	11	2.020	0.0434	0.1716	1.0000	**ns**
8	12	2.160	0.0308	0.1487	1.0000	**ns**
8	13	2.053	0.0401	0.1660	1.0000	**ns**
8	14	1.876	0.0607	0.1849	1.0000	**ns**

8	15	1.900	0.0574	0.1849	1.0000	**ns**
9	10	0.778	0.4366	0.5463	1.0000	**ns**
9	11	1.144	0.2526	0.4445	1.0000	**ns**
9	12	1.380	0.1676	0.3350	1.0000	**ns**
9	13	1.273	0.2030	0.3840	1.0000	**ns**
9	14	1.125	0.2606	0.4445	1.0000	**ns**
9	15	1.130	0.2585	0.4445	1.0000	**ns**
10	12	1.220	0.2225	0.4118	1.0000	**ns**
10	13	1.087	0.2770	0.4548	1.0000	**ns**
10	14	0.847	0.3970	0.5381	1.0000	**ns**
10	15	0.858	0.3909	0.5381	1.0000	**ns**

LB: lower bound of peptide length window; UB: upper bound. z-score: Mann–Whitney U test statistic comparing IC_{50} distributions of Rule 5–compliant vs. non-compliant peptides. p (raw): uncorrected two-tailed *p*-value. p (BH-FDR): Benjamini–Hochberg adjusted *p*-value across 87 tests. *p* (Bonf.): Bonferroni adjusted *p*-value. Sig. (BH): *** $p_{adj} < 0.001$, ** $p_{adj} < 0.01$, * $p_{adj} < 0.05$, ns = not significant. Green shading indicates windows surviving BH-FDR correction at $\alpha = 0.05$. Negative z-scores indicate Rule 5 enrichment (compliant peptides more potent); positive z-scores indicate depletion.

Table S16. Pearson correlations of Rules 1–5 composition-weighted frequencies with experimental ACE inhibitory activity in the 16 independent commercial-hydrolysate evaluation set (n = 16).

Rule	P1' set	P2' set	r (ACE Inh.)	*p*	r ($\log_{10}(1/IC_{50})$)	*p*
P1' freq.	{W,Y,F,P}	—	+0.174	0.518	+0.202	0.454
Rule 1	{W,Y,F,P}	{V,L,I}	+0.195	0.470	+0.233	0.385
Rule 2	{W,F,P}	{K,R}; P3' {V,L,I}	−0.028	0.919	−0.011	0.969
Rule 3	{W,Y,F,P}	{L,I,V,K,R,H,W,Y,F}	+0.418	0.108	+0.372	0.156
Rule 4	{W,F,Y,H,P}	—	+0.303	0.253	+0.319	0.228
**Rule 5 #**	**{W,Y,F,P}**	**{L,I,V,K,R,H}**	**+0.609 ***	**0.012**	**+0.582 ***	**0.018**

Selected for subsequent analyses. * $p < 0.05$. Frequencies calculated as composition-weighted averages from BIOPEP-UWM virtual hydrolysis (Tables S8–S11). Rule 5 is the only rule achieving significance in the independent set.

Table S17. Sensitivity analyses for the association between composition-weighted Rule 5-compliant peptide frequency and ACE-inhibitory outcomes in the 16 commercial hydrolysates.

Analysis	Outcome	Result	Interpretation
Leave-one-protein-out	ACE inhibition	Pearson r = 0.423–0.697	Significant in 3 of 4 exclusions; removal of milk reduced significance
Leave-one-protein-out	$\log_{10}(1/IC_{50})$	Pearson r = 0.438–0.658	Significant in 3 of 4 exclusions; removal of milk reduced significance
Leave-one-protease-out	ACE inhibition	Pearson r = 0.414–0.673	Removal of chymotrypsin reduced significance
Leave-one-protease-out	$\log_{10}(1/IC_{50})$	Pearson r = 0.368–0.702	Removal of chymotrypsin reduced significance
Partial correlation controlling for protein source and protease identity	ACE inhibition	r = 0.495, p = 0.051 (df = 8)	Marginal association; limited power
Partial correlation controlling for protein source and protease identity	$\log_{10}(1/IC_{50})$	r = 0.518, p = 0.040 (df = 8)	Significant association; interpret cautiously because n = 16

ESI References

S1 Farrell, H. M., Jimenez-Flores, R., Bleck, G. T., Brown, E. M., Butler, J. E., Creamer, L. K., Hicks, C. L., Hollar, C. M., Ng-Kwai-Hang, K. F., & Swaisgood, H. E. (2004). Nomenclature of the proteins of cows' milk—sixth revision. *Journal of Dairy Science*, *87*(6), 1641–1674. https://doi.org/10.3168/jds.S0022-0302(04)73319-6

S2 Zarkadas, C. G., Karatzas, C. N., Khalili, A. D., Khanizadeh, S., & Moates, G. (1988). Quantitative determination of the myofibrillar proteins and connective tissue content in selected porcine skeletal muscles. *Journal of Agricultural and Food Chemistry*, *36*(6), 1131–1146. https://doi.org/10.1021/jf00084a004

S3 Lawrie, R. A. & Ledward, D. A. (2006). Lawrie's Meat Science, Woodhead Publishing, Cambridge, 7th ed.

S4 Wieser, H. (2007). Chemistry of gluten proteins. *Food Microbiology*, *24*(2), 115–119. https://doi.org/10.1016/j.fm.2006.07.004
